# Electronic Coupling in 1,2,3-Triazole Bridged Ferrocenes and Its Impact on Reactive Oxygen Species Generation and Deleterious Activity in Cancer Cells

**DOI:** 10.1021/acs.inorgchem.2c01110

**Published:** 2022-06-14

**Authors:** Przemysław Biegański, Eduard Kovalski, Noel Israel, Evgenia Dmitrieva, Damian Trzybiński, Krzysztof Woźniak, Valerije Vrček, Martina Godel, Chiara Riganti, Joanna Kopecka, Heinrich Lang, Konrad Kowalski

**Affiliations:** † Department of Organic Chemistry, Faculty of Chemistry, University of Łódź, Tamka 12, 91-403 Łódź, Poland; ‡ Institut für Chemie, Anorganische Chemie, Fakultät für Naturwissenschaften, Technische Universität Chemnitz, Straße der Nationen 62, D-09107 Chemnitz, Germany; § Leibniz Institute for Solid State and Materials Research (IFW Dresden), Helmholtzstraße 20, D-01069 Dresden, Germany; ∥ Faculty of Chemistry, Biological and Chemical Research Centre, University of Warsaw, Żwirki i Wigury 101, 02-089 Warszawa, Poland; ⊥ Department of Organic Chemistry, Faculty of Pharmacy and Biochemistry, University of Zagreb, 10000 Zagreb, Croatia; # Department of Oncology, University of Torino, via Santena 5/bis, 10126 Turin, Italy; ∇ MAIN Research Center, Technische Universität Chemnitz, Rosenbergstraße 6, 09126 Chemnitz, Germany

## Abstract

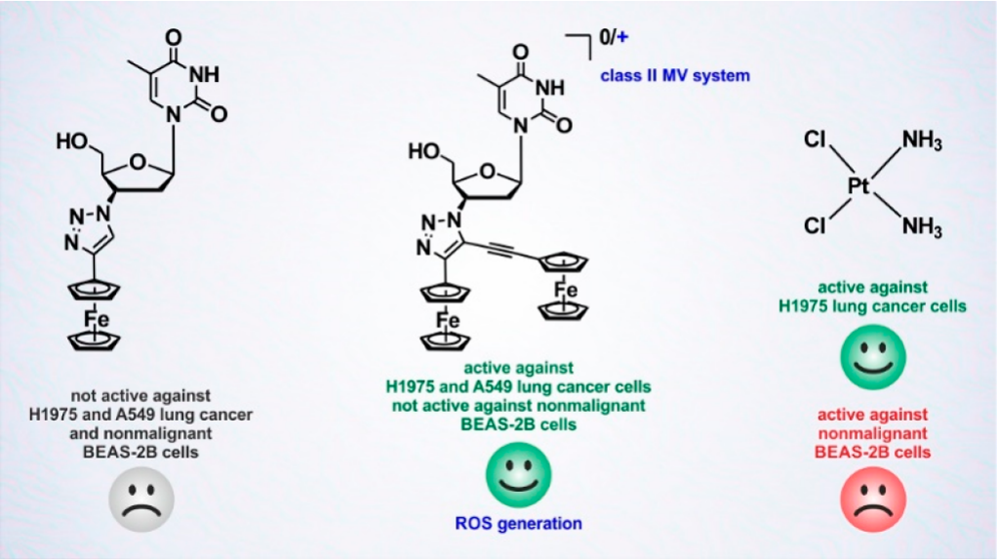

Mixed-valence (MV) binuclear ferrocenyl compounds have long been studied as models for testing theories of electron transfer and in attempts to design molecular-scale electronic devices (*e.g*., molecular wires). In contrary to that, far less attention has been paid to MV binuclear ferrocenes as anticancer agents. Herein, we discuss the synthesis of six 1,2,3-triazole ferrocenyl compounds for combined (spectro)electrochemical, electron paramagnetic resonance (EPR), computational, and anticancer activity studies. Our synthetic approach was based on the copper-catalyzed 1,3-dipolar azide–alkyne cycloaddition reaction and enabled us to obtain in one step compounds bearing either one, two, or three ferrocenyl entities linked to the common 1,2,3-triazole core. Thus, two series of complexes were obtained, which pertain to derivatives of 3′-azido-3′-deoxythymidine (AZT) and 3-azidopropionylferrocene, respectively. Based on the experimental and theoretical data, the two mono-oxidized species corresponding to binuclear AZT and trinuclear 3-azidopropionylferrocene complexes have been categorized as class II mixed-valence according to the classification proposed by Robin and Day. Of importance is the observation that these two compounds are more active against human A549 and H1975 non-small-cell lung cancer cells than their congeners, which do not show MV characteristics. Moreover, the anticancer activity of MV species competes or surpasses, dependent on the cell line, the activity of reference anticancer drugs such as cisplatin, tamoxifen, and 5-fluorouracil. The most active from the entire series of compounds was the binuclear thymidine derivative with the lowest IC_50_ value of 5 ± 2 μM against lung H1975 cancer cells. The major mechanism of antiproliferative activity for the investigated MV compounds is based on reactive oxygen species generation in cancer cells. This hypothesis was substantiated by EPR spin-trapping experiments and the observation of decreased anticancer activity in the presence of *N*-acetyl cysteine (NAC) free-radical scavenger.

## Abstract 2

The 1,2,3-triazole bridged bi- and triferrocenyl compounds were prepared *via* a “click” reaction. Their corresponding mono-oxidized forms have been categorized as class II MV species. The biferrocenyl thymidine derivative showed remarkable anticancer activity against human A549 and H1975 cancer cells and negligible activity against nonmalignant human BEAS-2B cells. The anticancer activity mechanism is mainly due to ROS generation, and it originates from the combination of electronic coupling and the thymidine moiety, combined all together in one molecular scaffold.

## Introduction

Mixed-valence (MV) species derived from d-transition-metal complexes are fascinating objects for chemical and spectroscopic studies. In particular, they are attractive from the perspective of basic studies on electron transfer processes as well as investigation of magnetic exchange interaction phenomena.^1−9^ Moreover, MV compounds are considered to be a source of components and devices for the emerging field of molecular electronics.^7,8,10−13^ The rate of electron delocalization (electronic coupling or communication) in MV species can be examined by a variety of analytical techniques including electrochemistry, ultraviolet/visible (UV–vis) spectroscopy, near-infrared (NIR) spectroscopy, electron paramagnetic resonance (EPR), and Mössbauer spectroscopy.^14−16^ Each of them operates in different time scale. Therefore, to accurately assess the extent of electron delocalization, a combination of slower (EPR and Mössbauer) and faster (UV–vis/NIR) techniques is desirable. Accessible with electrochemical measurements, half-wave potential splitting (Δ*E*_1/2_) often provides a misleading approximation of the amount of electronic coupling in MV compounds.^14^ A much more reliable measure of electron coupling in MV systems is provided by the electronic coupling matrix element *H*_ab_ (*V*_ab_). *H*_ab_ can be determined from the intervalence charge transfer (IVCT) band and using Hush’s two-state model according to eq 1S (see the Supporting Information (SI)).^17,18^ According to the classification of Robin and Day, there are three classes of MV compounds.^19^ Class I comprises valence-trapped systems, class II comprises weakly coupled systems, and class III comprises valence delocalized systems. In fully delocalized class III systems, the electronic coupling matrix element *H*_ab_ is half the energy at the IVCT band maximum, whereas in class I compounds, the IVCT band is not present.

Reported in 1951, ferrocene (FcH = Fe(η^5^-C_5_H_5_)_2_) has become a cornerstone of modern organometallic chemistry.^20,21^ In the last 71 years, ferrocenyl (Fc) compounds have found many applications in catalysis, biology, materials chemistry, and so forth.^22−35^ One of the reasons behind this success is due to the electrochemical properties of ferrocene and its derivatives. The Fc/[Fc]^+^ redox couple is usually characterized by superb chemical reversibility combined with great thermal stability.^36^ Thus, compounds containing Fc groups linked by aromatic or π-electron cyclic or acyclic bridges have been recognized as a source of MV species that are nicely suited for electronic communication studies.^37^ In this respect, bridges such as benzene,^38,39^ pyridine,^40^ 1,3,5-triazine,^40^ pyrrole,^41−43^ thiophene,^44−48^ selenophene,^49^ thiadiazole,^48^ thiazole,^50^ phosphole,^51,52^ and silole,^53^ to name just a few, have been studied.

The Fc/[Fc]^+^ redox couple has also found numerous applications in biology. It can be tentatively categorized as analytical and therapeutic. Regarding the former, adequately designed ferrocenylated DNA oligomers have been applied for single-base mismatches^54^ and viral DNA^55^ electrochemical detection as well as for redox coding of nucleobases and their ratiometric sensing.^56^ The role of redox chemistry in therapeutic applications of ferrocene derivatives is exemplified by a family of ferrocifen drugs.^33,57^ The mechanism of action of these remarkably anticancer-active compounds begins with single oxidation of the Fc entity, which is embedded in the “ferrocenyl-ene-phenol” structural motif.

**Figure 1:**
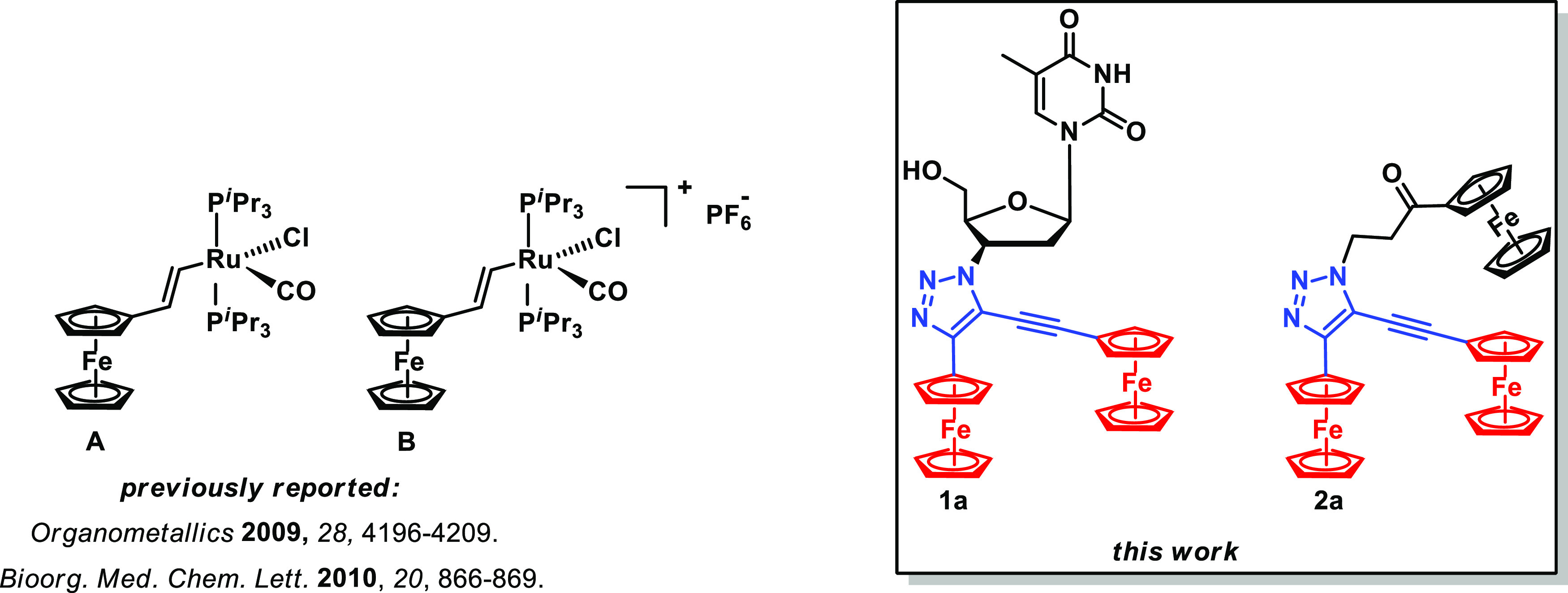
Structures of binuclear complexes **A** and **B** and **1a** and **2a**.

A high concentration of reactive oxygen species (ROS) in cancer cells is a well-established phenomenon^58^ that is utilized for activation of aminoferrocene-based antitumor prodrugs.^59,60^ In brief, their mechanism of action includes the initial ROS-activated cleavage of the phenylboronic acid “cap” from the prodrug, which then enables fragmentation of the thus-obtained molecule to form organic quinone methide (QM) and ferrocenium ion products.^59^ Ferrocenium ions themselves or liberated from them Fe^2+/3+^ ions react with endogenous ROS to further elevate oxidative stress (OS) in cancer cells, which finally leads to deleterious effects. Yet another relevant example of redox-activated anticancer-active ferrocenes pertains to ferrocene-(vinyl)Ru(CO)Cl(P^*i*^Pr_3_)_2_ compounds **A** and **B** (Figure 1).^16,61^

These compounds differ from ferrocifenes and aminoferrocene prodrugs as their molecular structure features two nonequivalent metal redox centers. Combined (spectro)electrochemical, EPR, and Mössbauer studies on **B** revealed that it belongs to class II MV systems.^16^ Interestingly, compound **B** showed high anticancer activity in HT-29 colon carcinoma and MCF-7 breast cancer cells *in vitro*.^61^ Its activity exceeded that of **A**, and it was much better in terms of activity than the corresponding mononuclear ferrocenyl and ruthenium complexes used as references in the same study.^61^ Remarkable biological activity of **A** and **B** has stimulated our interest in the development of new mixed-valence ferrocenyl systems as anticancer agents.

**Scheme 1:**
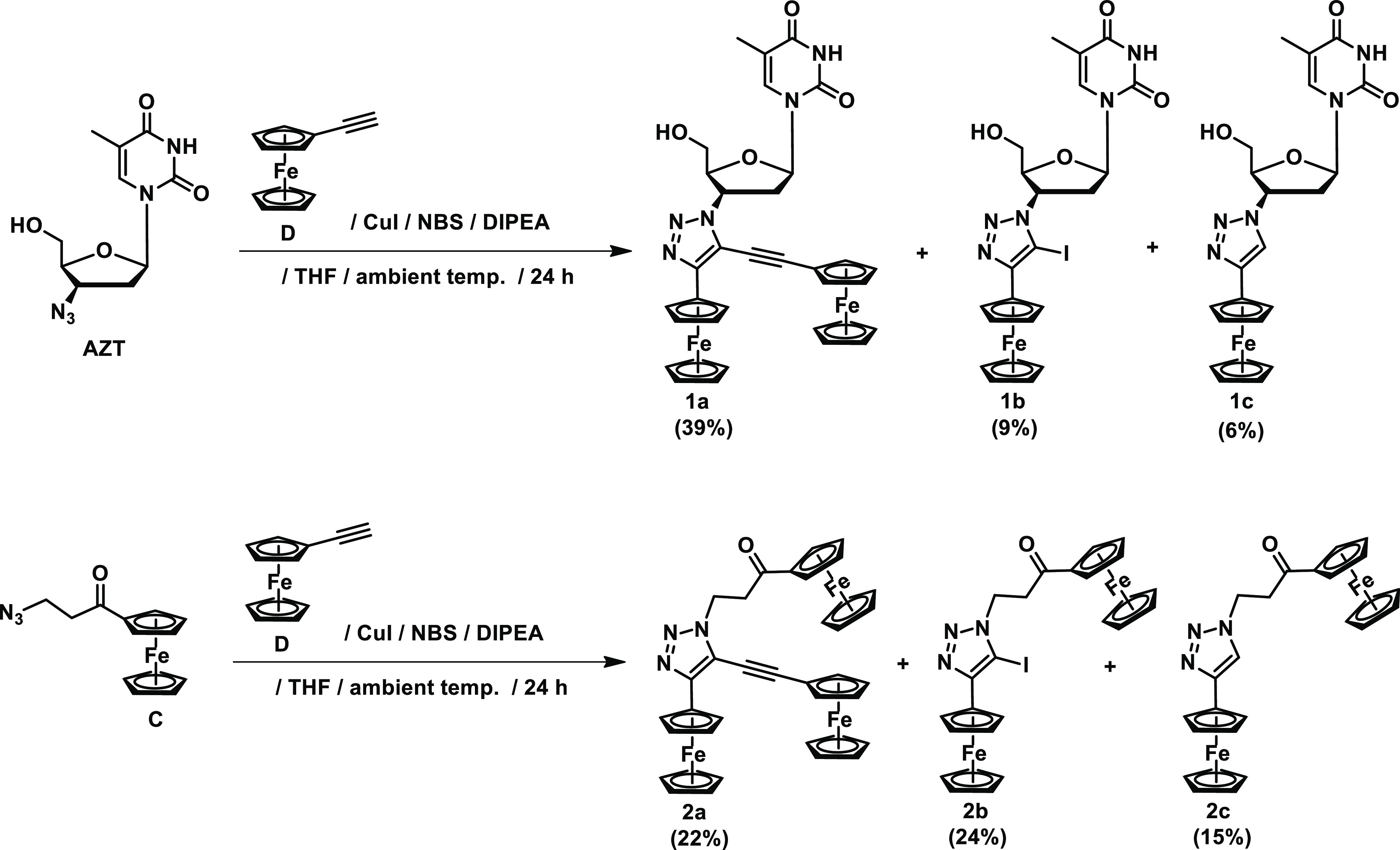
Synthesis of **1a**–**c** and **2a**–**c** AZT = 3′-azido-3′-deoxythymidine; NBS = *N*-bromosuccinimide; DIPEA = *N*,*N*-diisopropylethylamine; THF = tetrahydrofuran.

**Scheme 2:**
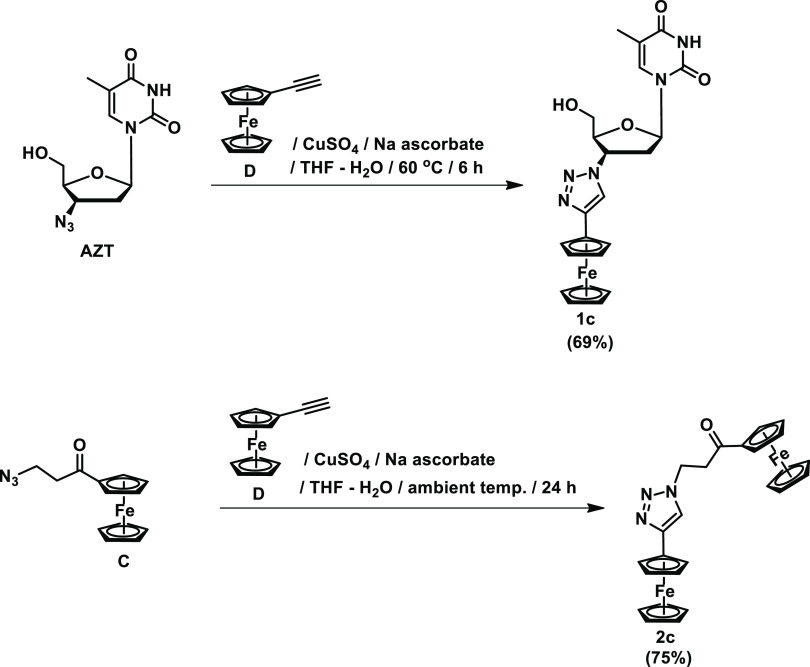
Synthesis of **1c** and **2c** AZT = 3′-azido-3′-deoxythymidine; THF = tetrahydrofuran.

Herein, we report the syntheses and (spectro)electrochemical, EPR, and density functional theory (DFT) studies of 3′-deoxy-3′-(4-ferrocenyl-5-ethynylferrocenyl-1*H*-1,2,3-triazol-1-yl)thymidine (**1a**) and 1-(3-propionylferrocenyl)-4-ferrocenyl-5-ethynylferrocenyl-1*H*-1,2,3-triazole (**2a**) representing bi- and trinuclear ferrocenyl systems, respectively (Figure 1). Furthermore, we report herein on mononuclear congeners of **1a** and **2a** such as 3′-deoxy-3′-(4-ferrocenyl-5-iodo-1*H*-1,2,3-triazol-1-yl)thymidine (**1b**), 1-(3-propionylferrocenyl)-4-ferrocenyl-5-iodo-1*H*-1,2,3-triazole (**2b**), 3′-deoxy-3′-(4-ferrocenyl-1*H*-1,2,3-triazol-1-yl)thymidine (**1c**), and 1-(3-propionylferrocenyl)-4-ferrocenyl-1*H*-1,2,3-triazole (**2c**) (Schemes 1 and 2). The common feature of **1a**–**c** and **2a**–**c** series of compounds is that they contain the 1,2,3-triazole structural motif. Due to the development of the copper-catalyzed 1,3-dipolar azide–alkyne cycloaddition (CuAAC) reaction,^62,63^ the interest in the chemistry of 1,2,3-triazoles has increased greatly in the recent time.^64−68^ In regard to biological applications, 1,2,3-triazoles have proved their value as easy-to-synthesize linkers in bioconjugate chemistry.^30,31,64,68^ In this work, another leap forward has been taken with respect to biological applications of 1,2,3-triazoles as they have been used not only as linkers but also as entities that allow electron transfer between two ferrocenyl groups to occur. The selection of 3′-azido-3′-deoxythymidine (AZT) as the source material for compounds **1a**–**c** was motivated by the biological significance of deoxythymidine nucleoside and general importance of CuAAC reactions in nucleic acid chemistry and biology.^64,68^ Taking into account the above motivation, compounds **1a** and **2a** as well as their mononuclear analogues **1c** and **2c** were used to study their anticancer activity in human A549 and H1975 non-small-cell lung cancer (NSCLC) cells and nonmalignant bronchial epithelium BEAS-2B cells. Anticancer activity assays have been also performed in the presence of free-radical scavenger *N*-acetyl cysteine (NAC) to investigate the impact of ROS on compounds’ activity.

## Results and Discussion

### Synthesis

Compounds **1a** and **2a** belong to 5-alkynyl-1,2,3-triazoles, a subclass of highly substituted 1,2,3-triazole derivatives with great potential for synthetic chemistry. A literature survey shows several synthetic approaches giving an access to this class of compounds.^69−73^ One of them relies on the palladium-catalyzed Sonogashira cross-coupling reaction of 5-iodo-1,2,3-triazoles with terminal alkynes.^65,73^ Due to apparent simplicity, we have chosen this approach for the synthesis of compounds **1a** and **2a**. In the first step, we attempted to obtain the 5-iodo-1,2,3-triazole **1b** and **2b** intermediates. Their syntheses were carried out by the reaction of AZT or 3-azidopropionylferrocene (**C**) with ethynylferrocene (**D**), *N*-bromosuccinimide (NBS), and *N*,*N*-diisopropylethylamine (DIPEA) according to Scheme 1.^74^

As expected, the respective reactions afforded 5-iodo-1,2,3-triazole **1b** and **2b** in 9 and 24% yields, respectively. Besides this and to our satisfaction, reactions also afforded the desired compounds **1a** and **2a** in 39 and 22% yields, respectively. Furthermore, 4-ferrocenyl-1,2,3-triazole derivatives **1c** and **2c** were obtained, although in low yields of 6 and 15%, respectively. We have found that simple modifications of the reaction conditions (*e.g*., increase of either the reaction time and/or temperature) only resulted in a decrease of compounds **1a** and **2a** yield. Also, any attempt to transform **1b** or **2b** into corresponding compounds **1a** and **2a** by the Sonogashira cross-coupling reaction with ethynylferrocene (**D**) failed. On the contrary, the yields of compounds **1c** and **2c** were easily increased using the classical CuAAC reaction conditions according to Scheme 2.

Formation of 5-iodo-1,2,3-triazole **1b** and **2b** can be explained by the mechanism proposed by Zhang.^74^ However, the observation of other reaction products suggests that further mechanism(s) can be also operational in the course of the reaction. Their investigation was out of our interest as the effort was entirely focused on electronic coupling and anticancer activity studies. After completion of the reaction and purification, compounds **1a** and **2a**–**c** were isolated as orange crystalline solids, whereas **1b** and **1c** were isolated as yellow crystalline solids. Characterization of all complexes was carried out with ^1^H and ^13^C NMR and IR spectroscopy, mass spectrometry, and elemental analyses. The ^1^H and ^31^C NMR spectra of **1a**–**c** and **2a**–**c** are shown in Figures S1–S12 (see the SI). Furthermore, the structures of **1a**, **2a**, and **2c** in the solid state were determined by single-crystal X-ray structural analysis.

### Crystallographic Studies

**Figure 2:**
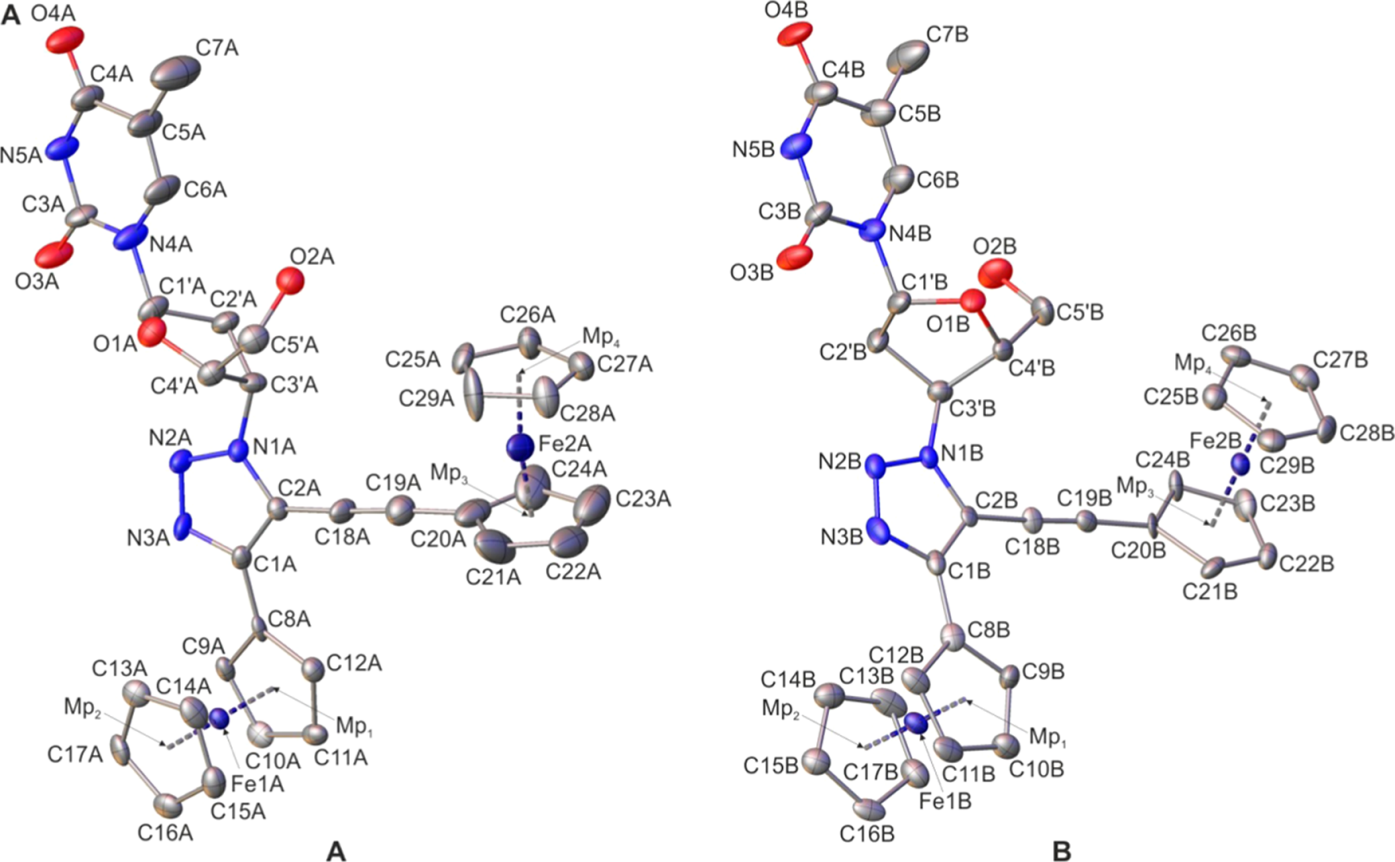
Molecular structure of **1a** (two crystallographically independent molecules in the crystal, **A** and **B**) with atomic displacement ellipsoids at the 50% probability level. The H-atoms are omitted for clarity. Mp_1_, Mp_2_, Mp_3_, and Mp_4_ pertain to the mid-points of the cyclopentadienyl rings. Selected bond lengths, distances [Å], and angles [deg] for molecule **A**/molecule **B**: Mp_1_–Mp_2_, 3.298(5)/3.308(5); Mp_3_–Mp_4_, 3.279(5)/3.311(5); Fe1A/Fe1B···Fe2A/Fe2B, 10.981(13)/11.055(11) (sum of the bond lengths); Fe1A–C8A/Fe1B–C8B, 2.061(8)/2.084; Fe2A–C20A/Fe2B–C20B, 1.999(8)/2.083(7); C1A–C8A/C1B–C8B, 1.456(12)/1.485(13); C18A–C19A/C18B–C19B, 1.214(15)/1.175(12); C2A–N1A/C2B–N1B, 1.351(11)/1.360(10); N1A–N2A/N1B–N2B, 1.331(11)/1.335(10); N2A–N3A/N2B–N3B, 1.321(10)/1.301(11); N3A–C1A/N3B–C1B 1.362(11)/1.370(11); C1A–C2A/C1B–C2B, 1.382(12)/1.382(12); C2A–C18A–C19A/C2B–C18B–C19B, 175.9(1)/175.6(8); C18A–C19A–C20A/C18B–C19B–C20B, 173.4(11)/176.6(9); C8A–C1A–C2A–C18A/C8B–C1B–C2B–C18B, 0.5(16)/–5.8(15); C1′A–O1A–C4′A–C3′A/C1′B–O1B–C4′B–C3′B, −4.5(10)/–6.2(9).

**Figure 3:**
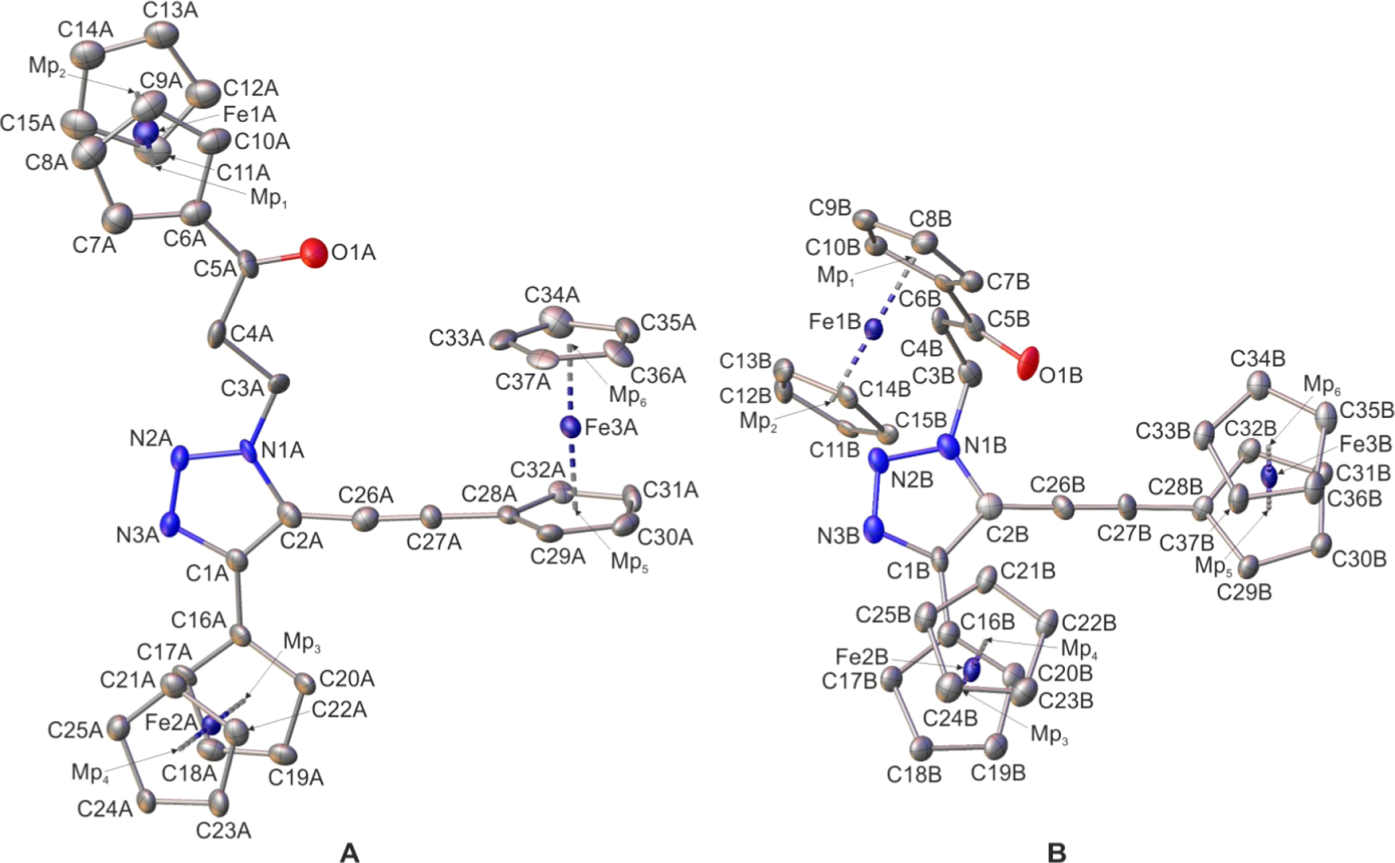
Molecular structure of **2a** (two crystallographically independent molecules in the crystal, **A** and **B**) with atomic displacement ellipsoids at the 50% probability level. The H-atoms are omitted for clarity. Mp_1_, Mp_2_, Mp_3_, Mp_4_, Mp_5_, and Mp_6_, pertain to the mid-points of the cyclopentadienyl rings. Selected bond lengths, distances [Å], and angles [deg] for molecule **A**/molecule **B**: Mp_1_–Mp_2_, 3.316(9)/3.306(7); Mp_3_–Mp_4_, 3.302(8)/3.294(8); Mp_5_–Mp_6_, 3.289(8)/3.296(8); Fe2A/Fe2B···Fe3A/Fe3B, 8.548(3)/6.770(3) (through space distance) and 10.920(16)/10.934(16) (sum of the bond lengths); Fe1A/Fe1B···Fe2A/Fe2B, 10.981(13)/11.055(11) (sum of the bond lengths); Fe1A–C6A/Fe1B–C6B, 2.030(14)/2.047(12); Fe2A–C16A/Fe2B–C16B, 2.033(12)/2.045(13); Fe3A–C28A/Fe3B–C28B, 2.058(12)/2.047(12); C1A–C2A/C1B–C2B, 1.378(18)/1.397(18); C1A–N3A/C1B–N3B, 1.381(16)/1.356(16); C1A–C16A/C1B–C16B, 1.435(17)/1.441(18); C2A–N1A/C2B–N1B, 1.405(17)/1.358(17); N1A–N2A/N1B–N2B, 1.296(14)/1.343(15); N2A–N3A/N2B–N3B, 1.327(15)/1.331(15); C26A–C27A/C26B–C27B, 1.211(19)/1.209(17); C2A–C26A–C27A/C2B–C26B–C27B, 174.6(15)/178.0(14); C26A–C27A–C28A/C26B–C27B–C28B, 177.2(14)/178.8(15); C16A–C1A–C2A–C26A/C16B–C1B–C2B–C26B, 9(3)/4(2); N1A–C3A–C4A–C5A/N1B–C3B–C4B–C5B, −179.3(10)/70.4(13).

**Figure 4:**
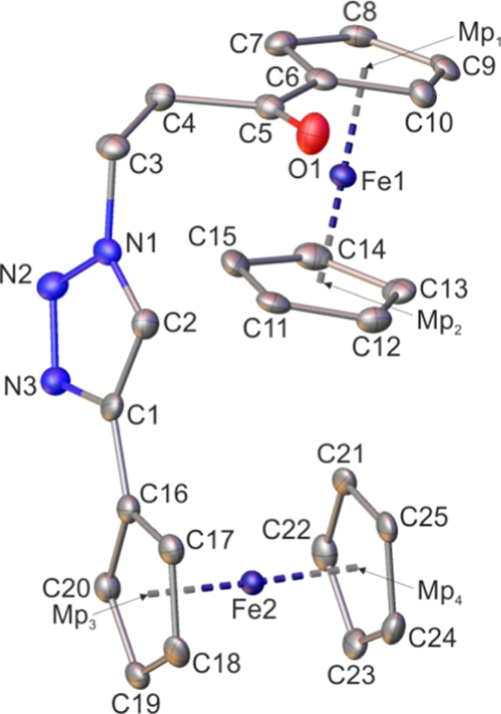
Molecular structure of **2c** with atomic displacement ellipsoids at the 50% probability level. The H-atoms are omitted for clarity. Mp_1_, Mp_2_, Mp_3_, and Mp_4_ pertain to the mid-points of the cyclopentadienyl rings. Selected bond lengths, distances [Å], and angles [deg]: Mp_1_–Mp_2_, 3.306(3); Mp_3_–Mp_4_, 3.298(3); Fe1–C6, 2.036(5); Fe2–C16, 2.049(6); C1–C2, 1.379(8); C5–C6, 1.473(8); N1–C2, 1.350(7); N3–C1, 1.363(7); N2–N1, 1.340(7); N3–N2, 1.318(7); O1–C5, 1.219(7); C22–C21–C25–C24, 0.2(7); C1–C2–N1–C3, 176.2(5); C1–C2–N1–N2, 0.5(6); C3–C4–C5–C6, 167.9(4); C4–C3–N1–N2, −64.2(7).

Single-crystals of **1a**, **2a**, and **2c** suitable for X-ray diffraction (XRD) analysis were obtained by diffusion of *n*-hexane in a solution of the respective complex in dichloromethane at room temperature. The crystal and structure refinement data are presented in Table S1 (see the SI). The molecular structures of **1a**, **2a**, and **2c** with the atom-labeling scheme and selected geometrical parameters are provided in Figures 2–4, respectively. The bond distances (Å) and valence and torsion angles (deg) are given in Tables S2–S10 (see the SI). Compounds **1a** and **2c** both crystallized in the orthorhombic space group, *P*2_1_ (**1a**) and *Cc* (**2c**). Compound **2a** crystallized in the monoclinic space group **P**2_1_/**c**. In the crystals of **1a** and **2a**, two crystallographically independent molecules (**A** and **B**) are observed.

Crystallographic analysis confirmed the postulated structures of examined complexes and indicate their conformational flexibility (two different conformers for **1a** and **2a** in the crystal lattices). Particularly, for **1a** and **2a**, the molecular architecture in which the ferrocenyl and the ethynylferrocenyl entities are bonded to a 1,2,3-triazole scaffold in a 4,5-substitution pattern was unambiguously confirmed. The through space distance between the Fe atoms in **1a** was 8.402(2) and 8.075(2) Å in conformers **A** and **B**, respectively. The analogous distance for compound **2a** was 8.548(3) Å (conformer **A**) and 6.770(3) Å (conformer **B**). The sandwich Fc groups adopt intermediate conformations between the staggered and the eclipsed form.^75^Table S11 (see the SI) provides the geometrical details for these conformations. The geometry of the thymine nucleobase in **1a** does not show significant differences with similar species reported in the literature.^76^ Furthermore, structural analysis confirmed that the absolute configuration of the deoxyribosyl moiety present in two independent molecules of **1a** in the crystal can be assigned as D (d-ribose). Of notice is, however, that the sugar conformations are different in each independent molecule. The puckering of the deoxyribosyl moiety within conformer **A** adopts an envelope C2′-endo conformation, whereas in conformer **B**, a twist C2′-endo–C3′-exo conformation is characteristic.^77,78^ The numerical data for both conformations are given in Table S12 (see the SI).

### (Spectro)electrochemistry

**Figure 5:**
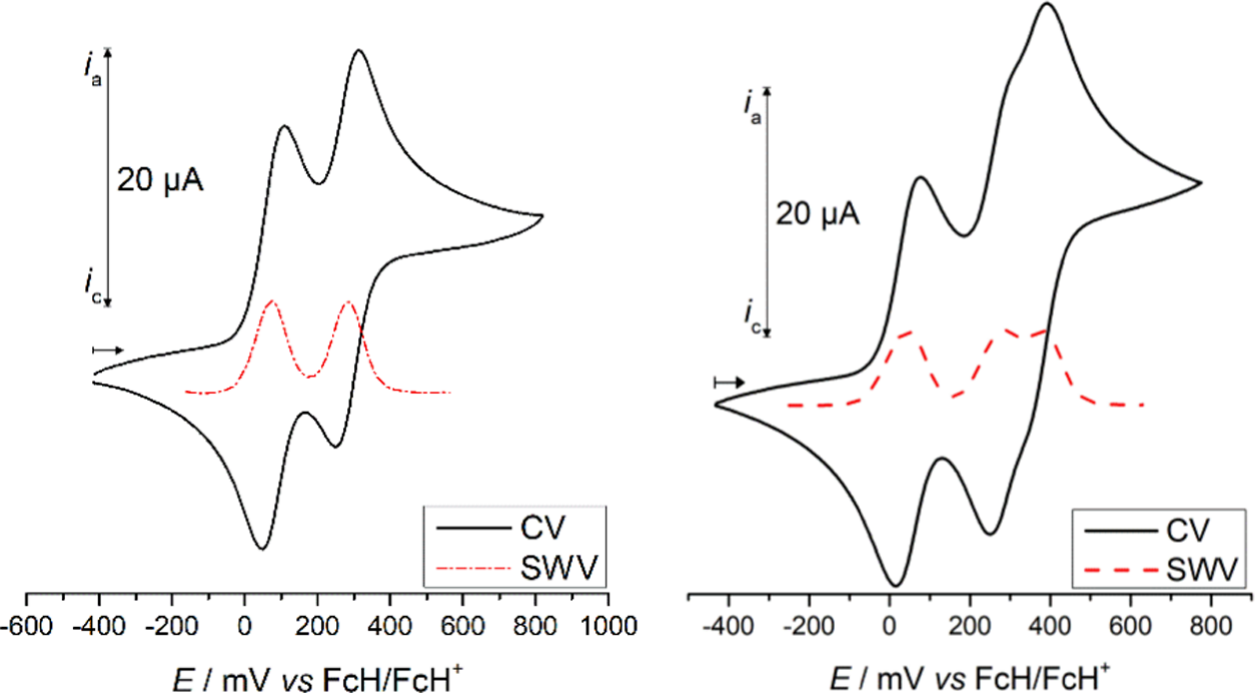
Cyclic voltammograms of **1a** (left) and **2a** (right) (potential area −500 to 800 mV) as well as square-wave voltammograms (dotted lines) (potential area −200 to 600 mV). Measurement conditions: scan rates, 100 mV·s^–1^ (CV) and 5 mV·s^–1^ (SWV) in anhydrous dichloromethane solutions (1.0 mmol·L^–1^); supporting electrolyte, 0.1 mol·L^–1^ of [NBu_4_][B(C_6_F_5_)_4_]; working electrode, glassy carbon.

Electrochemical studies of compounds **1a**, **1c**, **2a**, and **2c** were carried out using cyclic voltammetry (CV) and square-wave voltammetry (SWV) (Table 1; Figures 5 (compounds **1a**, **2a**) and S13 (compounds **1c**, **2c**), see the SI). A solution of [NBu_4_][B(C_6_F_5_)_4_] (0.1 mol·L^–1^) in anhydrous CH_2_Cl_2_ was used as the supporting electrolyte.^79^ The choice of the supporting electrolyte was motivated by the beneficial properties of [B(C_6_F_5_)_4_]^−^ ions. In contrast to smaller counter ions such as [Cl]^−^, [PF_6_]^−^, [BF_4_]^−^, or [ClO_4_]^−^, [B(C_6_F_5_)_4_]^−^ tolerates the stabilization of greatly charged species in solution, minimizing undesired ion-pairing effects.^80,81^ The voltammetry experiments were performed at 25 °C. All potentials are referenced to the FcH/[FcH]^+^ (Fc = Fe(η^5^-C_5_H_4_)(η^5^-C_5_H_5_)) redox couple (*E*°′ = 0 mV).^82^

**Table 1:** Cyclic Voltammetry Data of **1a**, **1c**, **2a**, and **2c**^a^

compound	*E*_1_°′/mV^b^ (Δ*E*_p_/mV^c^)	*E*_2_°′/mV^b^ (Δ*E*_p_/mV^c^)	*E*_3_°′/mV^b^ (Δ*E*_p_/mV^c^)	*K*_C_^d^
**1a**	80 (60)	280 (66)		2412
**1c**	60 (66)			
**2a**	45 (60)	280 (61)	365 (63)	9426
**2c**	20 (61)		330 (67)	

a Potentials *vs* [FcH]/[FcH]^+^ (scan rate 100 mV·s^–1^) at a glassy carbon electrode of 1.0 mmol·L^–1^ solutions of the analyte in anhydrous dichloromethane containing 0.1 mol·L^–1^ [NBu_4_][B(C_6_F_5_)_4_] as the supporting electrolyte at 25 °C.

b *E*°′ = formal potential.

c Δ*E*_p_ = difference between the cathodic and anodic peak potentials |*E*_pc_ – *E*_pa_|.

d *K*_C_ = comproportionation constant *K*_C_ = exp(*nF*/*RT*)Δ*E*_1/2_, *F* = Faraday constant, *R* = gas constant, *T* = temperature, Δ*E*_1/2_ = difference of half-wave potentials, *n* = number of transferred electrons.

The cyclic voltammogram of **1a** shows two separated reversible redox events at 80 and 280 mV, while **2a** with its further FcC(O)CH_2_CH_2_ unit features in total three redox processes at 45, 280, and 365 mV *vs* FcH/[FcH]^+^, as expected (Figure 5 and Table 1). To assign the appropriate redox waves, compounds **1c** and **2c** were measured under identical conditions. It was found that the ferrocenyl-based redox event of **1c** appears at 60 mV and the ones of **2c** appear at 20 and 330 mV (Table 1 and Figure S13, see the SI). Comparing these values leads to the conclusion that the first oxidation occurs at the Fc moiety directly bonded to the 1,2,3-triazole core. Such an assignment is consistent with data obtained for other ferrocenyl-1,2,3-triazole systems^83−85^ and supported by DFT calculations (see the SI). In the following electrochemical process, the respective FcC≡C unit is oxidized. The potentials confirm that compound **2a** is more electron-rich than **1a** and hence is easier to be oxidized, whereas the follow-up redox event occurs at the same potential. The difference between the formal potentials is 200 mV for **1a** and 235 mV for **2a** (Table 1), pointing to the fact that monocationic [**2a**]^+^ should be a somewhat more stable mixed-valent species than [**1a**]^+^ (*vide supra*). The formal potential of the FcC(O)CH_2_CH_2_ terminal group can be found at 330 (**2c**) and 365 mV (**2a**) due to the influence of the previously introduced positive charges.

**Figure 6:**
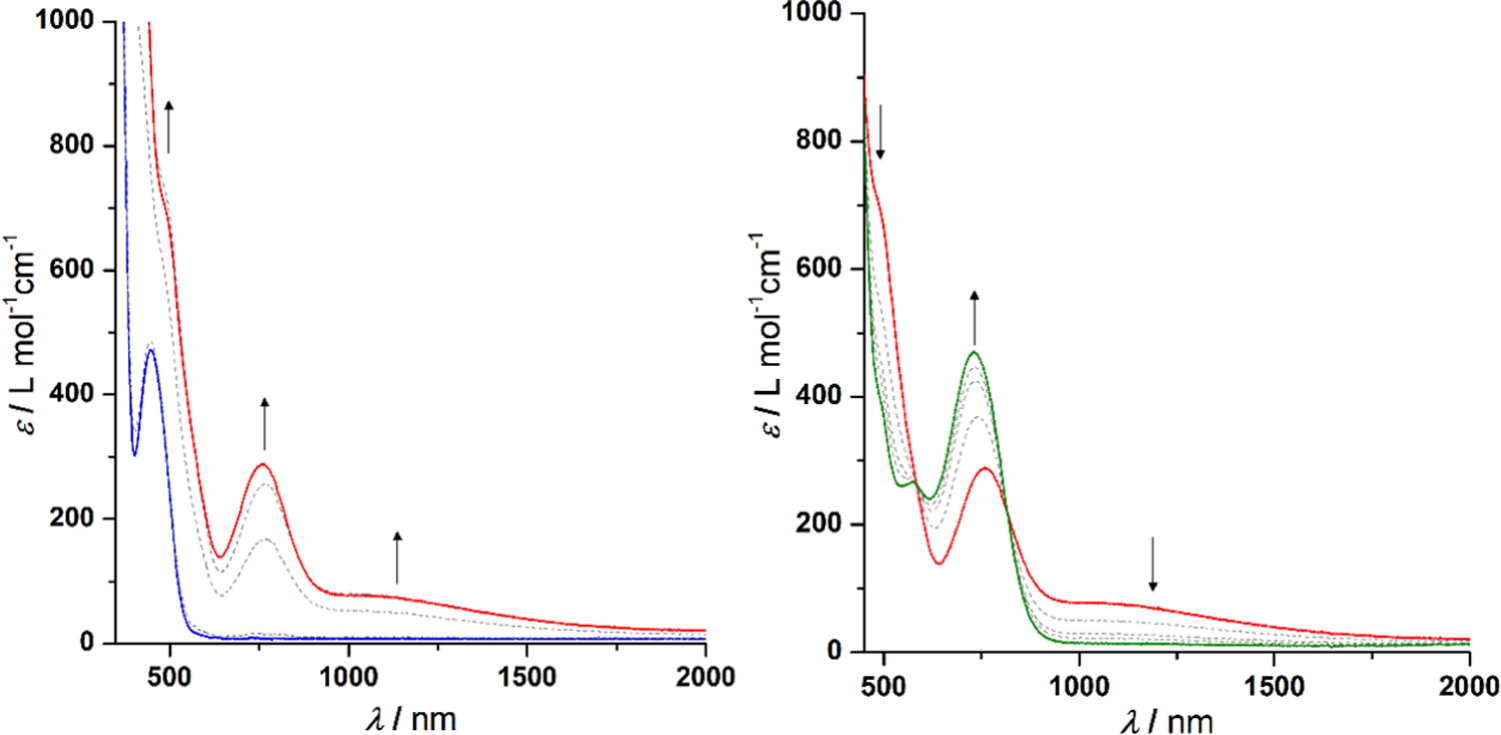
UV–vis/NIR spectra of **1a** at 0–250 mV (left) and 250–500 mV (right) *vs* Ag/AgCl in an OTTLE cell; measurement conditions: 25 °C, 5.0 mmol·L^–1^ analyte solution in tetrahydrofuran, and 0.1 mol·L^–1^ [N^*n*^Bu_4_][B(C_6_F_5_)_4_]; arrows indicate absorption changes.

**Figure 7:**
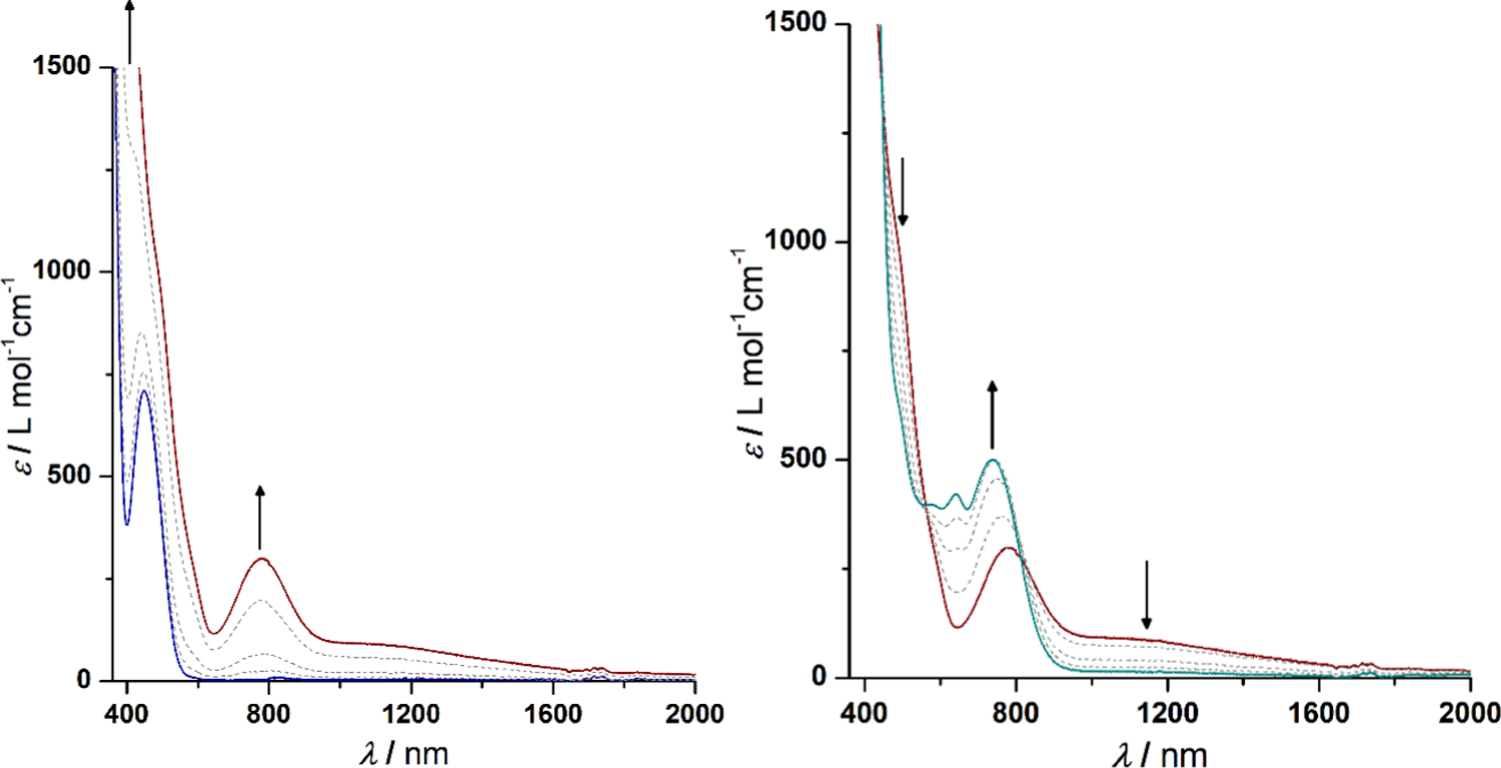
UV–vis/NIR spectra of **2a** at 150–275 mV (left) and 275–800 mV (right) *vs* Ag/AgCl in an OTTLE cell; measurement conditions: 25 °C, 5.0 mmol·L^–1^ analyte solution in tetrahydrofuran, and 0.1 mol·L^–1^ [N^*n*^Bu_4_][B(C_6_F_5_)_4_]; arrows indicate absorption changes.

The *in situ* electrochemical behavior of **1a** (Figure 6) and **2a** (Figure 7) was investigated by spectroelectrochemical UV–vis/NIR measurements within an optically transparent thin-layer electrochemical (OTTLE^86^) cell with SiO_2_ windows in tetrahydrofuran solutions of the analyte, containing [NBu_4_][B(C_6_F_5_)_4_] (0.1 mol·L^–1^) as the supporting electrolyte.^87,88^ In the course of the measurements, the applied cell potential was increased stepwise (step width: 25, 50, or 100 mV). At the end of each measurement, the analyte was reduced at −500 mV *vs* Ag/AgCl for 30 min, and an additional spectrum was recorded to prove the reversibility of the oxidation. The spectroelectrochemical UV–vis/NIR data of **1a** in tetrahydrofuran display weak absorptions in the NIR region between 0 and 250 mV *vs* Ag/AgCl upon formation of the mixed-valent species [**1a**]^+^ (Figure 6). A further increase of the potential leads to the generation of dicationic [**1a**]^2+^ (250–500 mV *vs* Ag/AgCl). The measurements confirm that [**1a**]^+^ exhibits IVCT absorption of a weak strength, indicating reduced coupling between the Fc and the [Fc]^+^ entity. Similar observations were made for the UV–vis/NIR spectra of **2a** (Figure 7). Further analysis of both IVCT absorptions via deconvolution of the resulting bands confirmed that the weak nature of these transitions is less pronounced for **2a** (ṽ_IVCT_ = 9255 cm^–1^, ε_max_ = 80 L·mol^–1^·cm^–1^, Δṽ_1/2_ = 6215 cm^–1^) than **1a** (ṽ_IVCT_ = 9040 cm^–1^, ε_max_ = 65 L·mol^–1^·cm^–1^, Δṽ_1/2_ = 4795 cm^–1^) (Figure S14, see the SI). Based on these values,^89^ the electronic matrix coupling element *V*_ab_ (*H*_ab_) (eq 1S, see the SI) can be calculated and results in 100 cm^–1^ for **1a** and 127 cm^–1^ for **2a**, confirming the weak nature of their electronic coupling.

**Figure 8:**
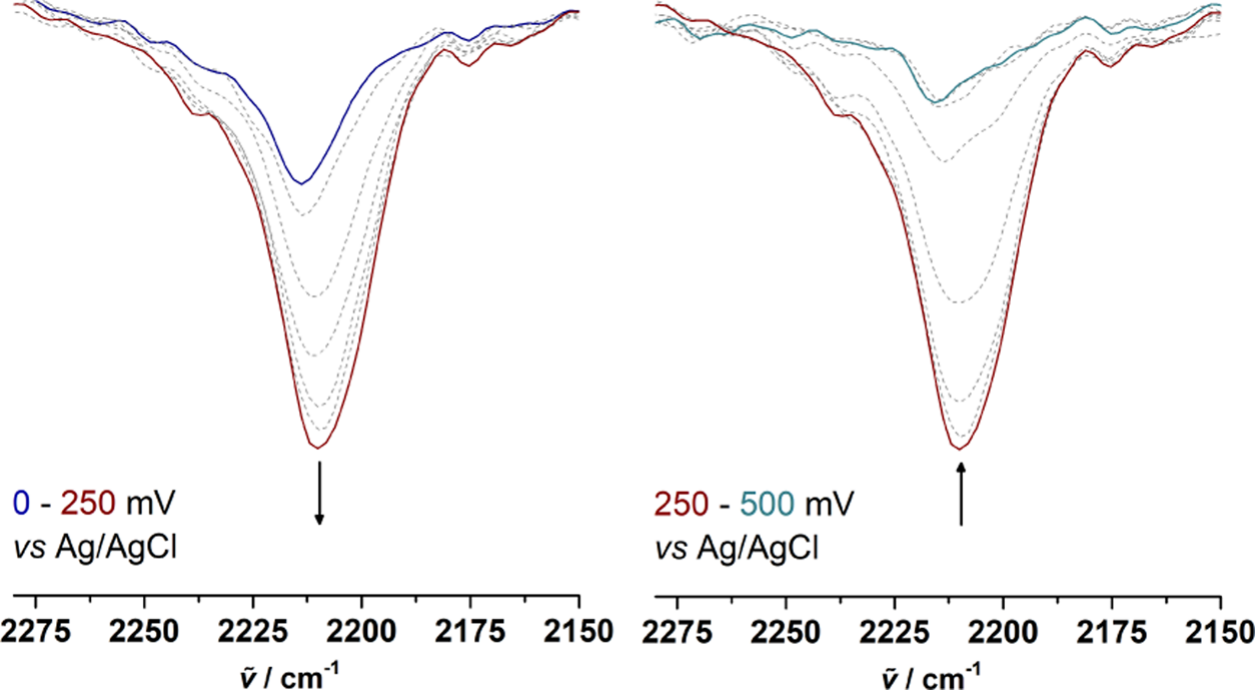
IR spectra (2150–2300 cm^–1^) of **1a** at 0–250 mV (left) and 250–500 mV (right) *vs* Ag/AgCl in an OTTLE cell; measurement conditions: 25 °C, 5.0 mmol·L^–1^ analyte solution in tetrahydrofuran, 0.1 mol·L^–1^ [N^*n*^Bu_4_][B(C_6_F_5_)_4_], arrows indicate increasing or decreasing ν_C≡C_ vibrations.

In the example of **1a**, spectroelectro-IR studies were carried out applying an OTTLE cell with CaF_2_ windows under identical measurement conditions (*vide infra*). Oxidation of neutral **1a** to monocationic [**1a**]^+^ leads to higher intensities of the triple bond vibrational band, which is accompanied by a shift from 2214 to 2210 cm^–1^ (Figure 8). Smaller wavenumbers imply that the carbon–carbon triple bond comprises more electron density in [**1a**]^+^, proposing that electron transfer between the ferrocenic species passes through the carbon–carbon triple bond, making this a “through-bond” electron transfer process. A further increase of potential leads to the generation of [**1a**]^2+^, which is followed by a characteristic shift of the band from 2210 to 2216 cm^–1^. This observation is the result of decreased electron density due to both ferrocenyl systems featuring Fe^3+^ ions. Therefore, electron delocalization between the Fc and FcC≡C units *via* the 1,2,3-triazole connectivity is reduced compared to [**1a**]^+^.

A bathochromic (4 cm^–1^) and hypsochromic (6 cm^–1^) shifts in the infrared C≡C stretching vibration, observed during the first (**1a** → **1a**^+^) and the second (**1a**^**+**^ → **1a**^**2**+^) oxidation, respectively, were reproduced at the BLYP/6-31+G(d)/LanL2DZ level of theory (see the DFT Calculations section and the SI for details).

### DFT Calculations

**Figure 9:**
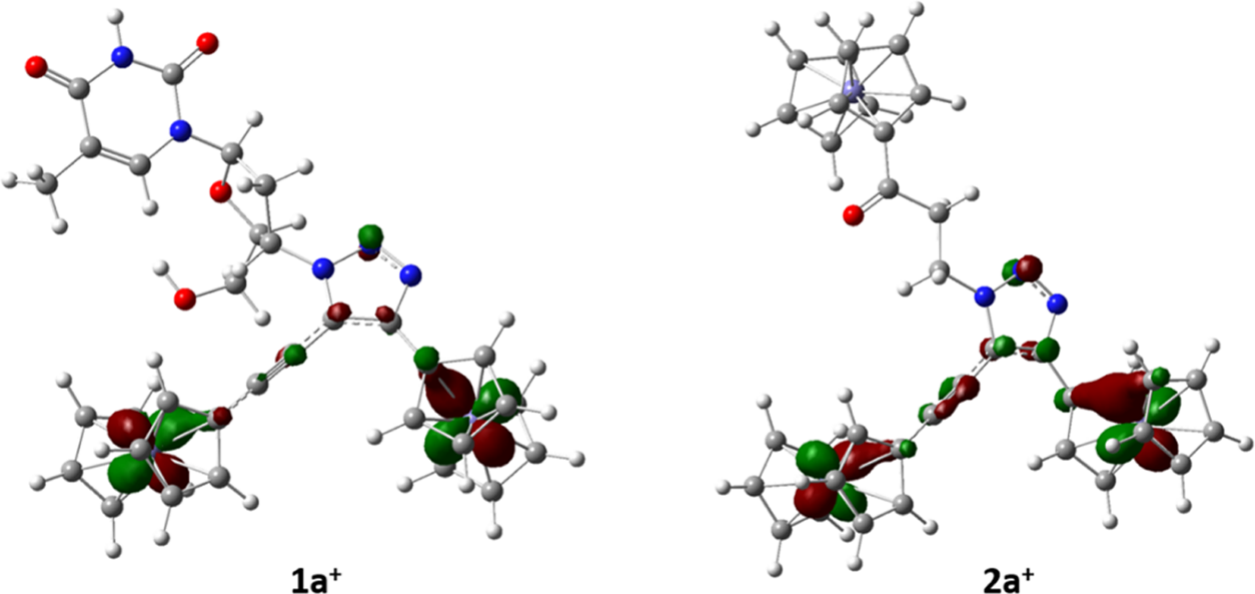
SOMO orbitals in open-shell species [**1a**]^+^ and [**2a**]^+^ calculated at the BLYP/6-31+G(d)/LanL2DZ level of theory. Atomic radii scaled by 50%.

To gain more detailed insight into the electronic structures of the examined compounds, calculations were carried out at the BLYP/6-31+G(d)/LanL2DZ level of DFT theory^90^ utilizing the Gaussian 16 code.^91^ Details on structural optimization and calculations are provided in the Experimental Section and the SI. According to DFT calculations, the highest occupied molecular orbital (HOMO) orbital of **1a**, **1c**, **2a**, and **2c** is localized at the ferrocenyl group directly bonded to the 1,2,3-triazolyl moiety (Figure S15). Upon first oxidation, one electron (a β spin state) is removed from the 3d*_xy_* orbital of the ferrocene ring. The 3d*_xy_* orbital becomes the singly occupied molecular orbital (SOMO) for the α-electron and the lowest unoccupied molecular orbital (LUMO) in the β-electron configuration in the oxidized species. In the case of [**1a**]^+^ and [**2a**]^+^, the spin density is not located on one ferrocenyl group but expands over the *ca.* 11 Å ferrocenyl-1,2,3-triazolyl-ethynylferrocenyl part of the molecule (Figure 9). This feature provides additional evidence for the possibility of electron communication between the two Fc moieties in [**1a**]^+^ and [**2a**]^+^. However, the spin density is not uniformly distributed over the 1,2,3-triazolyl bridge. Its highest contribution is on the two carbon (formally C=C bond) and the middle nitrogen atom of the 1,2,3-triazolyl core.

DFT calculations were found very useful with respect to spectroelectro-IR study result interpretation. Accordingly, an excellent agreement between experimental and calculated C≡C bond stretching frequencies was obtained (Table S13, see the SI). This further validates our theoretical approach and supports the experimental evidence of the electron transfer between the two ferrocenyl moieties in [**1a**]^+^. In the dicationic species [**1a**]^2+^, however, the “through-bond” electron transfer was lost, as both ferrocenyl units exist in the Fe^3+^ form. According to calculations, the ground state of [**1a**]^2+^ was found to be a triplet state (rather than a single state) with the two singly occupied MOs (Figure S16, see the SI). Interestingly, the relative increase in the C≡C stretching frequency ([**1a**]^+^ < **1a** < [**1a**]^2+^) correlates well with the calculated C≡C bond length in the respective series (Table S13, see the SI): with an increase in frequency, the bond becomes shorter. The relative change is small but indicative. This also supports the involvement of the C≡C bond in Fe^2+^–Fe^3+^ delocalization in [**1a**]^+^ on the intrinsic IR time scale and the lack of the corresponding communication between the two ferrocenyl entities in [**1a**]^2+^.

### Electron Paramagnetic Resonance (EPR) Spectroscopic Study

With the purpose of gaining better insights into the charge delocalization in one-electron oxidized compounds, we performed *in situ* EPR spectroelectrochemical measurements for compounds **1a** and **2a**. While the organic radical could be obtained at room temperature, an anisotropic signal of the ferrocenium ion is only detectable at low temperature (below 77 K) due to fast spin-lattice relaxation. The EPR spectra of electrochemically generated [**1a**]^+^ and [**2a**]^+^ show no signals at 298 and 85 K. The absence of any signals during the first redox event under specified conditions indicates that the oxidation process in the compounds is predominantly located on the ferrocenyl moiety at the EPR time scale, substantiating the presence of the weakly coupled class II MV system according to Robin and Day. Thus, further information about the electronic coupling between the ferrocenyl groups in [**1a**]^+^ and [**2a**]^+^ cannot be provided with EPR due to experimental limitations.

**Figure 10:**
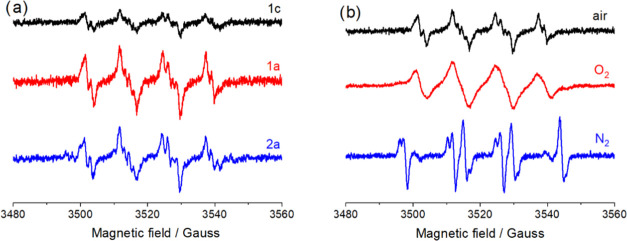
EPR spectra measured in DMF solutions containing (a) **1c**, **1a**, and **2a** under air conditions and (b) **1a** under different conditions (air, O_2_, N_2_), *T* = 295 K.

Instead, the EPR spin-trapping technique was employed to detect short-lived free radicals (reactive oxygen species; ROS) generated in dimethylformamide (DMF) solutions of ferrocene compounds in the presence of molecular oxygen. Free radicals are key cell-damage causative agents that are often generated by ferrocenium species inside cancer cells.^27,31,59−61^ It was therefore justified to check whether our compounds are also capable of free-radical generation. In this regard, 5,5-dimethyl-1-pyrroline *N*-oxide (DMPO) was used as a spin trap. The EPR spectra measured in air-saturated DMF solutions selected for measurement compounds of **1a**, **1c**, and **2a** show a mixture of DMPO adducts, indicating the production of several free radicals (Figure 10).

On the basis of the hyperfine splitting constants of DMPO adducts,^92^ the main radicals formed in the systems are oxygen-centered ones (superoxide radical anion O_2_^•–^ and its protonated form hydroperoxyl radical ^•^OOH). The simulated spectra fit very well with the experimental ones (Figure S17, see the SI). EPR parameters of the spin trap adducts obtained from simulations of experimental spectra are presented in Table 2.

**Table 2:** EPR Parameters of DMPO Spin Adducts^a^

	hyperfine splitting constants (G)				
experimental conditions	*a*(^14^N)	*a*(^1^H_β_)	*a*(^1^Hγ)	*g* value	radical
**1a**					
air	**12.84**	**10.15**	**1.39**	**2.00596**	**O**_**2**_^**•–**^
	**13.81**	**11.71**	**0.83**	**2.00579**	^**•**^**OOH**
O_2_	**13.10**	**10.63**		**2.00590**	**O**_**2**_^**•–**^
	14.38	16.47		2.00585	^•^CH_2_N(CH_3_)CHO
N_2_	**14.36**	**17.66**		**2.00572**	^**•**^**CH**_**2**_**N(CH**_**3**_**)CHO**
	**14.27**	**19.94**		**2.00579**	^**•**^**CH**_**3**_
	13.37	11.53	0.97	2.00583	^•^OOH
**2a**					
air	**12.93**	**10.21**	**1.38**	**2.00588**	**O**_**2**_^**•–**^
	**13.93**	**11.96**	**0.94**	**2.00571**	^**•**^**OOH**
	14.21	16.93		2.00583	^•^CH_2_N(CH_3_)CHO
	14.07	20.81		2.00578	^•^CH_3_

a Main adducts are shown in bold.

Under O_2_-saturated conditions, the signal of the superoxide radical anion adduct of DMPO is significantly broadened due to the high concentration of radicals in the solution (Figures 10b and S18a). All of these observations are the confirmation of a single-electron-transfer reaction between a ferrocenyl group and molecular oxygen, resulting in the formation of superoxide anion radicals. It should be also noted that the concentration of the radicals formed in the system with **1c** is much lower than that with **1a** and **2a**. It indicates that the binuclear compounds containing ferrocenyl and ethynylferrocenyl moieties are more effective ROS generators. In an inert (N_2_) atmosphere, carbon-centered (alkyl) radicals are mainly formed (Figures 10b and S18b, see the SI). Radicals ^•^CH_3_ and ^•^CH_2_N(CH_3_)CHO have been earlier found as a result of ultrasound-induced pyrolysis of DMF.^93^ The main DMPO adducts obtained under an inert atmosphere can be assigned to DMPO/^•^CH_3_ and DMPO/^•^CH_2_N(CH_3_)CHO. The alkyl radicals of DMF are also present in small amounts in air- and O_2_-saturated solutions.

### Antiproliferative Activity

Our first reports on anticancer-active MV ferrocenyl compounds occurred over a decade ago.^16,61^ Recently, they were followed by another report on anticancer-active electronically coupled ferrocene systems.^94^ Herein, the antiproliferative activity of **1a**, **1c**, **2a**, and **2c** is examined in human NSCLC A549 and H1975 cells as well as against nonmalignant human bronchial epithelium BEAS-2B cells. The calculated IC_50_ concentrations after 72 h of compound incubation with the cells are shown in Table 3 (cell survival curves related to IC_50_ values are provided in Figures S19–S27).

**Table 3:** Antiproliferative Activity (IC_50_; μM) of Compounds **1a**, **1c**, **2a**, **2c**, and Reference Drugs (Cisplatin, Tamoxifen, and 5-Fluorouracil) against Human NSCLC A549 and H1975 Cells and Nonmalignant Human Bronchial Epithelium BEAS-2B Cells^a^

compound	A549	SInd	H1975	SInd	BEAS-2B
**1a**	57 ± 18	8.2	5 ± 2	93.8	469 ± 10
**1c**	230 ± 13	0.9	456 ± 17	0.5	215 ± 7
**2a**	184 ± 7	1.4	84 ± 5	3.0	257 ± 5
**2c**	805 ± 72	0.2	122 ± 45	1.6	198 ± 7
cisplatin	108 ± 12	0.02	4 ± 0.1	0.7	3 ± 0.1
tamoxifen	72 ± 9	0.1	37 ± 5	0.2	9 ± 0.2
5-fluorouracil	69 ± 21	0.1	32 ± 12	0.2	6 ± 0.1

a IC_50_ was defined as the compound concentration causing a 50% decrease in cell viability in compared to the viability of untreated cells. The selectivity index (SInd) was calculated from the simple equation: IC_50_(BEAS-2B)/IC_50_(A549 or H1975). Treatment time, 72 h.

**Figure 11:**
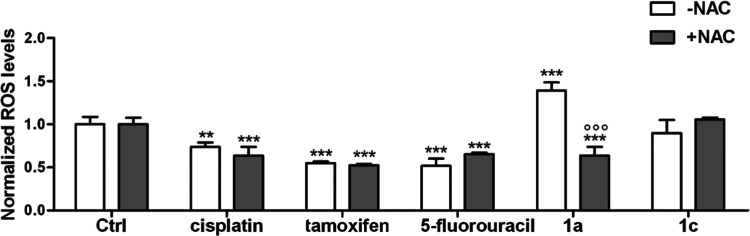
Relative ROS amount in H1975 cells treated with 20 μM of compounds **1a** and **1c** and reference drugs with or without 50 μM NAC. The ROS levels were measured by a fluorimetric assay in duplicates. Data are mean ± standard deviation (SD) (*n* = 3). **p < 0.01, ***p < 0.001: compound-treated cells *vs* respective untreated (Ctrl) cells; ^○○○^*p* <0.001: compound-treated cells *vs* compound + NAC-treated cells.

The most active complexes among ferrocenyl compounds tested were **1a** and **2a**. Noticeably, compound **1a** was more active against H1975 cells than tamoxifen and 5-fluorouracil and almost equally active as cisplatin (5 ± 2 (**1a**) *vs* 4 ± 0.1 μM(cisPt)). Furthermore, it was found that **1a** was more active against A549 in comparison to all three reference compounds tested. An important feature of binuclear compound **1a** is that it shows a remarkably high selectivity index (SInd) toward H1975 (93.8) and A549 (8.2) cells. Higher selectivity toward cancer cells over nonmalignant BEAS-2B cells was also observed for compound **2a**, which might be indicative of similar mechanisms for **1a** and **2a** but not for their mononuclear congeners **1c** and **2c**, respectively. Of remark is that the SInd for all reference drugs tested was low and ranged from 0.02 (A549 for cisplatin) to 0.7 (H1975 for cisplatin), indicating high undesirable toxicity toward nonmalignant cells. In other words, the most anticancer-active compound, **1a** had an IC_50_ value of 469 ± 10 μM against BEAS-2B cells, respectively, which is about 156-, 52-, and 78-times higher values than the IC_50_ values for cisplatin, tamoxifen, and 5-fluorouracil (3 ± 0.1, 9 ± 0.2, and 6 ± 0.1 μM), respectively, against the same BEAS-2B cells. Antiproliferative activity assays showed that cancer cells rich in ROS^58,95^ are more susceptible to **1a** and **2a** in comparison to normal BEAS-2B cells. Likewise, mononuclear compounds **1c** and **2c** showed negligible activity in either cancer or noncancerous cells. For anticancer activity, the presence of two electronically connected ferrocenyl groups is required. However, of **1a** and **2a** compounds, the latter had one ferrocenyl entity more than the former but it shows a lower anticancer effect. This observation indicates that also the nucleotide thymidynyl entity contributes to the anticancer effect as well as the fact that a simple increase of the number of redox-active ferrocenyl centers in a given scaffold does not immediately lead to the improved anticancer effect. In general, antiproliferative activity studies are in agreement with our earlier observation of the high anticancer activity of MV ferrocenyl compounds.^16,61^ Oxidative stress (OS) resulting from ROS production is an important factor that takes part in the anticancer activity of organometallic compounds.^27,31,59,60^ Concerning that, the aim of the following studies was to examine whether studied compounds generate ROS in cancer cells and how the viability of the treated cells changes in the presence of *N*-acetyl cysteine (NAC) free-radical scavenger.^96^ Thus, we investigated the amount of ROS (OH^•^, O2^•–^, H_2_O_2_, ROO^•^) produced by compounds **1a** and **1c** and reference drugs at 20 μM concentration and 1 h treatment time in H1975 and A549 cells. The measurements were performed using fluorescent probe 5-(and-6)-chloromethyl-2′,7′-dichlorodihydrofluorescein diacetate-acetyl ester (CM-H2DCF-DA) (Figures 11 and S28, see the SI).

**Figure 12:**
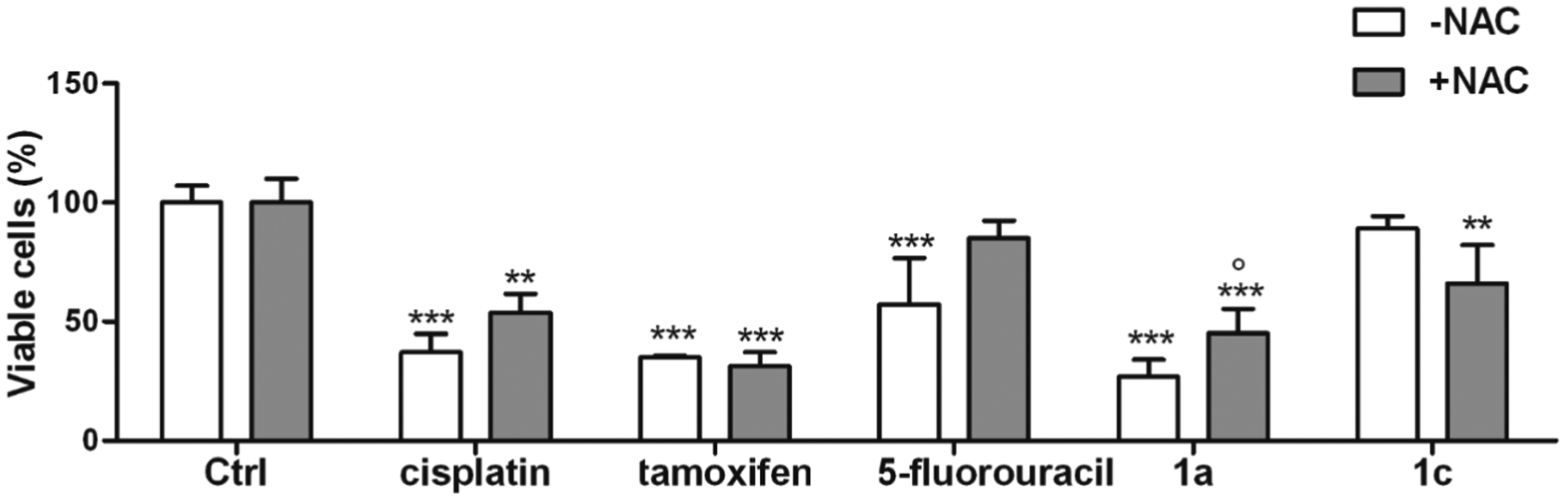
Viability of H1975 cells treated for 72 h with 20 μM of compounds **1a** and **1c** and reference drugs with or without 50 μM NAC. Cell viability was measured spectrophotometrically in triplicate. Data are mean ± SD (*n* = 3). **p < 0.01, ***p < 0.001: compound-treated cells *vs* respective untreated (Ctrl) cells; ^○^*p* < 0.05: compound-treated cells *vs* compound +NAC-treated cells.

Compounds **1a** and **1c** were more effective ROS generators than cisplatin, tamoxifen, and 5-fluorouracil in both cancer cell types. Of the two ferrocene compounds, the most effective ROS generator was binuclear complex **1a**. It generated about 1.6 and 2.5 times more ROS than **1c** in H1975 and A549 cells. Furthermore, **1a** was about 2 and 2.5 times more potent in ROS generation than reference drugs in H1975 and A549 cells. The addition of NAC had almost no effect on ROS generation by cisplatin, tamoxifen, and 5-fluorouracil. Oppositely, the ROS amount produced by **1a** in NAC-treated A549 and H1975 cells was approximatively between 0.4 and 0.8 times lower compared to A549 and H1975 NAC nontreated cells. This definitely pin points a key role of ROS in the mechanism of the anticancer action of **1a** and corroborates with EPR study results (see the Electron Paramagnetic Resonance (EPR) Spectroscopic Study section). Further support for the pivotal role of ROS in inducing compound **1a** anticancer activity was provided by the viability assays (Figures 12, S29, and S30, see the SI).

Cells treated with NAC were partially protected from the deleterious influence of compound **1a**. Accordingly, the viability of H1975 cells treated with NAC and compound **1a** increased approximately to 20% compared to cells treated only with compound **1a** (Figure 12) and an analogous increase was also observed for A549 and BEAS-2B cells (Figures S29 and S30, see the SI). These results once again pinpoint the induction of OS/ROS as a key factor responsible for the antiproliferative activity of **1a**.

## Conclusions

Two series of 1,2,3-triazole derivatives having one, two, or three ferrocenyl units in their molecular scaffolds were prepared. The synthetic approach utilized CuAAC reactions and enabled obtaining all representatives of a given series of compounds in a single synthetic step. The biferrocenyl (**1a**) and triferrocenyl (**2a**) complexes belong to weakly coupled class II mixed-valence systems according to Robin and Day.^19^ The EPR study shows that **1a** and **2a** are better ROS generators than mononuclear complex **1c**. Importantly, **1a** and **2a** showed higher anticancer activity toward A549 and H1975 NSCLC cells than their non-mixed-valence generating counterparts **1c** and **2c**. Their anticancer efficacy was similar to the efficacy of well-established anticancer drugs such as cisplatin, tamoxifen, and 5-fluorouracil. Of note, **1a** and **2a** are also characterized by very low toxicity against normal BEAS-2B cells. Observed with EPR studies, the ability for ROS generation of compounds **1a** and **2a** was further observed *in vitro* in A549 and H1975 cancer cells. Obtained data allow concluding that the highly deleterious effects of **1a** and **2a** in investigated cancer cells are primarily due to the ROS and oxidative stress generation. However, the increased ability for ROS generation is not the only mechanism through which these compounds work. This supposition is corroborated by the fact that thymidine derivative **1a** has higher anticancer activity than triferrocenyl compound **2a**, but of the two compounds, the latter one (**2a**) is more electron-rich and thus is more susceptible to oxidation in cancer cells. This observation underlines that the thymine portion of compound **1a** has also contributed to the anticancer effect. This might be a valuable starting point for the design of new ferrocenyl mixed-valence systems conjugated to nucleic acid components such as nucleosides or nucleotides.

## Experimental Section

### General Considerations

All preparations were carried out using standard Schlenk techniques. Chromatographic separations were performed using silica gel 60 (Merck, 230–400 mesh ASTM). Azidothymidine (AZT) and ethynylferrocene were purchased from a commercial supplier and used without prior purification. Solvents were of reagent grade and also used without prior purification. 3-Azidopropanoylferrocene was synthesized according to the literature guidelines.^97^^1^H NMR (600 MHz) and ^13^C{H} NMR (150 MHz) spectra were recorded with a Bruker ARX 600 spectrometer operating at 298 K in Fourier transform mode. Chemical shifts are given in δ units (ppm) using residual dimethyl sulfoxide (DMSO) (^1^H δ 2.50 ppm, ^13^C δ 39.5 ppm) or CDCl_3_ (^1^H δ 7.26 ppm, ^13^C δ 77.0 ppm) peaks as a reference. All of the mass spectra were recorded using a Synapt G2-Si mass spectrometer (Waters) equipped with an electrospray ionization (ESI) source and a quadrupole time-of-flight (quadrupole-TOF) mass analyzer. The mass spectrometer was operated in the positive ion detection mode. The measurements were performed with the capillary voltage set to 2.7 kV and the sampling cone voltage set to 20 V. The source temperature was 110 °C. To ensure the accuracy of mass measurements, data were collected in the centroid mode and mass was corrected during acquisition using leucine enkephalin solution as an external reference (Lock-Spray). The results of the measurements were processed using MassLynx 4.1 software (Waters) incorporated with the instrument. The IR spectra were recorded on a Fourier transform infrared (FTIR) Nexus Nicolet apparatus. Microanalyses were performed by Analytical Services of the Polish Academy of the Sciences (Łódź).

#### Synthesis of **1a**–**c**

A Schlenk tube charged with AZT (120 mg, 0.45 mmol, 1.0 equiv), ethynylferrocene (189 mg, 0.90 mmol, 2.0 equiv), CuI (120 mg, 0.63 mmol, 1.4 equiv), and *N*-bromosuccinimide (96 mg, 0.54 mmol, 1.2 equiv) was flushed with argon. Then, anhydrous THF (6 mL) and *N*,*N*-diisopropylethylamine (0.08 mL, 0.45 mmol, 1.0 equiv) were added. The resulting reaction mixture was protected against light and stirred at ambient temperature for 24 h. Then, 50 mL of 2% aqueous solution of hydrogen chloride was added and the mixture was extracted with dichloromethane (2 × 25 mL). The organic layer was separated, dried over anhydrous Na_2_SO_4_, and transferred to a round-bottomed flask, and all volatiles were evaporated under reduced pressure. After evaporation, the remaining oil was subjected to column chromatography on SiO_2_ (ethyl acetate/chloroform/methanol 35:30:3 v/v/v). Three fractions were collected. The first fraction contained compound **1a**, the second contained compound **1b**, and the third contained compound **1c**. Chromatographically purified compounds were crystallized from a mixture of dichloromethane/*n*-hexane to afford analytically pure samples. Compound **1a** was obtained as an orange crystalline solid in 39% (120 mg) yield, compound **1b** was obtained as a yellow crystalline solid in 9% (25 mg) yield, and compound **1c** was obtained as a yellow crystalline solid in 6% (12 mg) yield.

##### 3′-Deoxy-3′-(4-ferrocenyl-5-ethynylferrocenyl-1*H*-1,2,3-triazol-1-yl)thymidine (**1a**)

^1^H NMR (600 MHz, DMSO-*d*_6_): δ = 11.40 (s, 1H, NH thymine), 7.87 (s, 1H, H6 thymine), 6.54 (t, *J*_H,H_ = 6.6 Hz, 1H, H1′), 5.42 (m, 1H, H3′), 5.41 (t, *J*_H,H_ = 4.8 Hz, 1H, OH), 4.94 (pt, *J*_H,H_ = 1.8 Hz, 2H, C_5_H_4_ Fc), 4.77 (pq, *J*_H,H_ = 1.8 Hz, 2H, C_5_H_4_ Fc), 4.50 (pt, *J*_H,H_ = 1.8 Hz, 2H, C_5_H_4_ Fc), 4.44 (pt, *J*_H,H_ = 1.8 Hz, 2H, C_5_H_4_ Fc), 4.37 (s, 5H, C_5_H_5_ Fc), 4.36 (m, 1H, H4′), 4.15 (s, 5H, C_5_H_5_ Fc), 3.81 (m, 1H, H5′), 3.74 (m, 1H, H5′), 2.87 (m, 1H, H2′), 2.72 (m, 1H, H2′), 1.82 (s, 3H, CH_3_ thymine) ppm. ^13^C{^1^H} NMR (150 MHz, DMSO-*d*_6_): δ = 163.7, 150.5, 147.6, 136.1, 116.2, 109.7, 103.1, 84.45, 84.43, 74.3, 71.5, 71.4, 70.9, 69.97, 69.95, 69.3, 68.8, 66.3, 62.2, 61.4, 59.1, 36.4, 12.3 ppm. MS (TOF ES+): *m*/*z* = 686.1155 (M + H^+^) (calcd for C_34_H_32_N_5_O_4_Fe_2_: 686.1153). FTIR (CHCl_3_ ν [cm^–1^]): 3386 (OH), 3093, 3014, 2925, 2852, 2211 (C≡C), 1687 (C=O), 1468, 1411, 1272, 1219, 1104, 1052, 754. Anal. Calcd for C_34_H_31_N_5_O_4_Fe_2_: C, 59.59%; H, 4.56%; N, 10.22%. Found: C, 59,29%; H, 4.60%; N, 10.10%.

##### 3′-Deoxy-3′-(4-ferrocenyl-5-iodo-1*H*-1,2,3-triazol-1-yl)thymidine (**1b**)

^1^H NMR (600 MHz, CDCl_3_): δ = 8.47 (s, 1H, NH thymine), 7.29 (s, 1H, H6 thymine), 6.23 (t, *J*_H,H_ = 7.2 Hz, 1H, H1′), 5.50 (dt, *J*_H,H_ = 9.0, 3.6 Hz, 1H, H3′), 5.01 (s, 2H, C_5_H_4_ Fc), 4.47 (m, 1H, H4′), 4.37 (s, 2H, C_5_H_4_ Fc), 4.14 (s, 5H, C_5_H_5_ Fc), 4.04 (dt, *J*_H,H_ = 12.6, 2.4 Hz, 1H, H5′), 3.88 (ddd, *J*_H,H_ = 11.8, 9.0, 2.4 Hz, 1H, H5′), 3.52 (dd, *J*_H,H_ = 9.0, 2.4 Hz, 1H, OH), 3.20 (dt, *J*_H,H_ = 13.8, 8.4 Hz, 1H, H2′), 2.92 (dq, *J*_H,H_ = 13.8, 6.3, 3.0 Hz, 1H, H2′), 1.96 (s, 3H, CH_3_ thymine) ppm. ^13^C{^1^H} NMR (150 MHz, CDCl_3_): δ = 163.4, 150.4, 150.3, 139.0, 111.5, 91.6, 85.9, 75.4, 74.3, 69.7, 69.6, 69.1, 67.4, 67.3, 62.7, 60.3, 36.6, 29.8, 12.5 ppm. MS (TOF ES+): *m*/*z* = 604.0143 (M + H^+^) (calcd for C_22_H_23_N_5_O_4_IFe: 604.0144). FTIR (KBr ν [cm^–1^]): 3391 (OH), 3082, 2926, 1689 (C=O), 1468, 1410, 1272, 1228, 1105, 1050, 879. Anal. Calcd for C_22_H_22_N_5_O_4_IFe: C, 43.81%; H, 3.68%; N, 11.61%. Found: C, 43.85%; H, 3.61%; N, 11.64%.

##### 3′-Deoxy-3′-(4-ferrocenyl-1*H*-1,2,3-triazol-1-yl)thymidine (**1c**)

^1^H NMR (600 MHz, DMSO-*d*_6_): δ = 11.37 (s, 1H, NH thymine), 8.37 (s, 1H, H 1,2,3-triazole), 7.83 (s, 1H, H6 thymine), 6.44 (t, *J*_H,H_ = 6.6 Hz, 1H, H1′), 5.33 (m, 1H, H3′), 5.30 (t, *J*_H,H_ = 4.8 Hz, 1H, OH), 4.70 (s, 2H, C_5_H_4_ Fc), 4.31 (s, 2H, C_5_H_4_ Fc), 4.24 (m, 1H, H4′), 4.05 (s, 5H, C_5_H_5_ Fc), 3.72 (m, 1H, H5′), 3.65 (m, 1H, H5′), 2.77 (m, 1H, H2′), 2.68 (m, 1H, H2′), 1.82 (s, 3H, CH_3_ thymine) ppm. ^13^C{^1^H} NMR (150 MHz, DMSO-*d*_6_): δ = 163.7, 150.4, 145.5, 136.2, 120.1, 109.6, 84.4, 83.8, 75.7, 69.2, 68.2, 66.4, 66.3, 60.7, 59.1, 37.0, 12.2 ppm. MS (TOF ES+): *m*/*z* = 478.1169 (M + H^+^) (calcd for C_22_H_24_N_5_O_4_Fe: 478.1178). FTIR (KBr ν [cm^–1^]): 3180, 3115, 3053, 2949, 2835, 1693 (C=O), 1463, 1277, 1039. Anal. Calcd for C_22_H_23_N_5_O_4_Fe: C, 55.36%; H, 4.86%; N, 14.67%. Found: C, 55.24%; H, 4.90%; N, 14.39%.

#### Synthesis of **1c**

Ethynylferrocene (95 mg, 0.45 mmol, 1.2 equiv), sodium ascorbate (59 mg, 0.30 mmol, 0.8 equiv), and CuSO_4_·5H_2_O (20 mg, 0.08 mmol, 0.2 equiv) were added to a stirred solution of AZT (99 mg, 0.37 mmol, 1.0 equiv) in 4 mL of THF/H_2_O (1/1 v/v). The resulting reaction mixture was stirred at 60 °C for 6 h. Then, all volatiles were evaporated under reduced pressure and subsequently treated with 15 mL of DCM. The resulting suspension was filtered off through a Schott funnel, and the yellow filtrate was washed with 150 mL of distilled water and 30 mL of DCM. The resulting material was dried under reduced pressure overnight to afford an analytically pure sample as a yellow crystalline solid in 69% (122 mg) yield.

#### Synthesis of **2a**–**c**

A Schlenk tube charged with 3-azidopropionylferrocene (150 mg, 0.53 mmol, 1.0 equiv), ethynylferrocene (223 mg, 1.06 mmol, 2.0 equiv), CuI (141 mg, 0.74 mmol, 1.4 equiv), and *N*-bromosuccinimide (112 mg, 0.63 mmol, 1.2 equiv) was flushed with argon. Then, anhydrous THF (6 mL) and *N*,*N*-diisopropylethylamine (0.09 mL, 0.53 mmol, 1.0 equiv) were added. The resulting reaction mixture was protected against light and stirred at ambient temperature for 24 h. Then, 60 mL of 2% aqueous solution of hydrogen chloride was added and the mixture was extracted with dichloromethane (2 × 25 mL). The organic layer was separated, dried over anhydrous Na_2_SO_4_, and transferred to a round-bottomed flask, and all volatiles were evaporated under reduced pressure. After evaporation, the remaining oil was subjected to column chromatography on SiO_2_ (ethyl acetate/*n*-hexane 2:3 v/v). Two fractions were collected. The first fraction contained a mixture of compounds **2a** and **2b**, whereas the second contained compound **2c**. Compound **2c** was obtained as an orange crystalline solid in 15% (39 mg) yield following crystallization from a mixture of dichloromethane/*n*-hexane. The mixture of compounds **2a** and **2b** was subjected to column chromatography on SiO_2_ (dichloromethane/ethyl acetate/acetone 300:7:2 v/v/v). Two fractions were collected. The first fraction contained compound **2a**, and the second contained compound **2b**. Chromatographically purified products were crystallized from a mixture of dichloromethane/*n*-hexane to afford analytically pure samples. Compound **2a** was obtained as an orange crystalline solid in 22% (83 mg) yield, and compound **2b** was obtained as an orange crystalline solid in 24% (78 mg) yield.

##### 1-(3-Propionylferrocenyl)-4-ferrocenyl-5-ethynylferrocenyl-1*H*-1,2,3-triazole (**2a**)

^1^H NMR (600 MHz, DMSO-*d*_6_): δ = 4.94 (pt, *J*_H,H_ = 1.8 Hz, 2H, C_5_H_4_ Fc), 4.84 (pt, *J*_H,H_ = 1.8 Hz, 2H, C_5_H_4_ Fc), 4.75 (pt, *J*_H,H_ = 1.8 Hz, 2H, C_5_H_4_ Fc), 4.73 (t, *J*_H,H_ = 6.6 Hz, 2H, N–CH_2_), 4.60 (pt, *J*_H,H_ = 1.8 Hz, 2H, C_5_H_4_ Fc), 4.49 (pt, *J*_H,H_ = 1.8 Hz, 2H, C_5_H_4_ Fc), 4.42 (pt, *J*_H,H_ = 1.8 Hz, 2H, C_5_H_4_ Fc), 4.38 (s, 5H, C_5_H_5_ Fc), 4.20 (s, 5H, C_5_H_5_ Fc), 4.12 (s, 5H, C_5_H_5_ Fc), 3.54 (t, *J*_H,H_ = 6.6 Hz, 2H, CH_2_C(=O)) ppm. ^13^C{^1^H} NMR (150 MHz, CDCl_3_): δ = 200.4, 148.3, 117.4, 102.4, 78.3, 75.0, 72.8, 71.8, 71.6, 70.2, 70.1, 69.8, 69.7, 69.4, 68.9, 66.9, 63.2, 44.0, 39.0 ppm. MS (TOF ES+): *m*/*z* = 702.0604 (M + H^+^) (calcd for C_37_H_32_N_3_OFe_3_: 702.0594). FTIR (KBr ν [cm^–1^]): 3091, 2921, 2853, 2214 (C≡C), 1667 (C=O), 1585, 1541, 1455, 1410, 1378, 1250, 1213, 1105, 1000, 820, 486. Anal. Calcd for C_37_H_31_N_3_OFe_3_: C, 63.38%; H, 4.46%; N, 5.99%. Found: C, 63.33%; H, 4.67%; N, 6.23%.

##### 1-(3-Propionyloferrocenyl)-4-ferrocenyl-5-iodo-1*H*-1,2,3-triazole (**2b**)

^1^H NMR (600 MHz, DMSO-*d*_6_): δ = 4.91 (pt, *J*_H,H_ = 1.8 Hz, 2H, C_5_H_4_ Fc), 4.84 (pt, *J*_H,H_ = 1.8 Hz, 2H, C_5_H_4_ Fc), 4.66 (t, *J*_H,H_ = 6.6 Hz, 2H, N-CH_2_), 4.60 (pt, *J*_H,H_ = 1.8 Hz, 2H, C_5_H_4_ Fc), 4.36 (pt, *J*_H,H_ = 1.8 Hz, 2H, C_5_H_4_ Fc), 4.21 (s, 5H, C_5_H_5_ Fc), 4.10 (s, 5H, C_5_H_5_ Fc), 3.48 (t, *J*_H,H_ = 6.6 Hz, 2H, CH_2_C(=O)) ppm. ^13^C{^1^H} NMR (150 MHz, DMSO-*d*_6_): δ = 199.8, 148.0, 80.2, 78.3, 75.5, 72.4, 69.6, 69.2, 69.1, 68.4, 66.6, 45.2, 38.2 ppm. MS (TOF ES+): *m*/*z* = 619.9589 (M + H^+^) (calcd for C_25_H_23_N_3_OIFe_2_: 619.9585). FTIR (KBr ν [cm^–1^]): 3084, 2952, 2922, 2852, 1669, 1657, 1566, 1455, 1399, 1252, 1223, 1105, 1065, 998, 878, 817. Anal. Calcd for C_25_H_22_N_3_OIFe_2_: C, 48.50%; H, 3.58%; N, 6.79%. Found: C, 48.59%; H, 3.36%; N, 6.64%.

##### 1-(3-Propionyloferrocenyl)-4-ferrocenyl-1*H*-1,2,3-triazole (**2c**)

^1^H NMR (600 MHz, DMSO-*d*_6_): δ = 8.19 (s, 1H, H 1,2,3-triazole), 4.83 (pt, *J*_H,H_ = 1.8 Hz, 2H, C_5_H_4_ Fc), 4.68 (pt, *J*_H,H_ = 1.8 Hz, 2H, C_5_H_4_ Fc), 4.67 (t, *J*_H,H_ = 6.6 Hz, 2H, N–CH_2_), 4.59 (pt, *J*_H,H_ = 1.8 Hz, 2H, C_5_H_4_ Fc), 4.28 (pt, *J*_H,H_ = 1.8 Hz, 2H, C_5_H_4_ Fc), 4.14 (s, 5H, C_5_H_5_ Fc), 4.01 (s, 5H, C_5_H_5_ Fc), 3.44 (t, *J*_H,H_ = 6.6 Hz, 2H, CH_2_C(=O)) ppm. ^13^C{^1^H} NMR (150 MHz, CDCl_3_): δ = 200.8, 146.3, 120.9, 78.1, 75.6, 72.9, 70.0, 69.6, 69.3, 68.6, 66.7, 44.6, 39.6 ppm. MS (TOF ES+): *m*/*z* = 494.0620 (M + H^+^) (calcd for C_25_H_24_N_3_OFe_2_: 494.0618). FTIR (KBr ν [cm^–1^]): 3107, 3075, 1659 (C=O), 1452, 1376, 1252, 1105, 1080, 1049, 999, 823, 812, 482. Anal. Calcd for C_25_H_23_N_3_OFe_2_: C, 60.89%; H, 4.70%; N, 8.52%. Found: C, 60.71%; H, 4.95%; N, 8.61%.

#### Synthesis of **2c**

A Schlenk tube charged with 3-azidopropanoylferrocene (71 mg, 0.25 mmol, 1.0 equiv), ethynylferrocene (63 mg, 0.30 mmol, 1.2 equiv), sodium ascorbate (40 mg, 0.20 mmol, 0.8 equiv), and CuSO_4_·5H_2_O (13 mg, 0.05 mmol, 0.2 equiv) was flushed with argon. Then, 6 mL of THF/H_2_O (1/1 v/v) was added. The resulting reaction mixture was stirred at ambient temperature for 24 h. Then, 50 mL of water was added and the mixture was extracted with chloroform (3 × 25 mL). The organic layer was separated, dried over anhydrous Na_2_SO_4_, and transferred to a round-bottomed flask, and all volatiles were evaporated under reduced pressure. After evaporation, the remaining oil was subjected to column chromatography on SiO_2_ (chloroform/ethyl acetate 15:2 v/v). Chromatographically purified product was crystallized from a mixture of dichloromethane/*n*-hexane to afford an analytically pure sample. Compound **2c** was obtained as an orange crystalline solid in 75% (93 mg) yield.

### X-ray Structure Analysis

Good-quality single crystals of **1a**, **2a**, and **2c** were selected for the X-ray diffraction experiments at *T* = 100(2) K. Diffraction data were collected on an Agilent Technologies SuperNova Dual Source diffractometer with CuKα radiation (λ = 1.54184 Å) using CrysAlis RED software.^98^ Analytical absorption correction using a multifaceted crystal model based on expressions derived by Clark and Reid (**1a** and **2c**) and numerical absorption correction based on Gaussian integration over a multifaceted crystal model (**2a**) were applied.^98,99^ The structural determination procedure was carried out using the SHELX package.^100^ The structures were solved with an intrinsic phasing method, and then, successive least-squares refinement was carried out based on the full-matrix least-squares method on *F*^2^ using the SHELXL program.^100^ All H-atoms were positioned geometrically with C–H bond lengths equal to 0.93, 0.96, 0.97, and 0.98 Å for the aromatic, methyl, methylene, and methine H-atoms, respectively, and constrained to ride on their parent atoms with *U*_iso_(H) = *xU*_eq_(C), where *x* = 1.2 for the aromatic, methylene, and methine and *x* = 1.5 for the methyl H-atoms. In the case of **1a**, the N–H and O–H bond lengths were equal to 0.86 and 0.82 Å for the amine and hydroxyl H-atoms, respectively, and constrained to ride on their parent atoms with *U*_iso_(H) = *xU*_eq_(N,O), where *x* = 1.2 for the amine and 1.5 for the hydroxyl H-atoms, respectively. Nine out of twelve cyclopentadienyl rings in **2a** were subject to RIGU restraints, whereas on the N1A, N2A, N2B, C4B, and C26B atoms, ISOR restraints were additionally applied. These types of restraints were also used during refinement of **1a**. RIGU was applied to restrain cyclopentadienyl moiety defined by atoms C20A–C24A, while atoms C19A–C24A, C13B, and C20B were subject to ISOR restraints. In the case of **1a**, a few distinct peaks on the difference Fourier map are indicating the presence of disordered solvent molecules. All attempts to model disordered solvents used for crystallization failed. Therefore, solvent contribution has been removed by applying the appropriate MASK procedure in the Olex2 program.^101^ The calculated void volume was approximately 947.9 Å^3^ occupied by 187.0 electrons per unit cell. The figures for this publication were prepared using the Olex2 program.^101^

### Electrochemistry

Measurements on 1.0 mmol·L^–1^ solutions of analytes **1a**, **1c**, **2a**, and **2c** in anhydrous dichloromethane solutions, containing 0.1 mol·L^–1^ [NBu_4_][B(C_6_F_5_)_4_] as the supporting electrolyte, were conducted under an atmosphere of argon at 25 °C. A three-electrode cell, which utilized a Pt auxiliary electrode, a glassy carbon working electrode (surface area 0.031 cm^2^), and an Ag/Ag^+^ (0.01 mol·L^–1^ AgNO_3_) reference electrode, was used as described in refs (82) and (102−104). Successive experiments under the same experimental conditions showed that all formal potentials were reproducible within ±5 mV. Experimental potentials were referenced against an Ag/Ag^+^ reference electrode, but results presented are referenced against the ferrocene [FcH/FcH^+^ couple = 220 mV *vs* Ag/Ag^+^, Δ*E*_p_ = 61 mV; FcH = Fe(η^5^-C_5_H_5_)_2_] as an internal standard.^82^ When decamethylferrocene [Fc* = Fe(η^5^-C_5_Me_5_)_2_] was used as an internal standard, the experimentally measured potentials were converted into *Evs* FcH/FcH^+^ (under our conditions, the Fc*/Fc*^+^ couple was at −614 mV *vs* FcH/FcH^+^, Δ*E*_p_ = 60 mV).

### Spectroelectrochemistry

The spectroelectrochemical measurements of **1a** and **2a** in anhydrous tetrahydrofuran containing [NBu_4_][B(C_6_F_5_)_4_] (0.1 mol·L^–1^) as the supporting electrolyte were performed at 25 °C in an optically transparent thin-layer electrochemistry (OTTLE) cell^87^ with quartz windows (UV–vis/NIR, compounds **1a** and **2a**) by a Varian Cary 5000 spectrophotometer or CaF_2_ windows (IR, **1a**) with a Nicolet IR200 spectrometer (Thermo Fisher). Between the spectroscopic measurements, the applied potentials were increased stepwise using step heights of 25, 50, or 100 mV. At the end of the measurements, the analyte was reduced at −500 mV *vs* Ag/AgCl for 30 min, and an additional spectrum was recorded to prove the reversibility of the oxidations.

### Computational Details

Structures of **1a**, **1c**, **2a**, and **2c** (oxidized/reduced forms) were optimized using the gradient corrected pure functional BLYP, with an effective core potential (ECP) basis set from the Los Alamos National Laboratory, LANL2DZ,^90^ on Fe atoms and with 6-31+G(d) basis set on other elements. All computational experiments were conducted using Gaussian 16 software.^91^ The search for conformers was performed by molecular modeling software PCMODEL 10.0 (using the MMX force field).^105^ Frequency calculations were performed to calculate thermal corrections to Gibbs free energies (at 298.15 K). Implicit solvation was modeled using the SCRF = SMD continuum solvation method at the (U)BLYP/6-31+G(d)/LANL2DZ level in dichloromethane (ε = 8.93) as a model solvent.^106^

### EPR Measurements

EPR measurements were performed using a CW X-band EMXplus spectrometer with a PremiumX microwave bridge and a high-sensitivity resonator (Bruker, Germany). The EPR spectra were registered at 100 kHz modulation and a microwave power of 5 mW at room temperature. An NMR teslameter (Bruker, Germany) was used for precise *g* value determination. For *in situ* EPR spectroelectrochemical experiments, a three-electrode EPR flat cell was used. A laminated gold mesh (Goodfellow, U.K.) as the working electrode, an AgCl-coated silver wire as the pseudoreference electrode, and a platinum wire as the counter electrode were used in spectroelectrochemical experiments. The 0.1 M [N(Bu)_4_][B(C_6_F_5_)_4_] in THF (anhydrous, ≥99.9%, inhibitor-free, Sigma-Aldrich) was used as the supporting electrolyte. Cell assembling and the measurements were performed under an inert (nitrogen) atmosphere. In the spin-trapping experiments, dimethylformamide (DMF, anhydrous, ≥99.8%, Sigma-Aldrich) solutions were bubbled with air, oxygen, or nitrogen for 2 h. 50 mM spin trap 5,5-dimethyl-1-pyrroline *N*-oxide (DMPO, ≥99.0% (GC), Dojindo, Japan) and 1.5 mM ferrocene compound were added to the solution one after another.

### Biological Assays

#### Cells

Human non-small-cell lung cancer cell lines A549 and H1975 and the human bronchial epithelial BEAS-2B cell line were purchased from ATCC (Manassas, VA). Cells were cultured in Roswell Park Memorial Institute (RPMI)-1640 media supplemented with 10% v/v fetal bovine serum, 100 U·mL^–1^ penicillin, and 100 μg·mL^–1^ streptomycin. Cells were grown in a humidified atmosphere at 37 °C and 5% CO_2_.

### Reactive Oxygen Species (ROS) Generation

Cells were incubated for 1 h in a fresh medium or in a medium containing 20 μM of compounds **1a** and **1c** and tamoxifen, 5-fluorouracil, and cisplatin, alone or together with 50 μM *N*-acetyl cysteine (NAC). Then, detached cells were resuspended in 0.5 mL of phosphate-buffered saline (PBS) containing 10 μM·L^–1^ fluorescent probe 5-(and-6)-chloromethyl-2′,7′-dichlorodihydrofluorescein diacetate-acetyl ester (CM-H2DCFDA) and incubated for 15 min at 37 °C. Afterward, the incubation cells were centrifuged at 13,000 rpm for 30 s and resuspended in 0.5 mL of PBS. The fluorescence of each sample (index of ROS levels) was read at 488 nm (λ_excitation_) and 520 nm (λ_emission_). The results were expressed as DCF fluorescence per mg cell proteins normalized *vs* control.

### Cell Viability with the Crystal Violet Assay

Crystal violet staining was used to assess cell viability. Cells were seeded in a 24-well plate and incubated with 20 μM concentration of compounds **1a**, **1c**, **2a**, and **2c** and tamoxifen, 5-fluorouracil, and cisplatin, with or without 50 μM NAC. After 72 h, the medium was discarded and cells were stained for 30 min with 5% w/v crystal violet solution in 66% v/v methanol, 200 μL per well. After staining, the crystal violet solution was removed, and the 24-well plate was washed with water to eliminate the excess solution. When dried, the plates were photographed. Quantitation of crystal violet staining was performed after solubilizing the dye in 10% acetic acid, 400 μL per well, and reading the absorbance of each well at 540 nm (HT Synergy 96-well microplate reader, Bio-Tek Instruments, Winooski, VT). The relative absorbance of untreated cells was considered as 100% viability; results were expressed as a percentage of viable cells *vs* untreated cells. To calculate IC_50_, cells were incubated 72 h with increasing concentrations (1 nM, 10 nM, 100 nM, 1 μM, 10 μM, 100 μM, 1 mM) of compounds **1a**, **1c**, **2a**, and **2c** and tamoxifen, 5-fluorouracil, and cisplatin. IC_50_ was defined as the concentration of each compound that reduced the cell viability to 50% compared to untreated cells, producing 50% cell death (GraphPad Prism, version 5).

## Supporting Information Available

Spectra (^1^H/^13^C/NMR), EPR spectra, crystal data, and biological data (PDF)

## Accession Codes

CCDC 2158730–2158732 contain the supplementary crystallographic data for this paper. These data can be obtained free of charge via www.ccdc.cam.ac.uk/data_request/cif, or by emailing data_request@ccdc.cam.ac.uk, or by contacting The Cambridge Crystallographic Data Centre, 12 Union Road, Cambridge CB2 1EZ, UK; fax: +44 1223 336033.

CCDC 2158732 (**1a**), 2158730 (**2a**), and 2158731 (**2c**) contain the supporting crystallographic data for this paper. The data can be obtained free of charge from the Cambridge Crystallographic Data Center via www.ccdc.cam.ac.uk/structures or by emailing data_request@ccdc.cam.ac.uk or by contacting The Cambridge Crystallographic Data Centre, 12 Union Road, Cambridge CB2 1EZ, U.K.; fax: +44 1223 336033.
