# Supplementary material for: Electronic Coupling in 1,2,3-Triazole Bridged Ferrocenes and Its Impact on Reactive Oxygen Species Generation and Deleterious Activity in Cancer Cells
Source: Inorg Chem. 2022 Jun 14;61(25):9650–66. doi: 10.1021/acs.inorgchem.2c01110 (PMC9490837; doi:10.1021/acs.inorgchem.2c01110)
Supplement: Supplementary file 1 — ic2c01110_si_001.pdf [file ic2c01110_si_001.pdf]

SUPPORTING INFORMATION

Electronic coupling in 1,2,3-triazole bridged ferrocenes and its impact on reactive oxygen species generation and deleterious activity in cancer cells

Przemysław Biegański,[a] Eduard Kovalski,[b] Noel Israel,[c] Evgenia Dmitrieva,[c] Damian Trzybiński,[d] Krzysztof Woźniak,[d] Valerije Vrček,[e] Martina Godel,[f] Chiara Riganti,[f] Joanna Kopecka, [f] Heinrich Lang,[b,g] and Konrad Kowalski*[a]

[a] *Faculty of Chemistry, Department of Organic Chemistry, University of Łódź, Tamka 12, 91-403 Łódź, Poland;*

[b] *Technische Universität Chemnitz, Fakultät für Naturwissenschaften, Institut für Chemie, Anorganische Chemie, Straße der Nationen 62, D-09107 Chemnitz, Germany;*

[c] *Leibniz Institute for Solid State and Materials Research (IFW Dresden), Helmholtzstraße 20, D-01069 Dresden, Germany;*

[d] *Faculty of Chemistry, Biological and Chemical Research Centre, University of Warsaw, Żwirki i Wigury 101, 02-089 Warszawa, Poland;*

[e] *University of Zagreb, Faculty of Pharmacy and Biochemistry, Department of Organic Chemistry, 10000 Zagreb, Croatia;*

[f] *Department of Oncology, University of Torino via Santena 5/bis - 10126 Turin – Italy;*

[g] *MAIN Research Center, Technische Universität Chemnitz, Rosenbergstraße 6, 09126 Chemnitz, Germany*

* Corresponding author: Konrad Kowalski (konrad.kowalski@chemia.uni.lodz.pl)

Contents

Fig. S1 ^{1}H-NMR spectrum of **1a** in DMSO-d_6 (600 MHz) **S 4**

Fig. S2 ^{1}H-NMR spectrum of **1b** in $CDCl_3$ (600 MHz) **S 5**

Fig. S3 ^{1}H-NMR spectrum of **1c** in DMSO-d_6 (600 MHz) **S 6**

Fig. S4 ^{1}H-NMR spectrum of **2a** in DMSO-d_6 (600 MHz) **S 7**

Fig. S5 ^{1}H-NMR spectrum of **2b** in DMSO-d_6 (600 MHz) **S 8**

Fig. S6 ^{1}H-NMR spectrum of **2c** in DMSO-d_6 (600 MHz) **S 9**

Fig. S7 ^{13}C{^{1}H }-NMR spectrum of **1a** in DMSO-d_6 (150 MHz) **S 10**

Fig. S8 ^{13}C{^{1}H }-NMR spectrum of **1b** in $CDCl_3$ (150 MHz) **S 11**

Fig. S9 ^{13}C{^{1}H }-NMR spectrum of **1c** in DMSO-d_6 (150 MHz) **S 12**

Fig. S10 ^{13}C{^{1}H }-NMR spectrum of **2a** in $CDCl_3$ (150 MHz) **S 13**

Fig. S11 ^{13}C{^{1}H }-NMR spectrum of **2b** in DMSO-d_6 (150 MHz) **S 14**

Fig. S12 ^{13}C{^{1}H }-NMR spectrum of **2c** in $CDCl_3$ (150 MHz) **S 15**

Fig. S13 Cyclic voltammograms of **1c** and **2c** **S 16**

Fig. S14. Deconvolution of the NIR absorptions of **[1a]$^+$** and **[2a]$^+$** **S 17**

Fig. S15. HOMO orbitals of **1a**, **1c**, **2a**, and **2c** calculated at the BLYP/6-31+G(d)/LanL2DZ **S 18**

Fig. S16. SOMO and SOMO-1 orbitals in dicationic species **1a^{2+}** **S 19**

Fig. S17. EPR spectra of DMPO-adducts **1a** and (b) **2a** under air conditions. **S 20**

Fig. S18 EPR spectra of DMPO-adducts of **1a** under (a) O_2- and (b) N_2- saturated conditions **S 21**

Fig. S19 Cell survival curves of A549 cells treated with **1a** and **1c** **S 22**

Fig. S20 Cell survival curves of A549 cells treated with **2a** and **2c** **S 23**

Fig. S21 Cell survival curves of A549 cells treated with cisplatin, tamoxifen and 5-fluorouracil **S 24**

Fig. S22 Cell survival curves of H1975 cells treated with **1a** and **1c** **S 25**

Fig. S23 Cell survival curves of H1975 cells treated with **2a** and **2c** **S 26**

Fig. S24 Cell survival curves of H1975 cells treated with cisplatin, tamoxifen and 5-fluorouracil **S 27**

Fig. S25 Cell survival curves of Beas-2b cells treated with **1a** and **1c** **S 28**

Fig. S26 Cell survival curves of Beas-2b cells treated with **2a** and **2c** **S 29**

Fig. S27 Cell survival curves of Beas-2b cells treated with cisplatin, tamoxifen and 5-fluorouracil **S 30**

Fig. S28 The effect of NAC on ROS generation in A549 cells treated with **1a** and **1c** **S 31**

Fig. S29 Viability of A549 cells in the presence or absence of NAC **S 32**

Fig. S30 Viability of BEAS-2B cells in the presence or absence of NAC **S 33**

Table S1 Crystal data and structure refinement for investigated compounds **S 34**

Table S2 Bond lengths for **1a** [Å] **S 35**

Table S3 Values of valence angles for **1a** [°] **S 37**

Table S4 Values of torsion angles for **1a** [°] **S 43**

Table S5 Bond lengths for **2a** [Å] **S 47**

Table S6 Values of valence angles for **2a** [°] **S 49**

Table S7 Values of torsion angles for **2a** [°] **S 56**

Table S8 Bond lengths for **2c** [Å] **S 60**

Table S9 Values of valence angles for **2c** [°] **S 61**

Table S10 Values of torsion angles for **2c** [°] **S 64**

Table S11 Values of angle of relative rotation of the cyclopentadienyl rings (τ) within individual ferrocene moieties in the crystals of investigated compounds **S 66**

Table S12 Cremer & Pople and pseudorotation parameters calculated for the pentafuranose rings present in **1a** **S 67**

Table S13. Experimental (IR_{exp}) and calculated (IR_{calc}) carbon-carbon triple bond stretch **S 68**

Equation 1 **S 69**

Details on DFT Calculations **S 70**

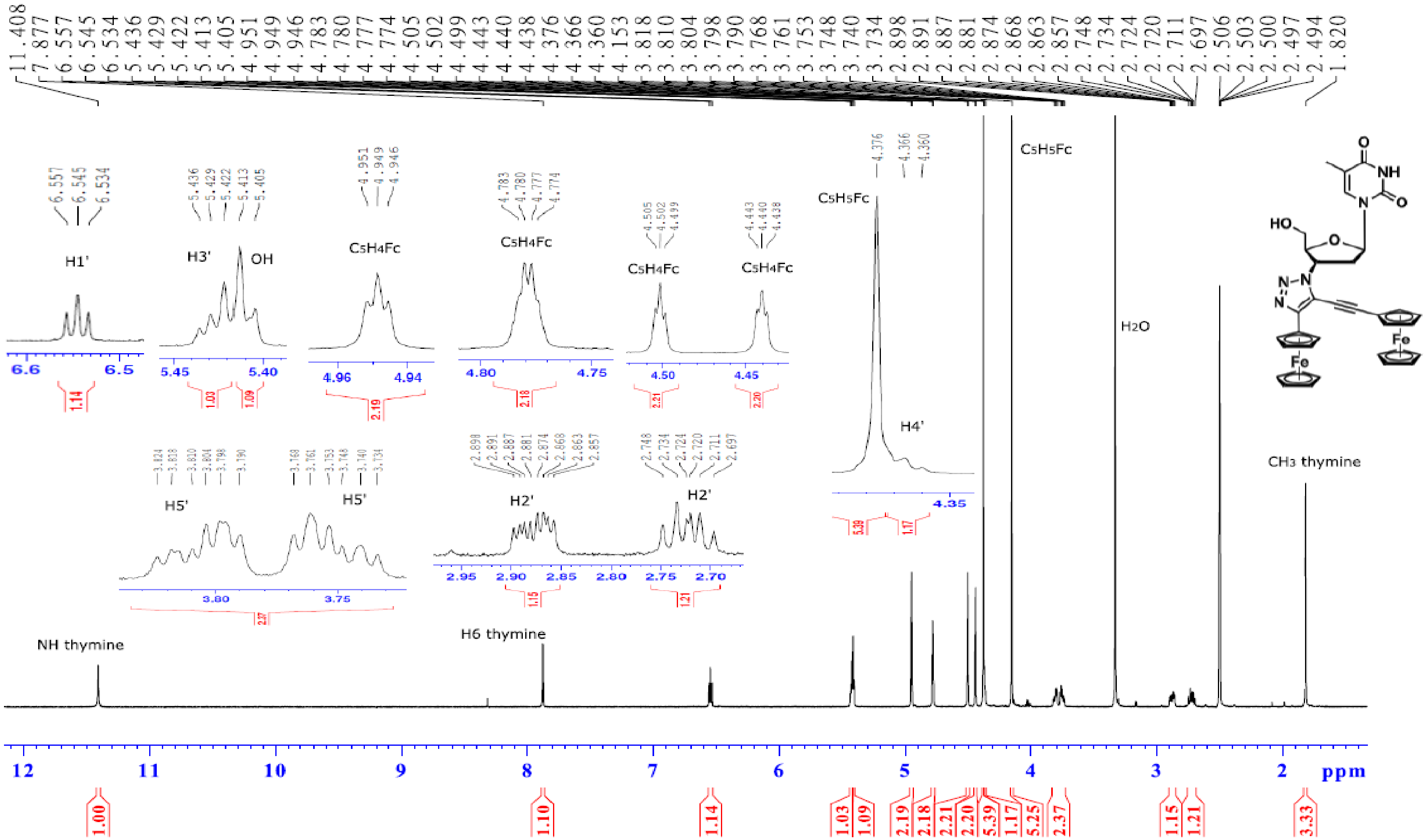

Fig. S1 ^{1}H-NMR spectrum of **1a** in DMSO-d6 (600 MHz)

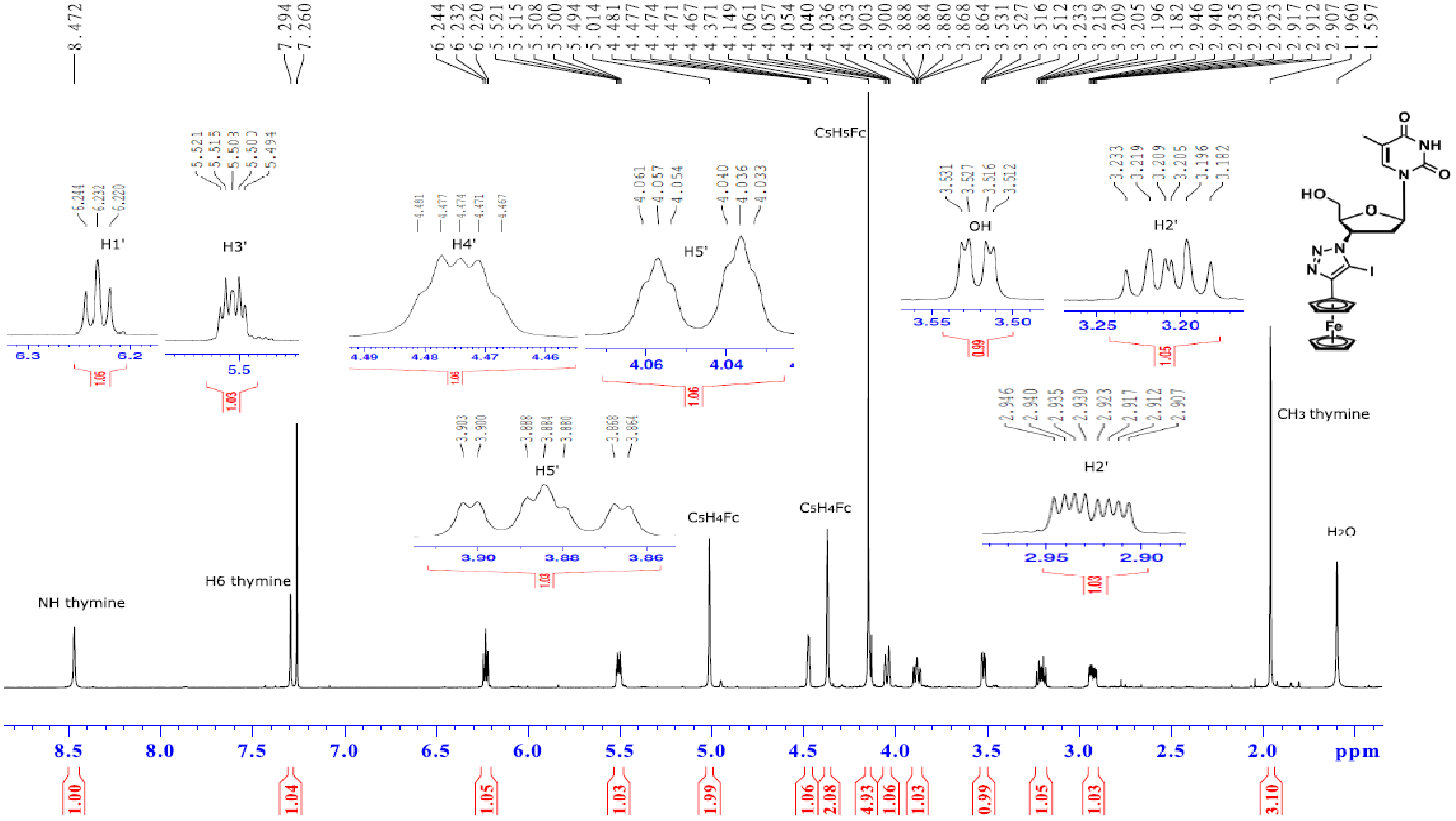

Fig. S2 ^{1}H-NMR spectrum of **1b** in $CDCl_3$ (600 MHz)

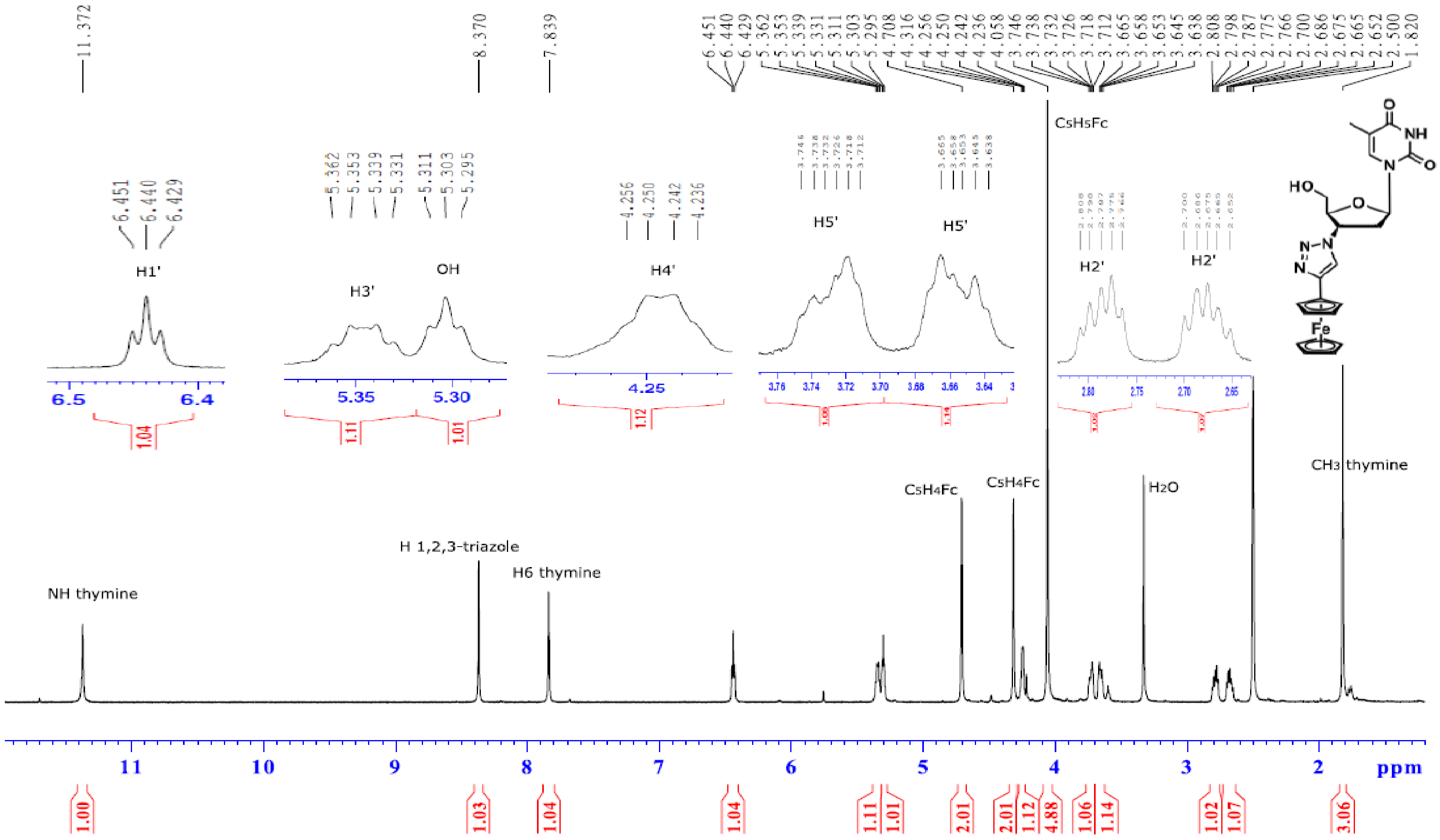

Fig. S3 ^{1}H-NMR spectrum of **1c** in DMSO-d6 (600 MHz)

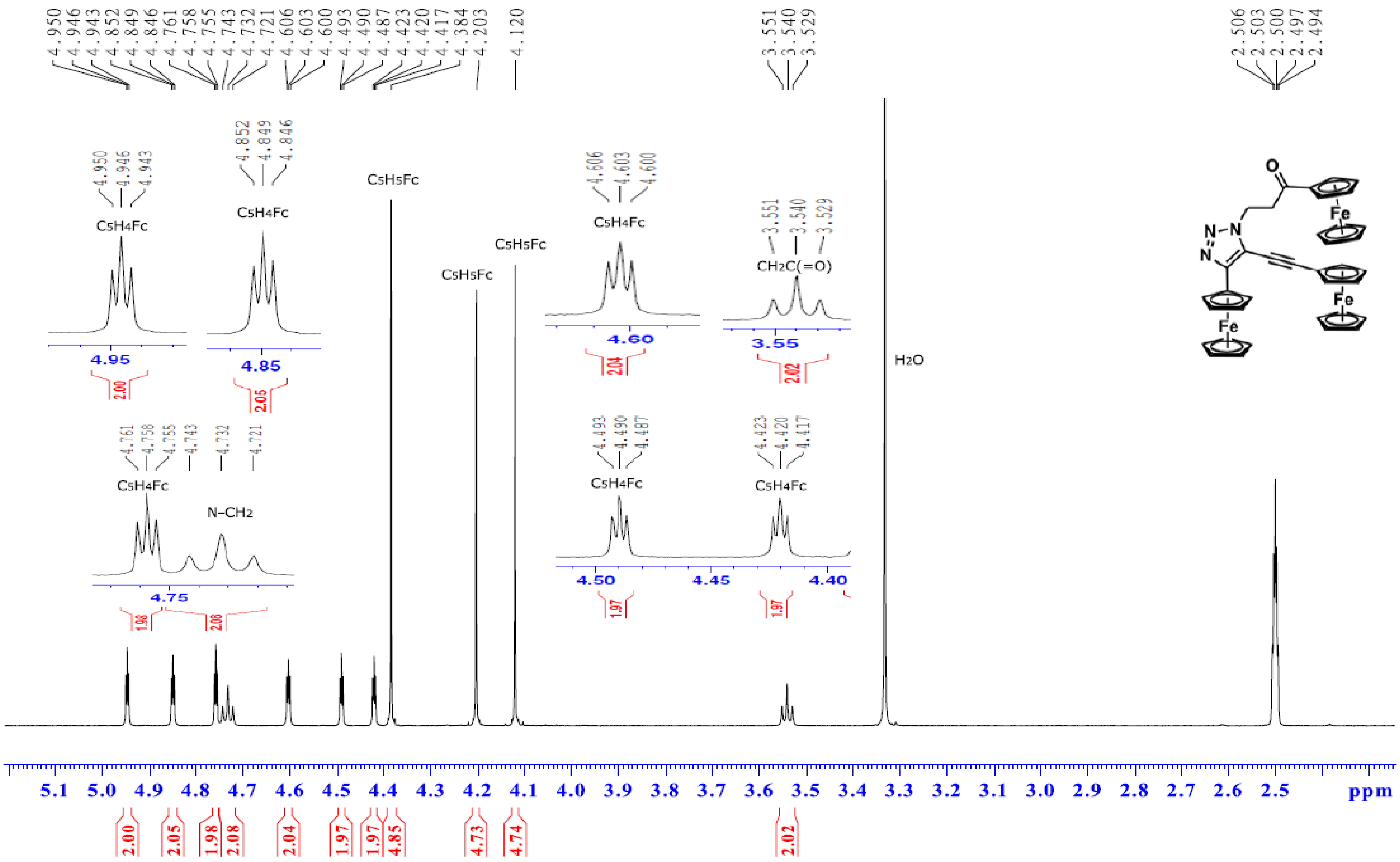

Fig. S4 ^{1}H-NMR spectrum of **2a** in DMSO-d_6 (600 MHz)

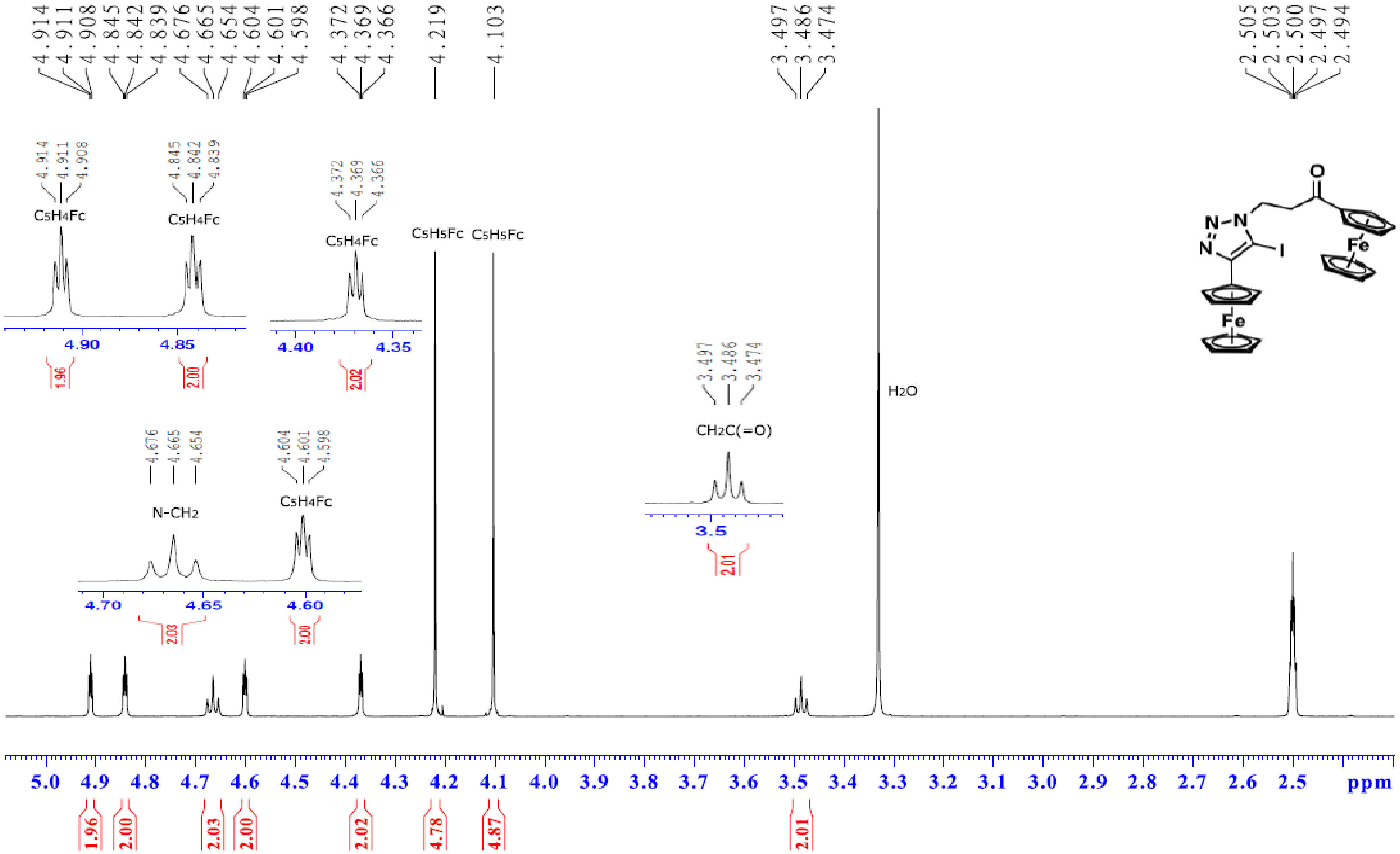

Fig. S5 ^{1}H-NMR spectrum of **2b** in DMSO-d$_6$ (600 MHz)

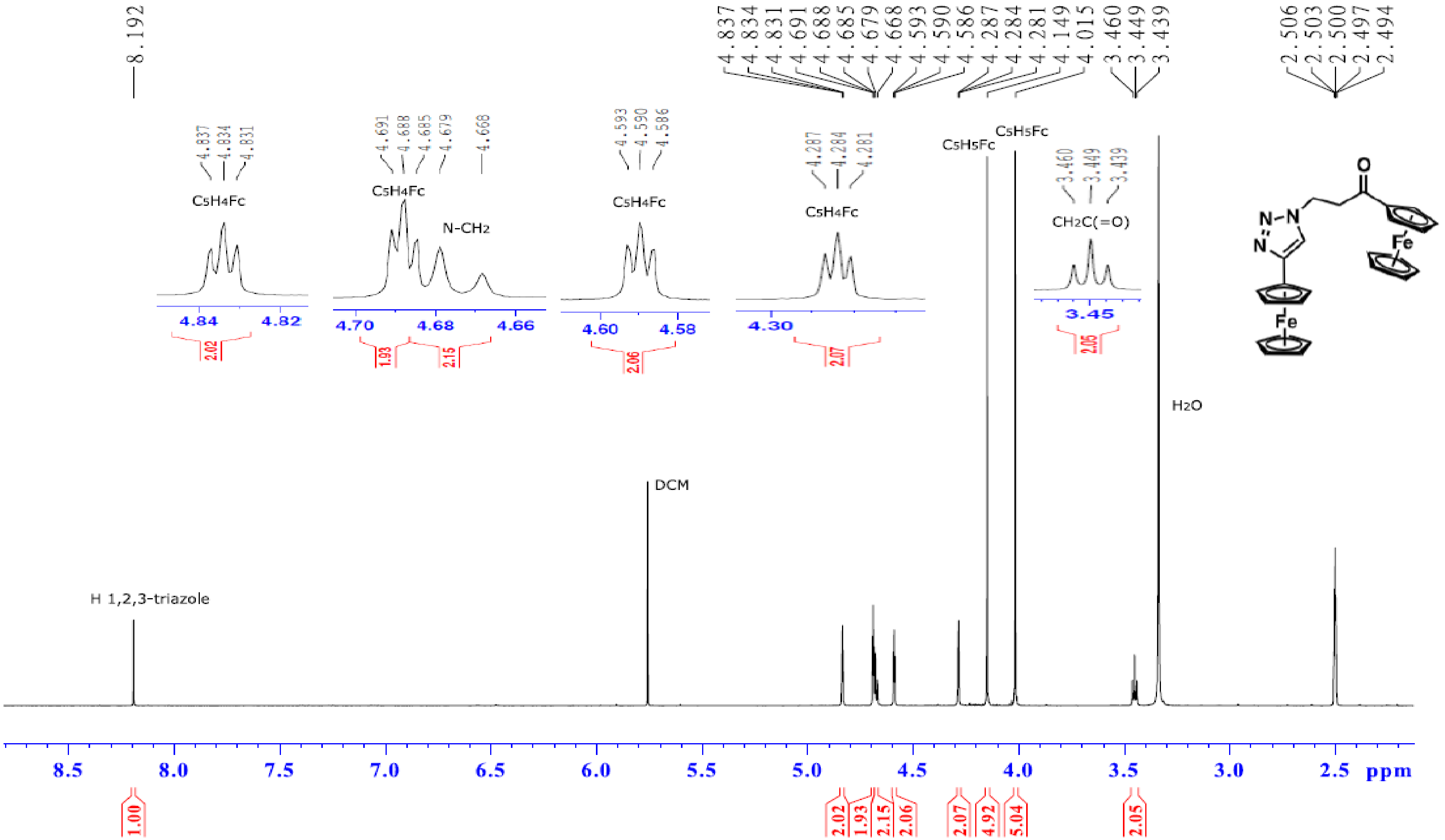

Fig. S6 ^{1}H-NMR spectrum of **2c** in DMSO-d6 (600 MHz)

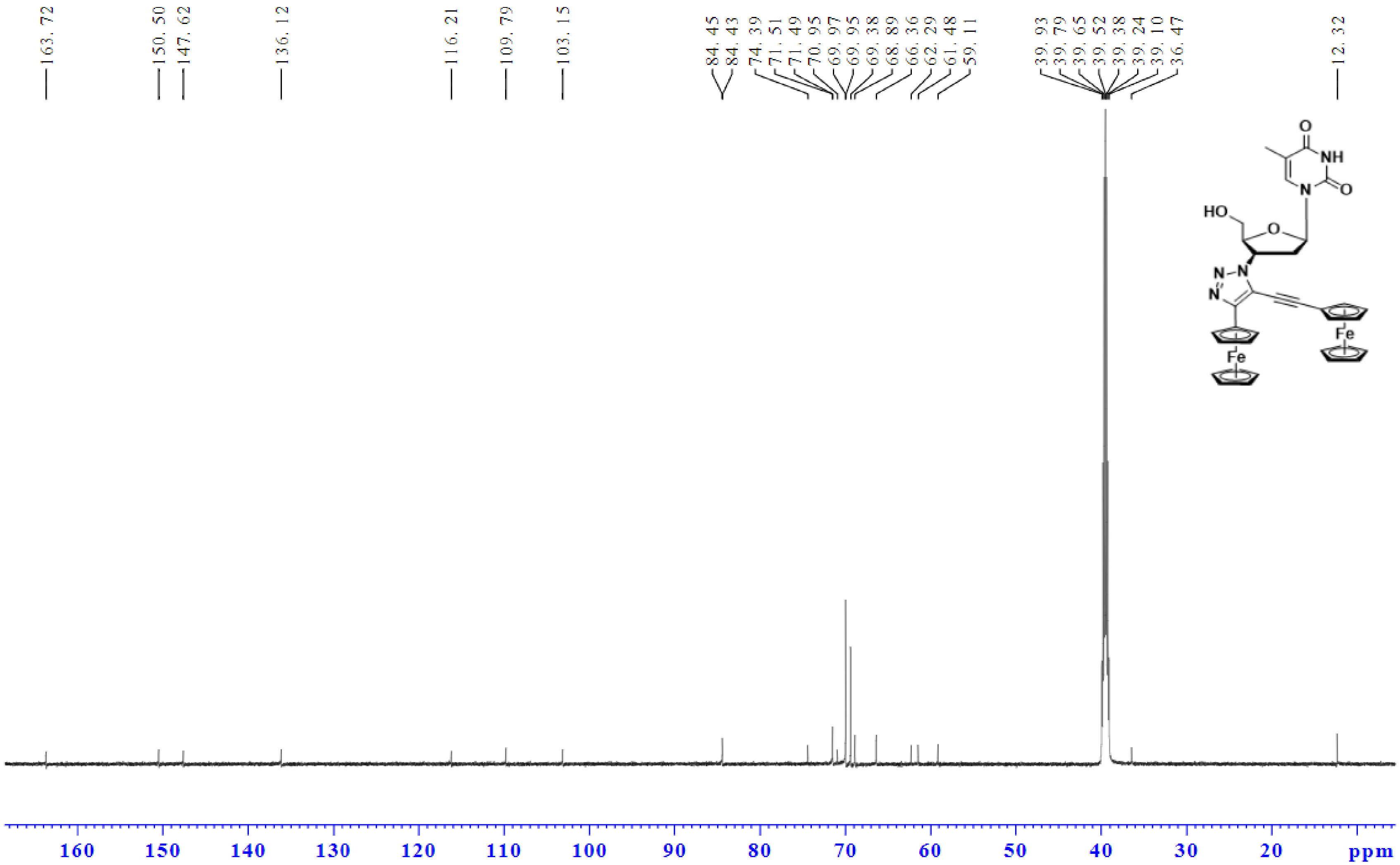

Fig. S7 $^{13}C\{^{1}H\}$-NMR spectrum of **1a** in DMSO-d6 (150 MHz)

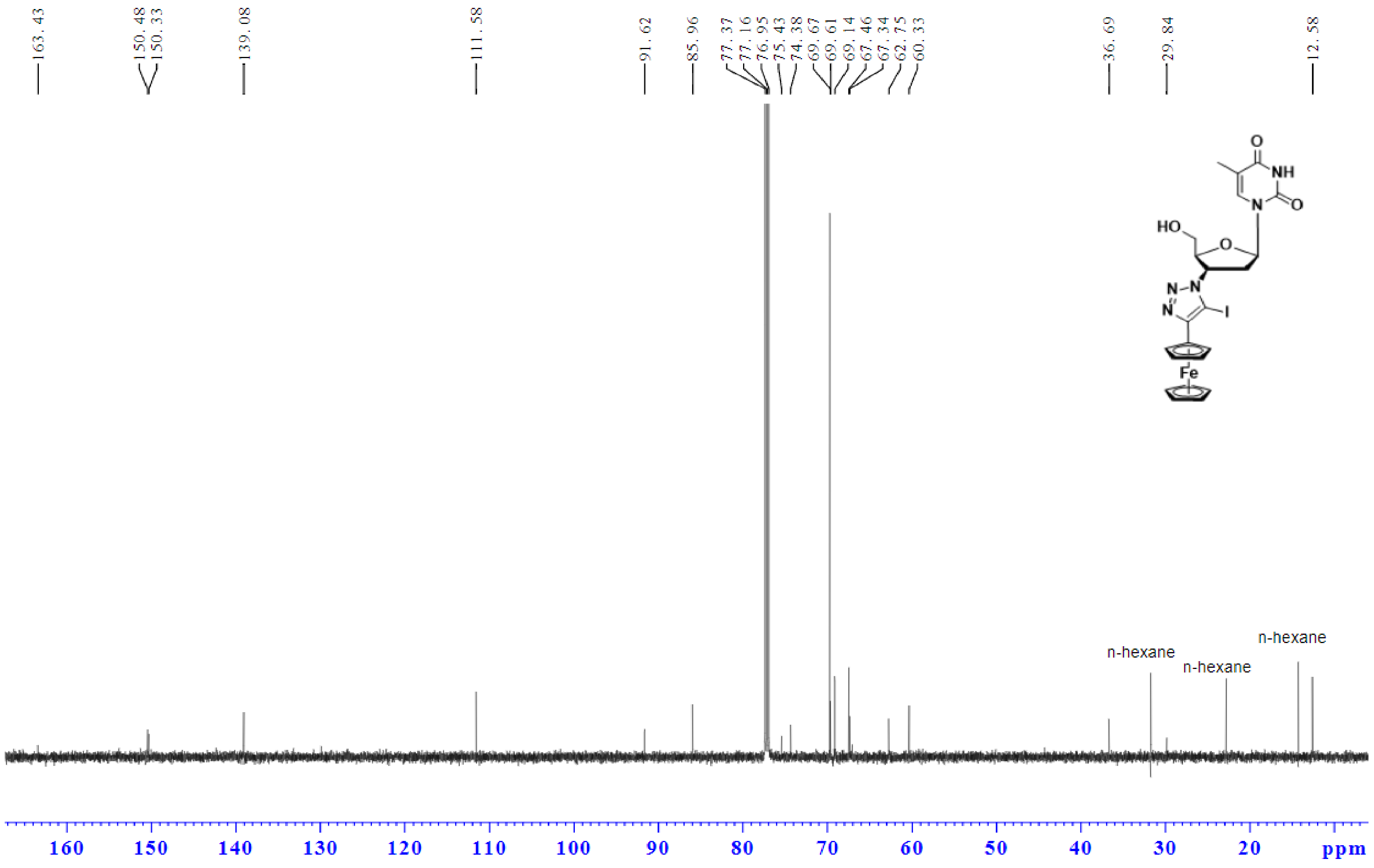

Fig. S8 $^{13}C\{^{1}H\}$-NMR spectrum of **1b** in $CDCl_3$ (150 MHz)

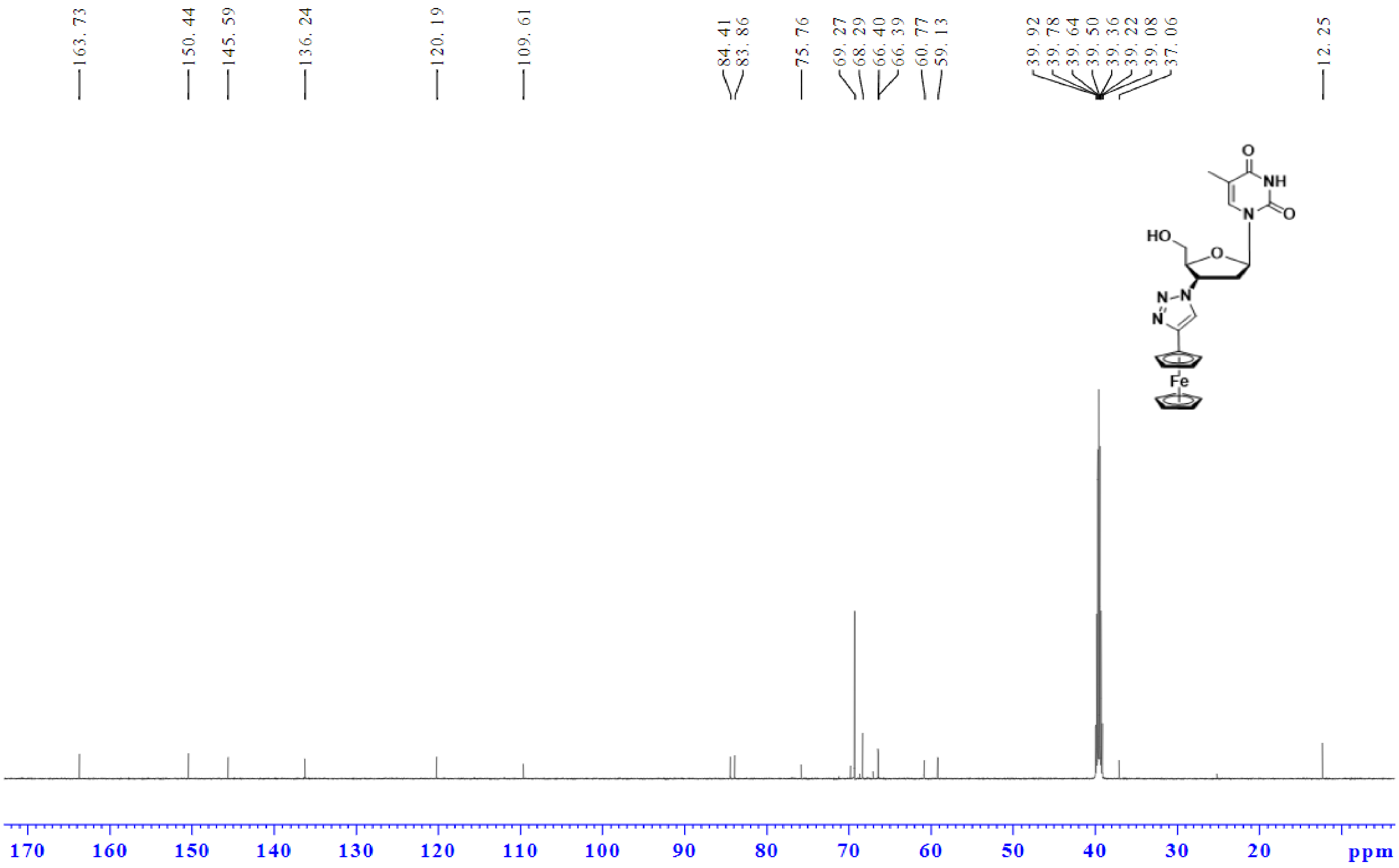

Fig. S9 $^{13}C\{^{1}H\}$-NMR spectrum of **1c** in DMSO-d_6 (150 MHz)

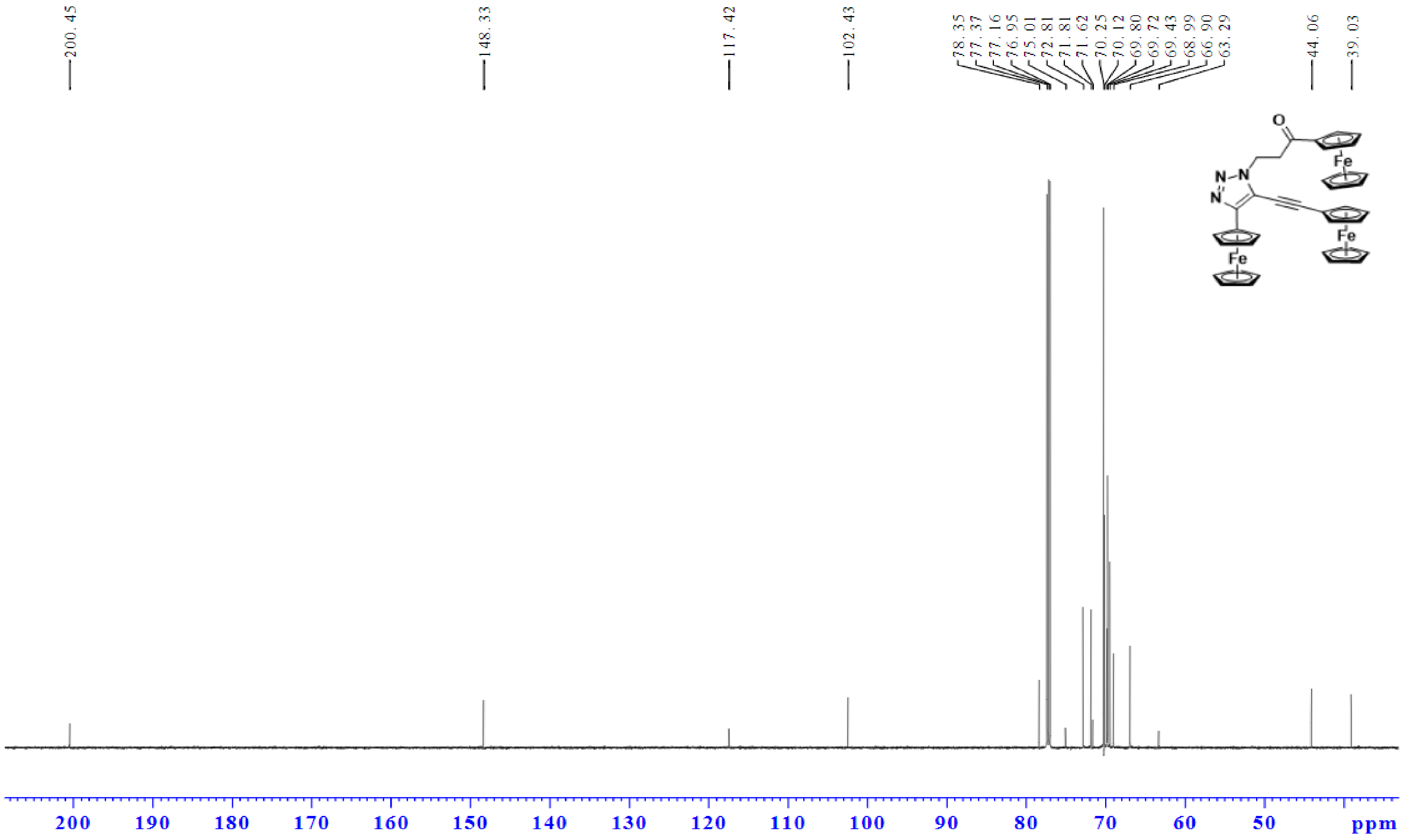

Fig. S10 $^{13}C\{^{1}H\}$-NMR spectrum of **2a** in $CDCl_3$ (150 MHz)

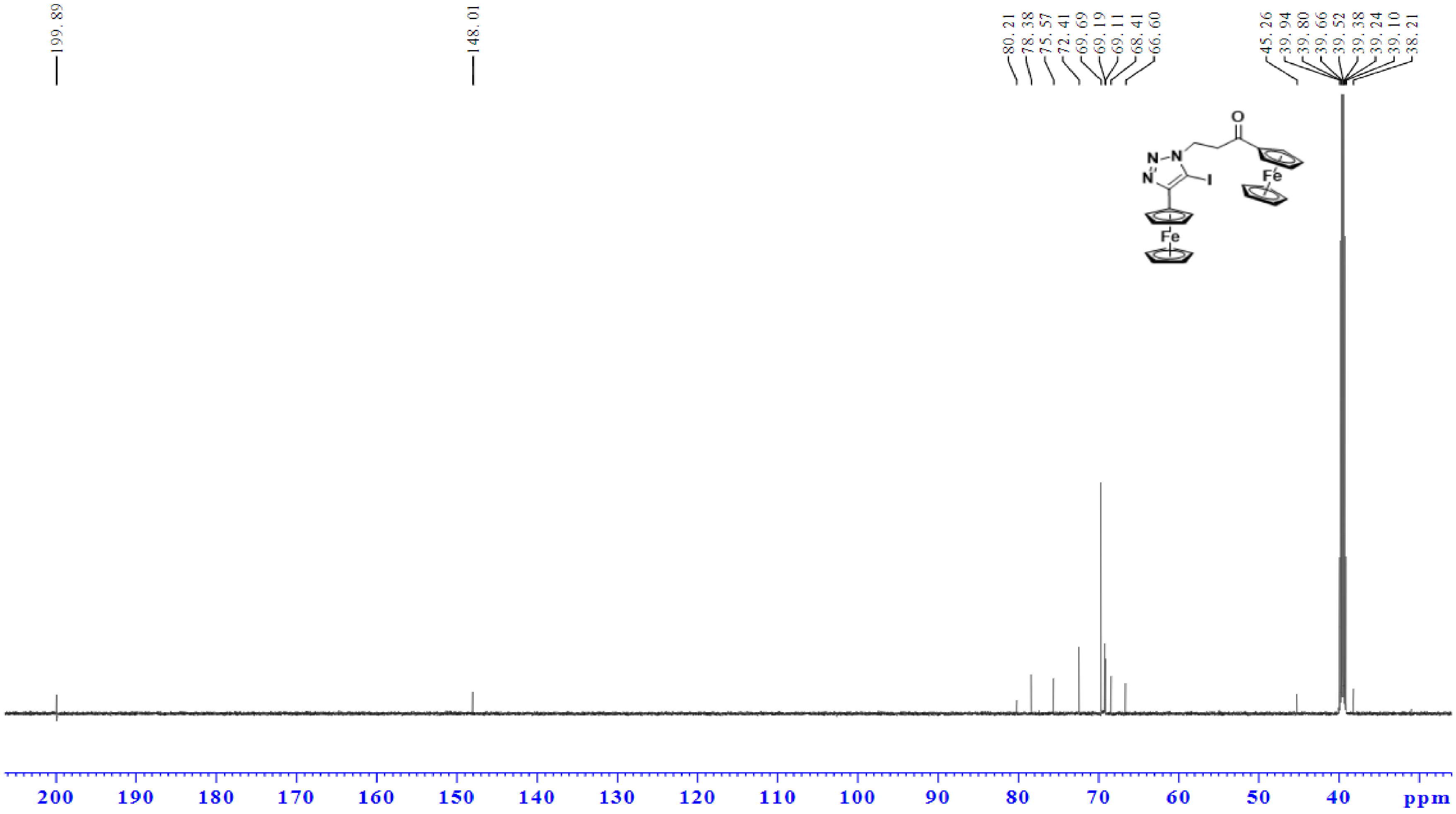

Fig. S11 $^{13}C\{^{1}H\}$-NMR spectrum of **2b** in DMSO-d_6 (150 MHz)

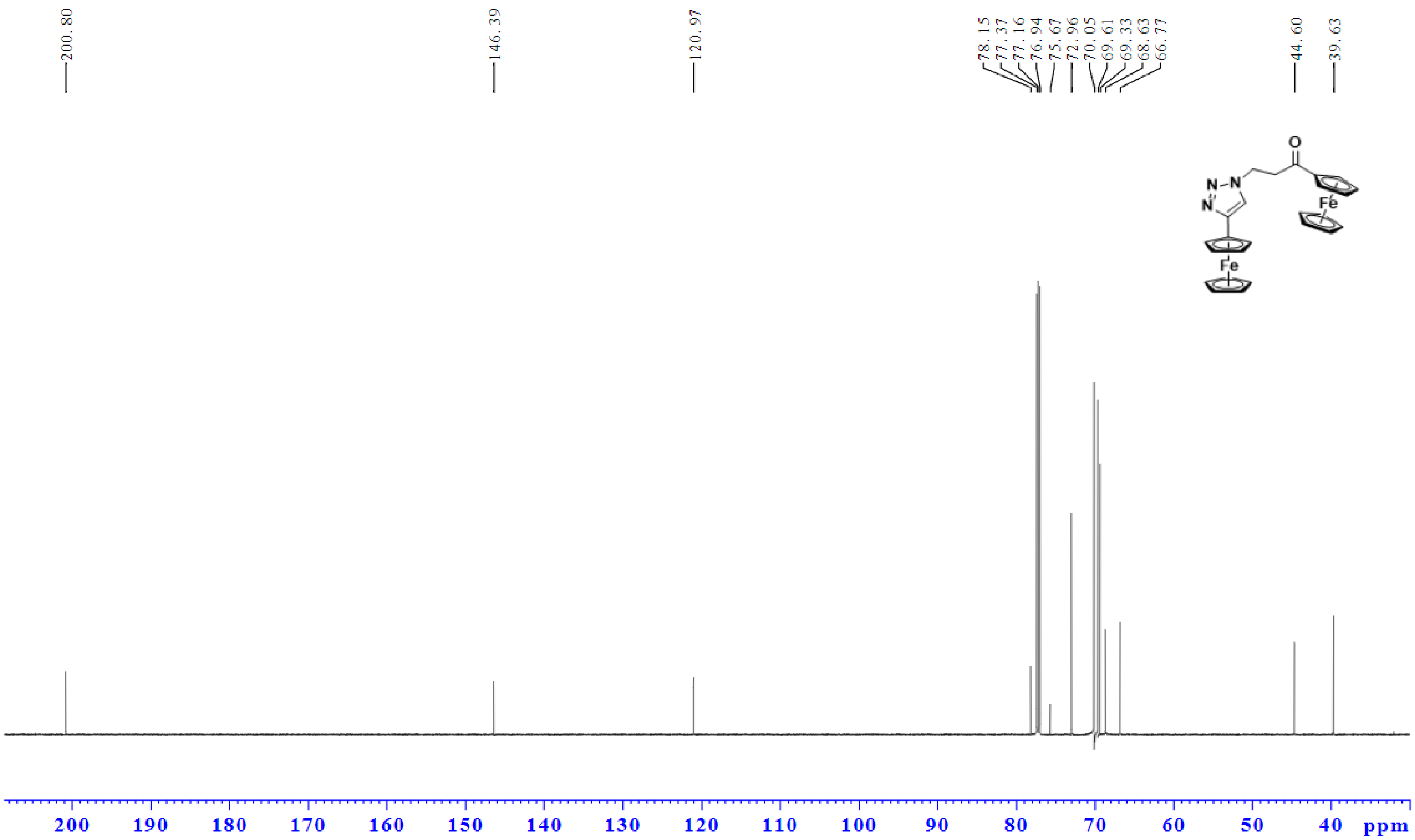

Fig. S12 $^{13}C\{^{1}H\}$-NMR spectrum of **2c** in $CDCl_3$ (150 MHz)

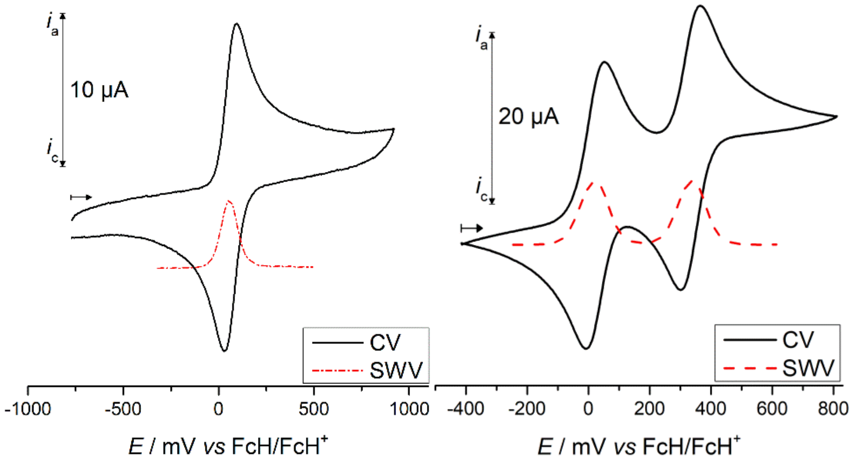

Fig. S13. Cyclic voltammograms of **1c** (left) and **2c** (right) (potential area -750 to 800 mV) as well as square wave voltammograms (dotted lines) (potential area -250 to 600 mV). Conditions: scan rate 100 mV·s^{-1} (CV) and 5 mV·s^{-1} (SWV) in anhydrous dichloromethane solutions (1.0 mmol·L^{1}), supporting electrolyte 0.1 mol·L^{1} of $[NBu_4][B(C_6F_5)_4]$, working electrode glassy carbon.

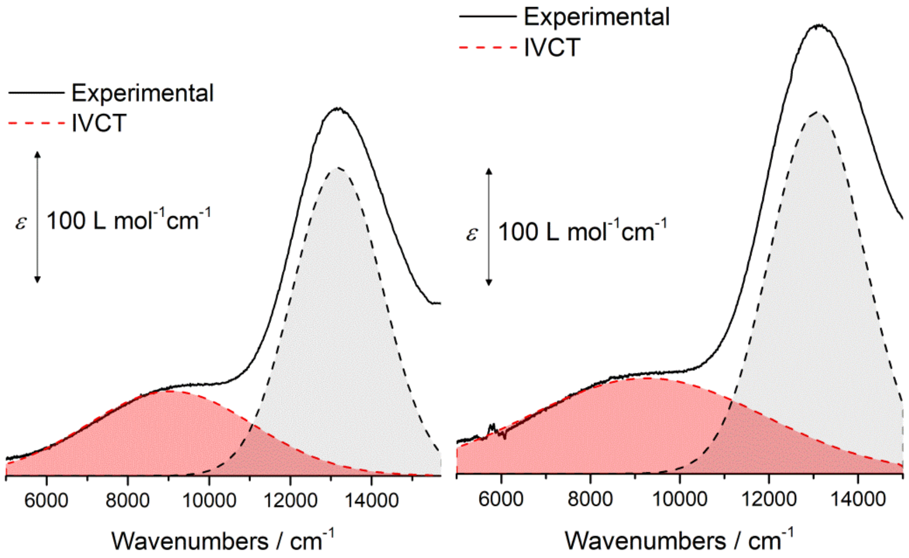

Fig. S14. Deconvolution of the NIR absorptions of [**1a**]$^{+}$ (left) and [**2a**]$^{+}$ (right) by using two gaussian-shaped bands determined by spectroelectrochemistry in an OTTLE cell. Conditions: Anhydrous tetrahydrofuran solutions (5.0 mmol·L^{1}), supporting electrolyte 0.1 mol·L^{1} of $[NBu_4][B(C_6F_5)_4]$.

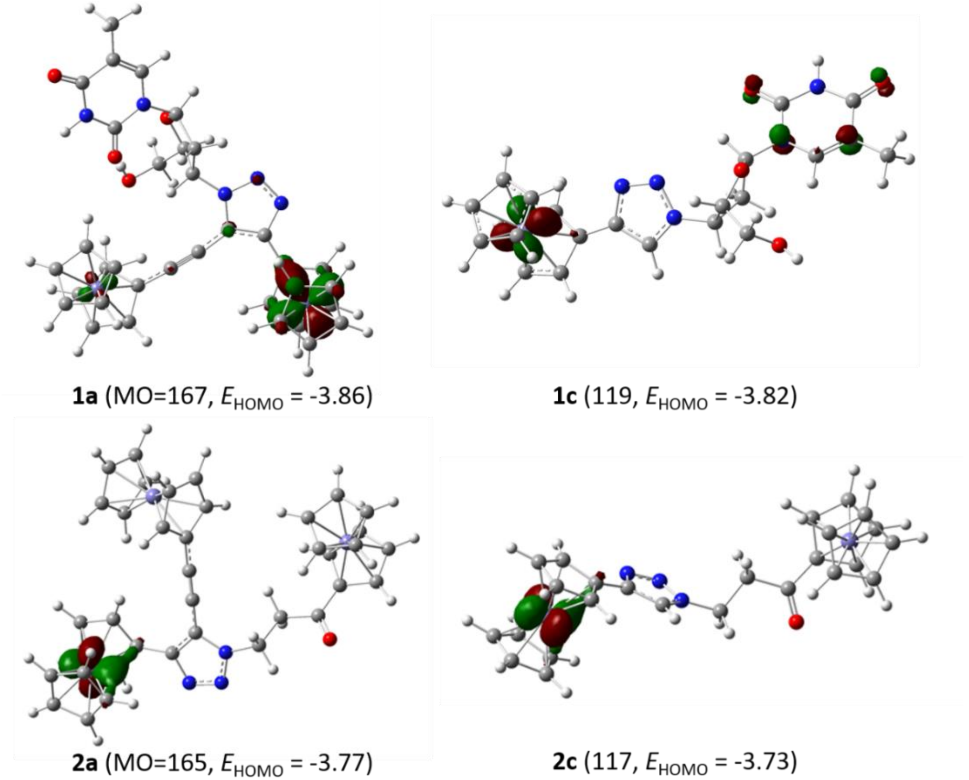

Fig. S15. HOMO orbitals in closed-shell species **1a**, **1c**, **2a**, and **2c** calculated at the BLYP/6-31+G(d)/LanL2DZ level of theory. Orbital energies are in eV. Atomic radii scaled by 50%.

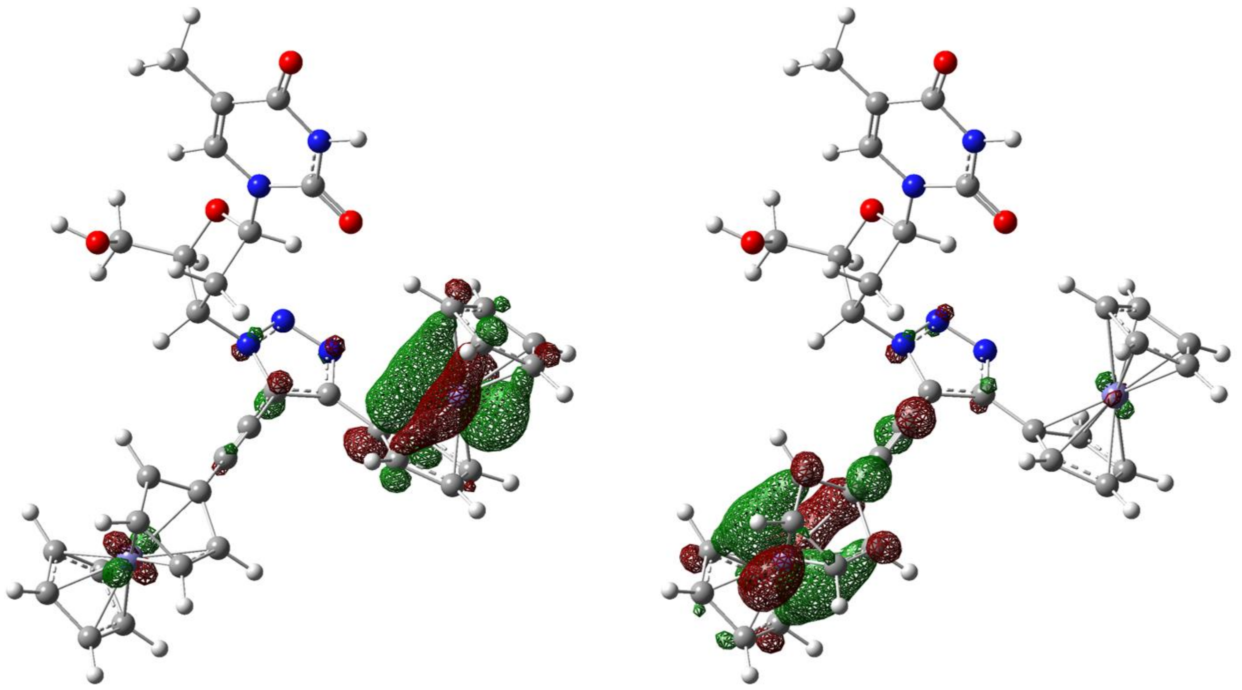

Fig. S16. SOMO (left) and SOMO-1 (right) orbitals in dicationic species [**1a**]$^{2+}$ (triplet state) calculated at the BLYP/6-31+G(d)/LanL2DZ level of theory.

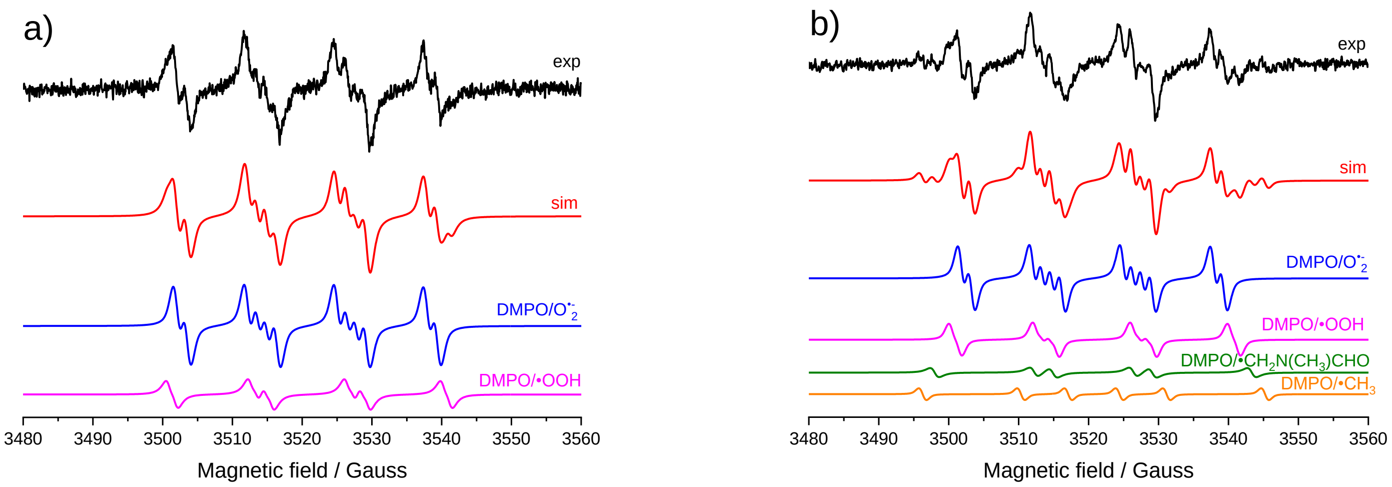

Fig. S17. Experimental, simulated and deconvoluted EPR spectra of DMPO-adducts measured in the DMF solution of (a) **1a** and (b) **2a** under air conditions.

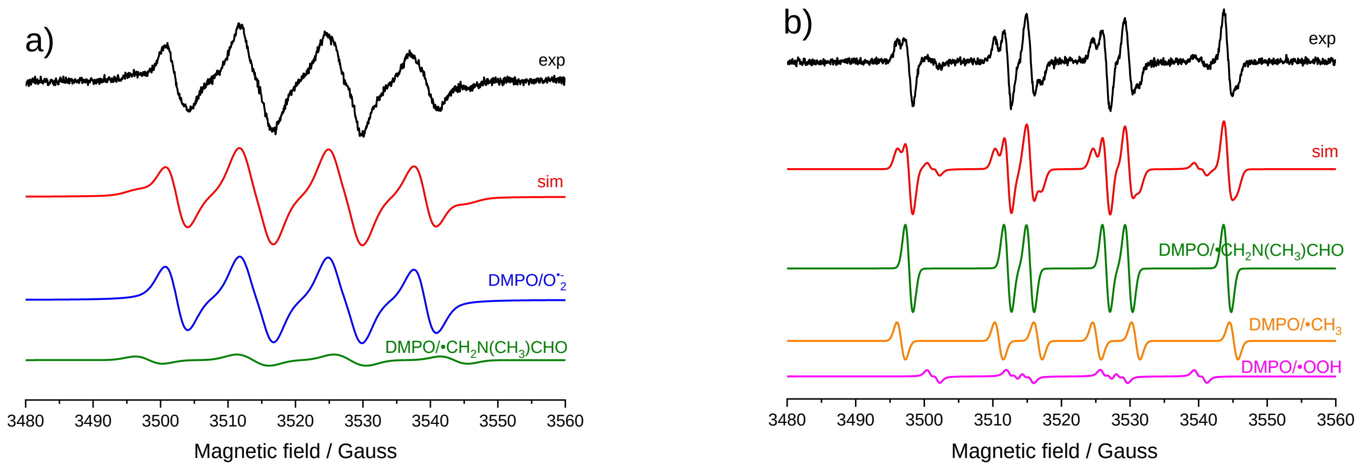

Fig. S18 Experimental, simulated and deconvoluted EPR spectra of DMPO-adducts measured in the DMF solution of **1a** under (a) O_2- and (b) N_2- saturated conditions.

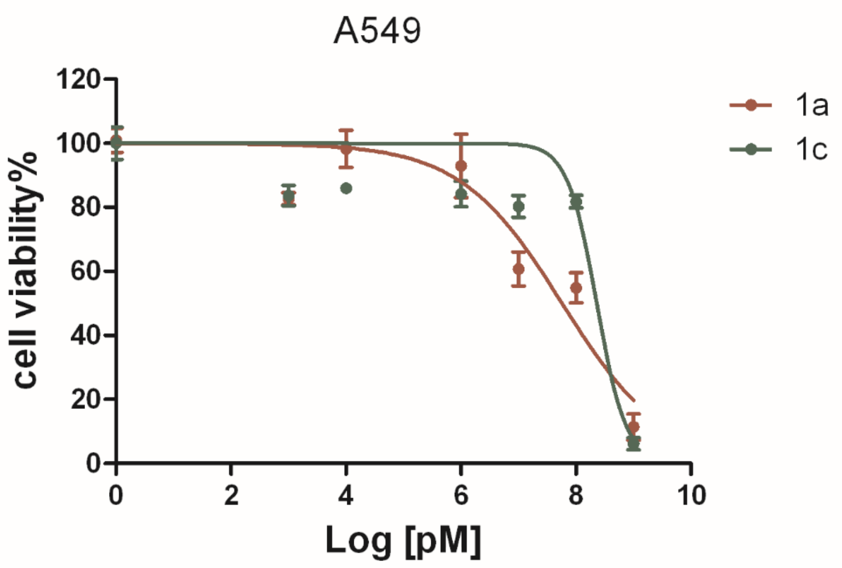

Fig. S19 Cell survival curves of A549 cells treated with **1a** and **1c**

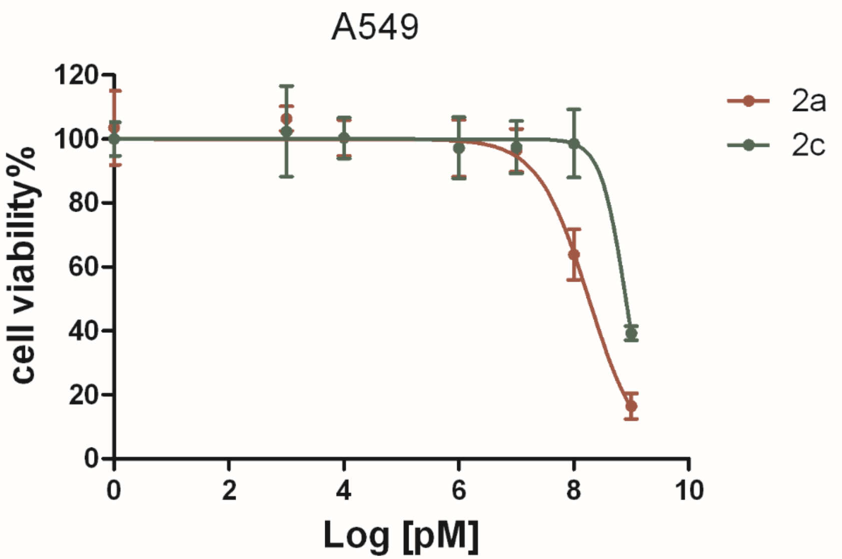

Fig. S20 Cell survival curves of A549 cells treated with **2a** and **2c**

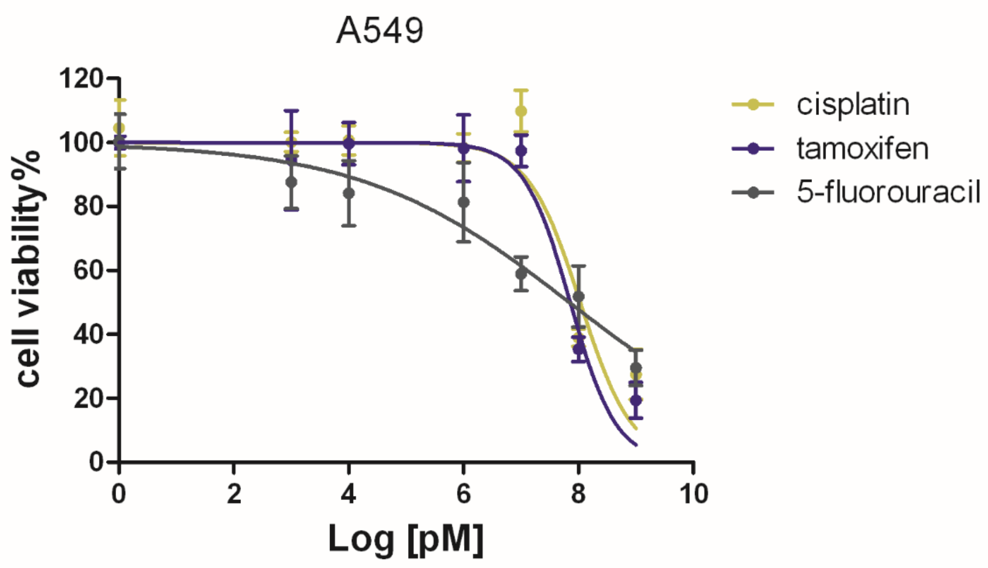

Fig. S21 Cell survival curves of A549 cells treated with cisplatin, tamoxifen and 5-fluorouracil

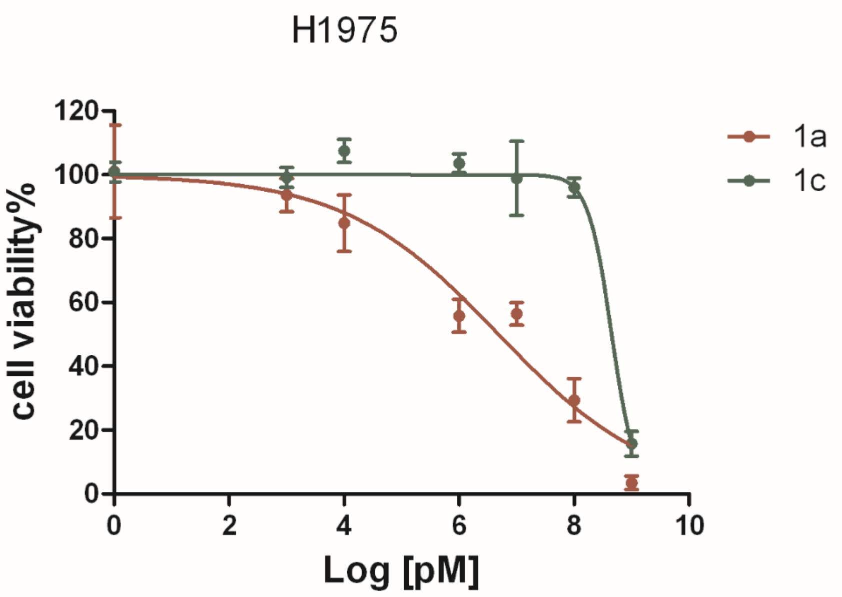

Fig. S22 Cell survival curves of H1975 cells treated with **1a** and **1c**

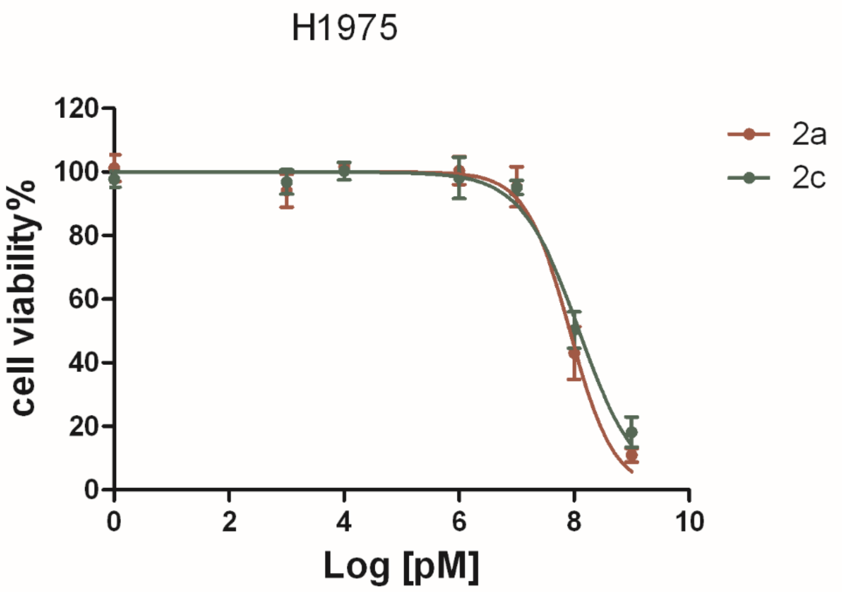

Fig. S23 Cell survival curves of H1975 cells treated with **2a** and **2c**

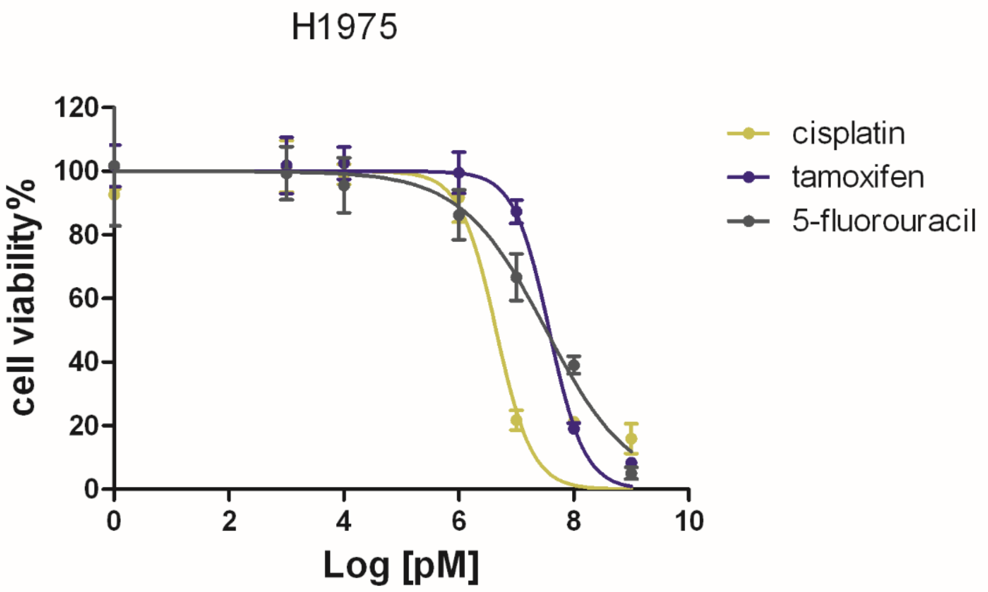

Fig. S24 Cell survival curves of H1975 cells treated with cisplatin, tamoxifen and 5-fluorouracil

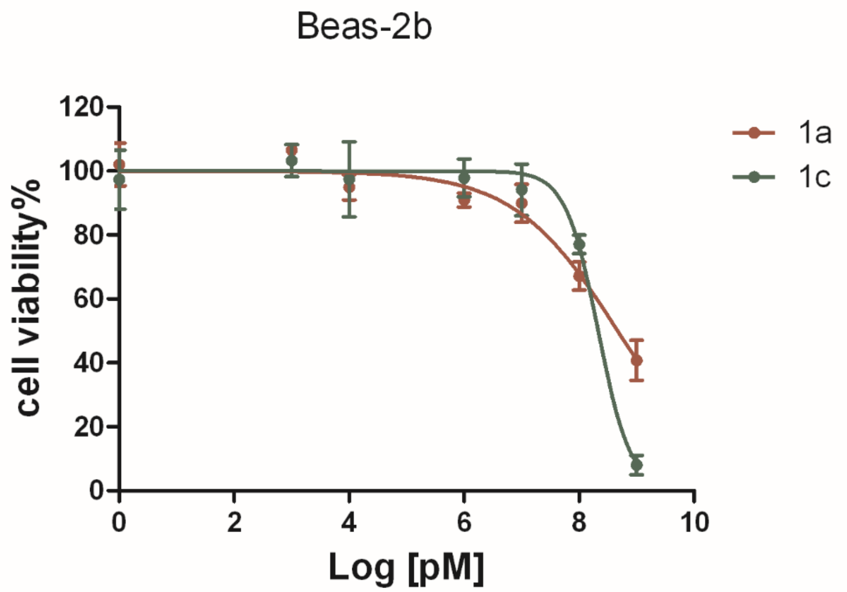

Fig. S25 Cell survival curves of Beas-2b cells treated with **1a** and **1c**

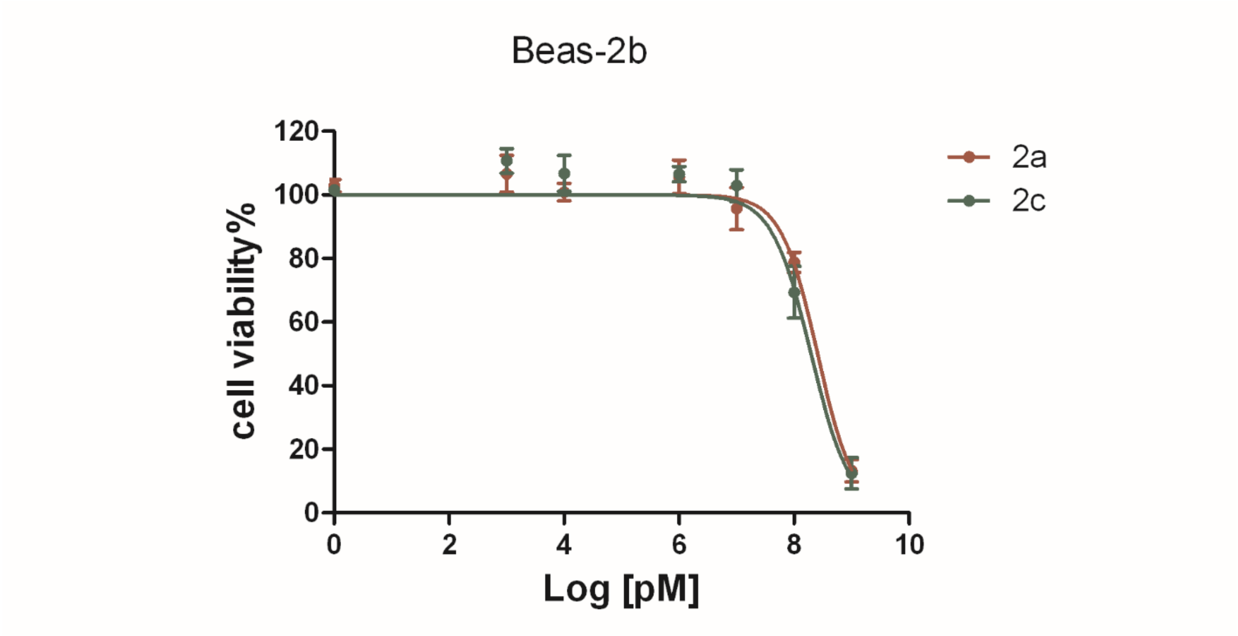

Fig. S26 Cell survival curves of Beas-2b cells treated with **2a** and **2c**

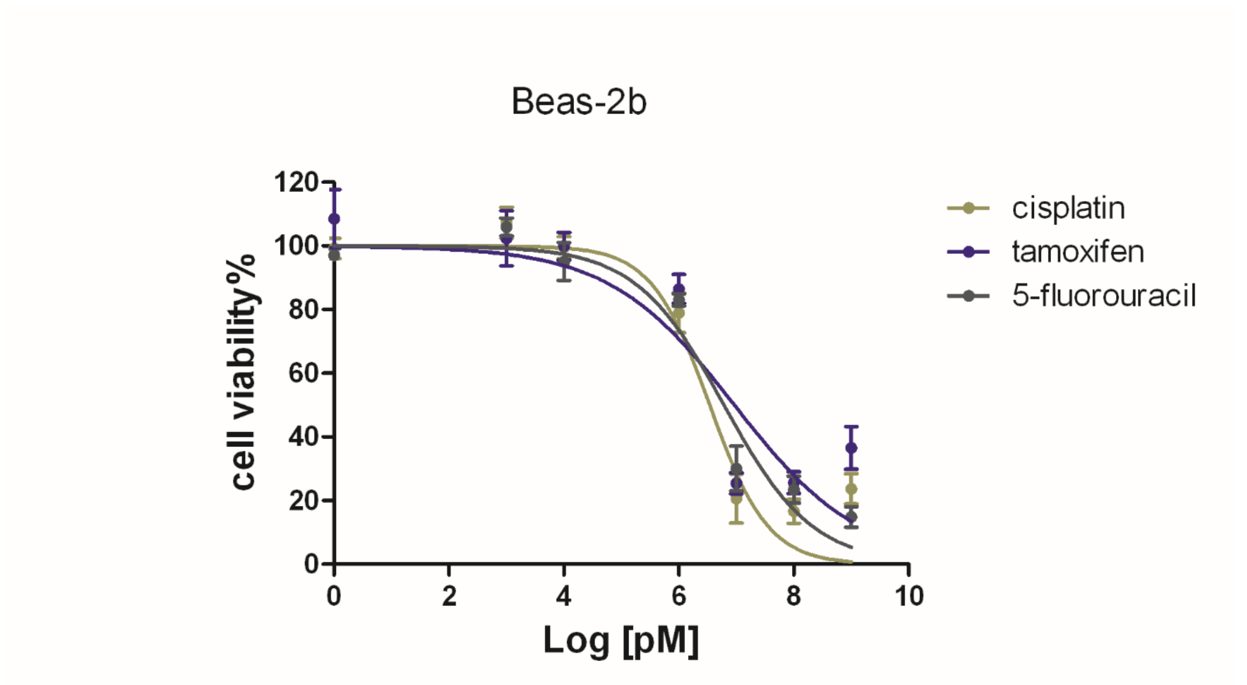

Fig. S27 Cell survival curves of Beas-2b cells treated with cisplatin, tamoxifen and 5-fluorouracil

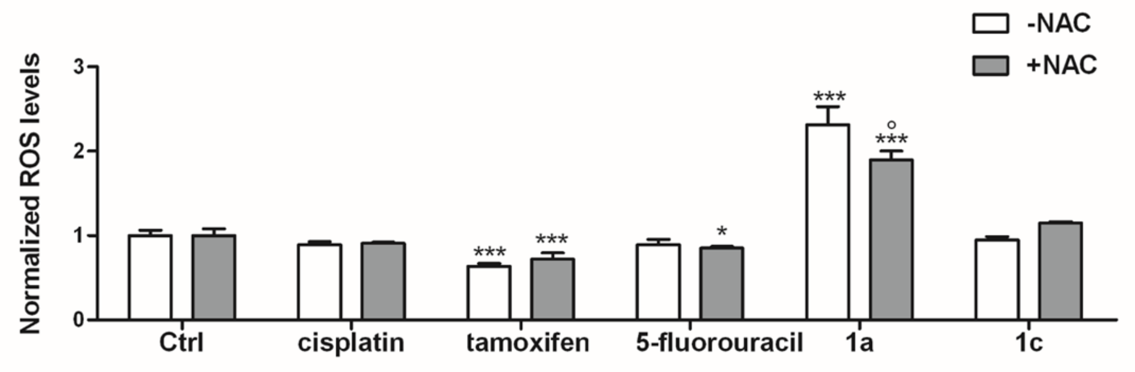

Fig. S28 The effect of 50 μM NAC on relative ROS amount generation by 20 μM of compounds **1a**, **1c**, and reference drugs in A549 cells. The ROS levels were measured by a fluorimetric assay in duplicates. Data are means ± SD (n = 3). *p < 0.05, **p < 0.01, ***p < 0.001: drugs treated cells *vs*. respective untreated (Ctrl) cells; ◦p < 0.05, ◦◦p < 0.01, ◦◦◦p < 0.001:compounds treated cells *vs*. compounds +NAC treated cells.

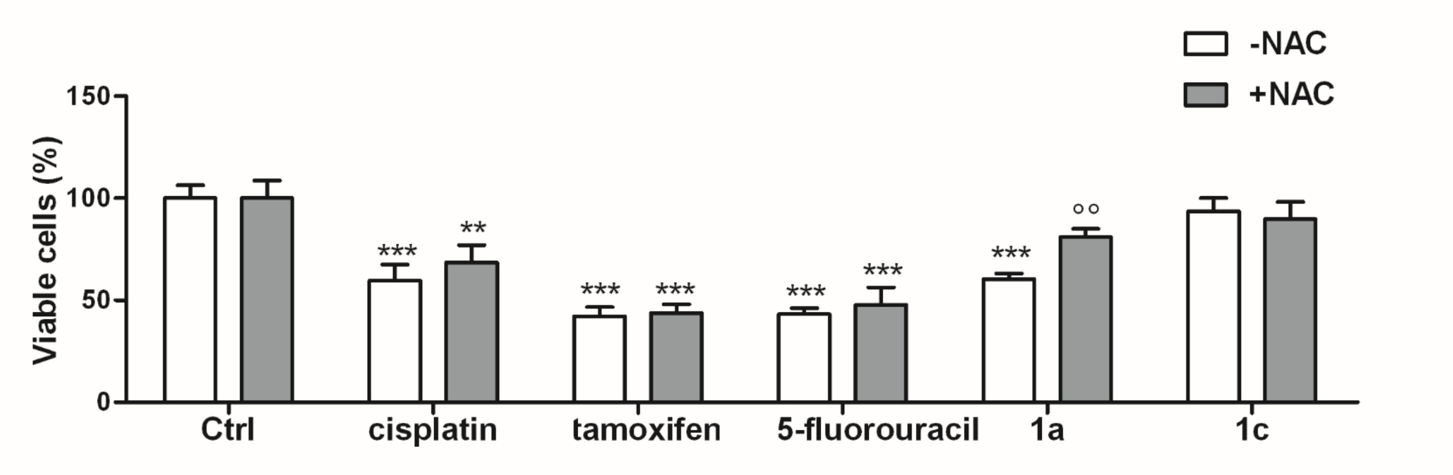

Fig. S29 Viability of A549 cells after 72 h treatment time with 20 μM of compound **1a**, **1c**, and reference drugs, in the presence or absence of 50 μM of the NAC. Cell viability was measured spectrophotometrically in triplicates. Data are means ± SD (n = 3). $^{**}p < 0.01$, $^{***}p < 0.001$: compounds treated cells *vs*. respective untreated (Ctrl) cells; $^{\circ}p < 0.05$: compounds treated cells *vs*. compounds+NAC treated cells.

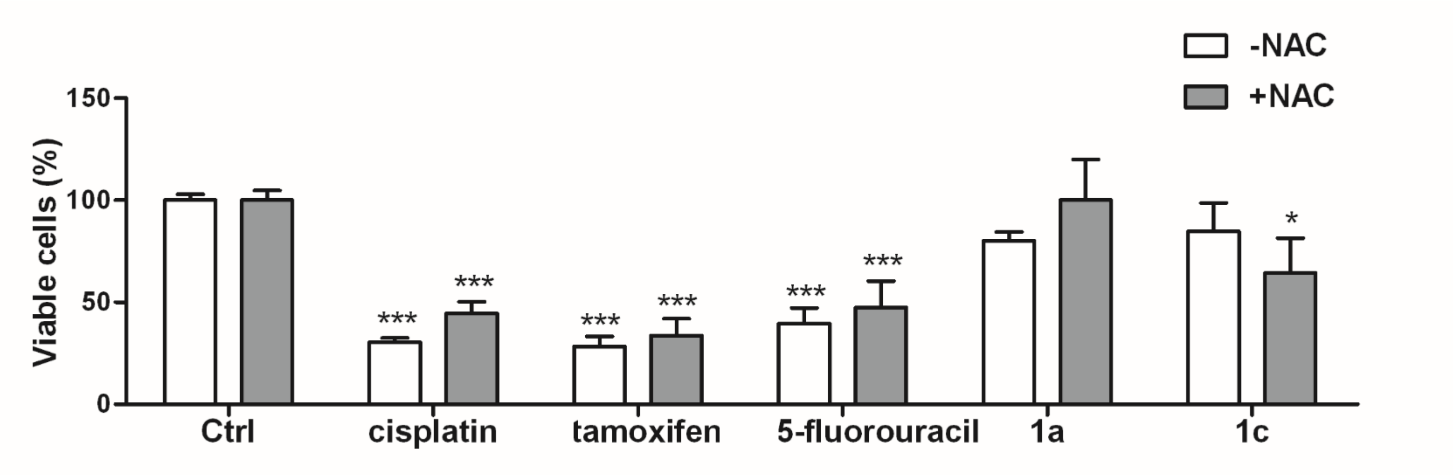

Fig. S30 Viability of BEAS-2B cells after 72 h treatment time with 20 μM of compound **1a**, **1c**, and reference drugs, in the presence or absence of 50 μM of the NAC**.** Cell viability was measured spectrophotometrically in triplicates. Data are means ± SD (n = 3). $^{**}p < 0.01$, $^{***}p < 0.001$: compounds treated cells *vs*. respective untreated (Ctrl) cells; $^{\circ}p < 0.05$: compounds treated cells *vs*. compounds+NAC treated cells.

Table S1. Crystal data and structure refinement for **1a**, **2a** and **2c**

**Compound**	**1a**	**2a**	**2c**
Empirical formula	$C_{34}H_{31}Fe_2N_5O_4$	$C_{37}H_{31}Fe_3N_3O$	$C_{25}H_{23}Fe_2N_3O$
Formula weight	685.34	701.20	493.16
Temperature/K	100(2)	100(2)	100(2)
Crystal system	monoclinic	monoclinic	monoclinic
Space group	$P2_1$	$P2_1$/c	*Cc*
a/Å	13.9050(3)	8.7406(2)	16.5204(4)
b/Å	9.13633(16)	59.6900(13)	5.73208(13)
c/Å	29.0835(5)	11.6165(3)	22.4781(6)
α/°	90	90	90
β/°	97.1162(18)	105.932(3)	107.422(3)
γ/°	90	90	90
Volume/Å^3	3666.34(12)	5827.8(3)	2030.94(9)
Z	4	8	4
ρ_{calc}g/cm^3	1.242	1.598	1.613
μ/mm^{-1}	6.672	12.103	11.637
F(000)	1416.0	2880.0	1016.0
Crystal size/mm^3	0.31 × 0.25 × 0.05	0.08 × 0.08 × 0.03	0.07 × 0.07 × 0.02
Radiation	Cu*K*α (λ = 1.54184)	Cu*K*α (λ = 1.54184)	Cu*K*α (λ = 1.54184)
2Θ range for data collection/°	6.126 to 134.122	5.922 to 134.154	8.246 to 134.036
Index ranges	$-14 \leq h \leq 16$, $-10 \leq k \leq 10$, $-34 \leq l \leq 31$	$-8 \leq h \leq 10$, $-71 \leq k \leq 71$, $-13 \leq l \leq 13$	$-19 \leq h \leq 19$, $-6 \leq k \leq 6$, $-26 \leq l \leq 26$
Reflections collected	25662	41536	12300
Independent reflections	12958 [R_{int} = 0.0676, R_{sigma} = 0.0970]	10416 [R_{int} = 0.0950, R_{sigma} = 0.0777]	3510 [R_{int} = 0.0483, R_{sigma} = 0.0410]
Data/restraints/parameters	12958/85/806	10416/300/793	3510/2/280
Goodness-of-fit on F^2	1.050	1.242	1.041
Final *R* indexes [*I*>=2σ (*I*)]	R_1 = 0.0696, wR_2 = 0.1772	R_1 = 0.1358, wR_2 = 0.2842	R_1 = 0.0374, wR_2 = 0.0957
Final *R* indexes [all data]	R_1 = 0.0825, wR_2 = 0.1870	R_1 = 0.1464, wR_2 = 0.2890	R_1 = 0.0389, wR_2 = 0.0969
Largest diff. peak/hole / e Å^{-3}	1.13/-0.58	2.01/-1.71	0.39/-0.30
Flack parameter	0.016(6)	–	-0.020(6)

Table S2. Bond lengths for **1a** [Å].

Molecule A		Molecule B	
C1'A—C2'A	1.504 (14)	C1'B—C2'B	1.536 (13)
C1'A—N4A	1.463 (12)	C1'B—N4B	1.464 (11)
C1'A—O1A	1.416 (11)	C1'B—O1B	1.403 (11)
C1A—C2A	1.382 (12)	C1B—C2B	1.382 (12)
C1A—C8A	1.456 (12)	C1B—C8B	1.485 (13)
C1A—N3A	1.362 (11)	C1B—N3B	1.370 (11)
C2'A—C3'A	1.546 (12)	C2'B—C3'B	1.545 (12)
C2A—C18A	1.404 (12)	C2B—C18B	1.423 (11)
C2A—N1A	1.351 (11)	C2B—N1B	1.360 (10)
C3'A—C4'A	1.549 (12)	C3'B—C4'B	1.535 (12)
C3'A—N1A	1.471 (10)	C3'B—N1B	1.477 (11)
C3A—N4A	1.343 (13)	C3B—N4B	1.401 (12)
C3A—N5A	1.363 (12)	C3B—N5B	1.365 (12)
C3A—O3A	1.232 (12)	C3B—O3B	1.223 (12)
C4'A—C5'A	1.511 (13)	C4'B—C5'B	1.505 (12)
C4'A—O1A	1.427 (11)	C4'B—O1B	1.448 (10)
C4A—C5A	1.475 (14)	C4B—C5B	1.454 (14)
C4A—N5A	1.380 (13)	C4B—N5B	1.385 (13)
C4A—O4A	1.202 (13)	C4B—O4B	1.233 (12)
C5'A—O2A	1.432 (12)	C5'B—O2B	1.411 (13)
C5A—C6A	1.331 (15)	C5B—C6B	1.323 (15)
C5A—C7A	1.486 (17)	C5B—C7B	1.493 (16)
C6A—N4A	1.389 (14)	C6B—N4B	1.368 (13)
C8A—C9A	1.440 (11)	C8B—C9B	1.429 (12)
C8A—C12A	1.423 (12)	C8B—C12B	1.418 (12)
C8A—Fe1A	2.061 (8)	C8B—Fe1B	2.024 (8)
C9A—C10A	1.422 (12)	C9B—C10B	1.420 (13)
C9A—Fe1A	2.056 (8)	C9B—Fe1B	2.024 (9)
C10A—C11A	1.437 (13)	C10B—C11B	1.433 (14)
C10A—Fe1A	2.055 (8)	C10B—Fe1B	2.016 (9)
C11A—C12A	1.416 (12)	C11B—C12B	1.441 (14)
C11A—Fe1A	2.077 (9)	C11B—Fe1B	2.051 (9)
C12A—Fe1A	2.050 (9)	C12B—Fe1B	2.045 (9)
C13A—C14A	1.408 (14)	C13B—C14B	1.393 (16)

C13A—C17A	1.405 (13)	C13B—C17B	1.428 (15)
C13A—Fe1A	2.027 (9)	C13B—Fe1B	2.053 (9)
C14A—C15A	1.431 (15)	C14B—C15B	1.430 (13)
C14A—Fe1A	2.021 (10)	C14B—Fe1B	2.070 (10)
C15A—C16A	1.414 (14)	C15B—C16B	1.428 (14)
C15A—Fe1A	2.045 (10)	C15B—Fe1B	2.091 (9)
C16A—C17A	1.442 (14)	C16B—C17B	1.436 (13)
C16A—Fe1A	2.045 (9)	C16B—Fe1B	2.082 (8)
C17A—Fe1A	2.025 (9)	C17B—Fe1B	2.057 (9)
C18A—C19A	1.214 (15)	C18B—C19B	1.175 (12)
C19A—C20A	1.468 (12)	C19B—C20B	1.423 (11)
C20A—C21A	1.4200	C20B—C21B	1.452 (11)
C20A—C24A	1.4200	C20B—C24B	1.446 (12)
C20A—Fe2A	1.999 (8)	C20B—Fe2B	2.083 (7)
C21A—C22A	1.4200	C21B—C22B	1.399 (13)
C21A—Fe2A	1.971 (9)	C21B—Fe2B	2.036 (9)
C22A—C23A	1.4200	C22B—C23B	1.427 (14)
C22A—Fe2A	2.041 (9)	C22B—Fe2B	2.057 (8)
C23A—C24A	1.4200	C23B—C24B	1.396 (12)
C23A—Fe2A	2.110 (10)	C23B—Fe2B	2.071 (9)
C24A—Fe2A	2.084 (9)	C24B—Fe2B	2.056 (8)
C25A—C26A	1.403 (15)	C25B—C26B	1.443 (13)
C25A—C29A	1.433 (16)	C25B—C29B	1.408 (13)
C25A—Fe2A	2.045 (9)	C25B—Fe2B	2.046 (9)
C26A—C27A	1.419 (15)	C26B—C27B	1.407 (14)
C26A—Fe2A	2.026 (11)	C26B—Fe2B	2.083 (9)
C27A—C28A	1.386 (15)	C27B—C28B	1.401 (14)
C27A—Fe2A	2.060 (10)	C27B—Fe2B	2.035 (9)
C28A—C29A	1.415 (15)	C28B—C29B	1.429 (14)
C28A—Fe2A	2.031 (10)	C28B—Fe2B	2.001 (8)
C29A—Fe2A	2.006 (12)	C29B—Fe2B	2.034 (9)
N1A—N2A	1.331 (11)	N1B—N2B	1.335 (10)
N2A—N3A	1.321 (10)	N2B—N3B	1.301 (11)

Table S3. Values of valence angles for **1a** [°].

Molecule A		Molecule B	
N4A—C1'A—C2'A	114.9 (9)	N4B—C1'B—C2'B	112.3 (8)
O1A—C1'A—C2'A	107.2 (7)	O1B—C1'B—C2'B	105.8 (7)
O1A—C1'A—N4A	108.6 (8)	O1B—C1'B—N4B	109.0 (7)
C2A—C1A—C8A	130.6 (8)	C2B—C1B—C8B	130.2 (7)
N3A—C1A—C2A	107.3 (7)	N3B—C1B—C2B	109.1 (7)
N3A—C1A—C8A	122.1 (7)	N3B—C1B—C8B	120.6 (7)
C1'A—C2'A—C3'A	104.3 (8)	C1'B—C2'B—C3'B	101.7 (7)
C1A—C2A—C18A	131.8 (8)	C1B—C2B—C18B	132.9 (7)
N1A—C2A—C1A	104.9 (7)	N1B—C2B—C1B	103.1 (7)
N1A—C2A—C18A	123.1 (8)	N1B—C2B—C18B	123.8 (7)
C2'A—C3'A—C4'A	102.2 (7)	C4'B—C3'B—C2'B	103.0 (7)
N1A—C3'A—C2'A	111.0 (7)	N1B—C3'B—C2'B	110.6 (7)
N1A—C3'A—C4'A	111.2 (7)	N1B—C3'B—C4'B	111.7 (7)
N4A—C3A—N5A	115.4 (9)	N5B—C3B—N4B	114.2 (8)
O3A—C3A—N4A	123.3 (8)	O3B—C3B—N4B	123.0 (8)
O3A—C3A—N5A	121.3 (9)	O3B—C3B—N5B	122.8 (9)
C5'A—C4'A—C3'A	112.7 (8)	C5'B—C4'B—C3'B	113.1 (7)
O1A—C4'A—C3'A	107.0 (7)	O1B—C4'B—C3'B	104.9 (7)
O1A—C4'A—C5'A	110.7 (7)	O1B—C4'B—C5'B	110.6 (7)
N5A—C4A—C5A	113.8 (9)	N5B—C4B—C5B	116.4 (8)
O4A—C4A—C5A	124.7 (10)	O4B—C4B—C5B	124.1 (9)
O4A—C4A—N5A	121.4 (9)	O4B—C4B—N5B	119.5 (9)
O2A—C5'A—C4'A	109.2 (7)	O2B—C5'B—C4'B	110.0 (7)
C4A—C5A—C7A	117.5 (10)	C4B—C5B—C7B	118.5 (9)
C6A—C5A—C4A	117.3 (10)	C6B—C5B—C4B	116.5 (9)
C6A—C5A—C7A	124.9 (11)	C6B—C5B—C7B	125.1 (10)
C5A—C6A—N4A	124.2 (10)	C5B—C6B—N4B	125.5 (10)
C1A—C8A—Fe1A	127.1 (6)	C1B—C8B—Fe1B	126.1 (6)
C9A—C8A—C1A	124.9 (7)	C9B—C8B—C1B	125.7 (8)
C9A—C8A—Fe1A	69.3 (4)	C9B—C8B—Fe1B	69.3 (5)
C12A—C8A—C1A	126.5 (7)	C12B—C8B—C1B	126.1 (8)
C12A—C8A—C9A	108.6 (7)	C12B—C8B—C9B	108.2 (8)
C12A—C8A—Fe1A	69.3 (5)	C12B—C8B—Fe1B	70.4 (5)
C8A—C9A—Fe1A	69.7 (4)	C8B—C9B—Fe1B	69.3 (5)
C10A—C9A—C8A	106.6 (7)	C10B—C9B—C8B	107.7 (8)

C10A—C9A—Fe1A	69.7 (5)	C10B—C9B—Fe1B	69.1 (5)
C9A—C10A—C11A	109.0 (7)	C9B—C10B—C11B	109.0 (8)
C9A—C10A—Fe1A	69.8 (5)	C9B—C10B—Fe1B	69.7 (5)
C11A—C10A—Fe1A	70.5 (5)	C11B—C10B—Fe1B	70.7 (5)
C10A—C11A—Fe1A	68.9 (5)	C10B—C11B—C12B	106.5 (8)
C12A—C11A—C10A	107.4 (8)	C10B—C11B—Fe1B	68.1 (5)
C12A—C11A—Fe1A	68.9 (5)	C12B—C11B—Fe1B	69.2 (5)
C8A—C12A—Fe1A	70.2 (5)	C8B—C12B—C11B	108.6 (8)
C11A—C12A—C8A	108.3 (7)	C8B—C12B—Fe1B	68.8 (5)
C11A—C12A—Fe1A	71.0 (5)	C11B—C12B—Fe1B	69.6 (5)
C14A—C13A—Fe1A	69.4 (6)	C14B—C13B—C17B	108.7 (8)
C17A—C13A—C14A	108.0 (9)	C14B—C13B—Fe1B	70.9 (5)
C17A—C13A—Fe1A	69.6 (5)	C17B—C13B—Fe1B	69.8 (5)
C13A—C14A—C15A	108.9 (8)	C13B—C14B—C15B	109.0 (9)
C13A—C14A—Fe1A	69.9 (5)	C13B—C14B—Fe1B	69.6 (6)
C15A—C14A—Fe1A	70.3 (5)	C15B—C14B—Fe1B	70.7 (5)
C14A—C15A—Fe1A	68.5 (6)	C14B—C15B—Fe1B	69.1 (5)
C16A—C15A—C14A	107.3 (8)	C16B—C15B—C14B	107.2 (8)
C16A—C15A—Fe1A	69.8 (5)	C16B—C15B—Fe1B	69.6 (5)
C15A—C16A—C17A	107.6 (8)	C15B—C16B—C17B	107.9 (8)
C15A—C16A—Fe1A	69.7 (5)	C15B—C16B—Fe1B	70.3 (5)
C17A—C16A—Fe1A	68.5 (5)	C17B—C16B—Fe1B	68.8 (5)
C13A—C17A—C16A	108.3 (8)	C13B—C17B—C16B	107.2 (9)
C13A—C17A—Fe1A	69.8 (5)	C13B—C17B—Fe1B	69.5 (5)
C16A—C17A—Fe1A	70.0 (5)	C16B—C17B—Fe1B	70.6 (5)
C19A—C18A—C2A	175.9 (11)	C19B—C18B—C2B	175.6 (8)
C18A—C19A—C20A	173.4 (11)	C18B—C19B—C20B	176.6 (9)
C19A—C20A—Fe2A	127.2 (7)	C19B—C20B—C21B	127.1 (8)
C21A—C20A—C19A	124.1 (8)	C19B—C20B—C24B	127.0 (7)
C21A—C20A—C24A	108.0	C19B—C20B—Fe2B	125.1 (6)
C21A—C20A—Fe2A	68.0 (4)	C21B—C20B—Fe2B	67.6 (5)
C24A—C20A—C19A	127.8 (8)	C24B—C20B—C21B	105.8 (7)
C24A—C20A—Fe2A	72.9 (4)	C24B—C20B—Fe2B	68.6 (4)
C20A—C21A—Fe2A	70.1 (3)	C20B—C21B—Fe2B	71.1 (5)
C22A—C21A—C20A	108.0	C22B—C21B—C20B	108.3 (8)
C22A—C21A—Fe2A	71.9 (4)	C22B—C21B—Fe2B	70.8 (5)
C21A—C22A—C23A	108.0	C21B—C22B—C23B	108.8 (7)
C21A—C22A—Fe2A	66.7 (4)	C21B—C22B—Fe2B	69.2 (5)

C23A—C22A—Fe2A	72.6 (4)	C23B—C22B—Fe2B	70.3 (5)
C22A—C23A—Fe2A	67.4 (4)	C22B—C23B—Fe2B	69.3 (5)
C24A—C23A—C22A	108.0	C24B—C23B—C22B	108.1 (8)
C24A—C23A—Fe2A	69.3 (3)	C24B—C23B—Fe2B	69.6 (5)
C20A—C24A—Fe2A	66.4 (4)	C20B—C24B—Fe2B	70.5 (4)
C23A—C24A—C20A	108.0	C23B—C24B—C20B	109.0 (8)
C23A—C24A—Fe2A	71.2 (3)	C23B—C24B—Fe2B	70.8 (5)
C26A—C25A—C29A	105.9 (9)	C26B—C25B—Fe2B	70.9 (5)
C26A—C25A—Fe2A	69.1 (6)	C29B—C25B—C26B	108.2 (8)
C29A—C25A—Fe2A	67.8 (7)	C29B—C25B—Fe2B	69.4 (5)
C25A—C26A—C27A	110.5 (10)	C25B—C26B—Fe2B	68.2 (5)
C25A—C26A—Fe2A	70.6 (6)	C27B—C26B—C25B	107.1 (8)
C27A—C26A—Fe2A	71.0 (6)	C27B—C26B—Fe2B	68.2 (5)
C26A—C27A—Fe2A	68.4 (6)	C26B—C27B—Fe2B	71.9 (5)
C28A—C27A—C26A	106.3 (9)	C28B—C27B—C26B	108.9 (8)
C28A—C27A—Fe2A	69.1 (6)	C28B—C27B—Fe2B	68.4 (5)
C27A—C28A—C29A	109.8 (10)	C27B—C28B—C29B	108.5 (8)
C27A—C28A—Fe2A	71.3 (6)	C27B—C28B—Fe2B	71.0 (5)
C29A—C28A—Fe2A	68.5 (6)	C29B—C28B—Fe2B	70.5 (5)
C25A—C29A—Fe2A	70.8 (6)	C25B—C29B—C28B	107.3 (8)
C28A—C29A—C25A	107.6 (9)	C25B—C29B—Fe2B	70.3 (5)
C28A—C29A—Fe2A	70.4 (7)	C28B—C29B—Fe2B	68.0 (5)
C8A—Fe1A—C11A	67.6 (3)	C8B—Fe1B—C9B	41.3 (4)
C9A—Fe1A—C8A	41.0 (3)	C8B—Fe1B—C11B	69.5 (3)
C9A—Fe1A—C11A	68.6 (3)	C8B—Fe1B—C12B	40.8 (3)
C10A—Fe1A—C8A	67.8 (3)	C8B—Fe1B—C13B	113.2 (4)
C10A—Fe1A—C9A	40.5 (3)	C8B—Fe1B—C14B	109.9 (4)
C10A—Fe1A—C11A	40.7 (4)	C8B—Fe1B—C15B	135.0 (4)
C12A—Fe1A—C8A	40.5 (3)	C8B—Fe1B—C16B	174.7 (4)
C12A—Fe1A—C9A	69.0 (3)	C8B—Fe1B—C17B	143.3 (4)
C12A—Fe1A—C10A	68.2 (4)	C9B—Fe1B—C11B	69.5 (4)
C12A—Fe1A—C11A	40.1 (3)	C9B—Fe1B—C12B	69.0 (4)
C13A—Fe1A—C8A	108.8 (4)	C9B—Fe1B—C13B	109.7 (4)
C13A—Fe1A—C9A	116.5 (4)	C9B—Fe1B—C14B	134.9 (4)
C13A—Fe1A—C10A	149.5 (4)	C9B—Fe1B—C15B	174.8 (4)
C13A—Fe1A—C11A	168.5 (4)	C9B—Fe1B—C16B	143.8 (4)
C13A—Fe1A—C12A	130.1 (4)	C9B—Fe1B—C17B	113.0 (4)
C13A—Fe1A—C15A	69.1 (4)	C10B—Fe1B—C8B	69.4 (4)

C13A—Fe1A—C16A	69.0 (4)	C10B—Fe1B—C9B	41.2 (4)
C14A—Fe1A—C8A	114.9 (4)	C10B—Fe1B—C11B	41.2 (4)
C14A—Fe1A—C9A	147.8 (4)	C10B—Fe1B—C12B	69.1 (4)
C14A—Fe1A—C10A	169.4 (4)	C10B—Fe1B—C13B	135.3 (4)
C14A—Fe1A—C11A	129.6 (4)	C10B—Fe1B—C14B	174.5 (4)
C14A—Fe1A—C12A	106.8 (4)	C10B—Fe1B—C15B	144.0 (4)
C14A—Fe1A—C13A	40.7 (4)	C10B—Fe1B—C16B	113.8 (4)
C14A—Fe1A—C15A	41.2 (4)	C10B—Fe1B—C17B	109.6 (4)
C14A—Fe1A—C16A	68.6 (4)	C11B—Fe1B—C13B	175.4 (4)
C14A—Fe1A—C17A	68.4 (4)	C11B—Fe1B—C14B	144.1 (4)
C15A—Fe1A—C8A	146.5 (3)	C11B—Fe1B—C15B	113.8 (4)
C15A—Fe1A—C9A	170.6 (4)	C11B—Fe1B—C16B	109.9 (3)
C15A—Fe1A—C10A	131.1 (4)	C11B—Fe1B—C17B	135.0 (4)
C15A—Fe1A—C11A	107.4 (4)	C12B—Fe1B—C11B	41.2 (4)
C15A—Fe1A—C12A	113.8 (4)	C12B—Fe1B—C13B	143.2 (4)
C15A—Fe1A—C16A	40.5 (4)	C12B—Fe1B—C14B	114.2 (4)
C16A—Fe1A—C8A	172.3 (3)	C12B—Fe1B—C15B	110.5 (4)
C16A—Fe1A—C9A	132.7 (4)	C12B—Fe1B—C16B	135.5 (4)
C16A—Fe1A—C10A	110.1 (3)	C12B—Fe1B—C17B	175.4 (4)
C16A—Fe1A—C11A	116.0 (4)	C13B—Fe1B—C14B	39.5 (4)
C16A—Fe1A—C12A	146.4 (4)	C13B—Fe1B—C15B	67.3 (4)
C17A—Fe1A—C8A	132.3 (4)	C13B—Fe1B—C16B	67.8 (3)
C17A—Fe1A—C9A	109.9 (4)	C13B—Fe1B—C17B	40.7 (4)
C17A—Fe1A—C10A	118.0 (4)	C14B—Fe1B—C15B	40.2 (4)
C17A—Fe1A—C11A	149.7 (4)	C14B—Fe1B—C16B	67.3 (4)
C17A—Fe1A—C12A	169.8 (4)	C16B—Fe1B—C15B	40.0 (4)
C17A—Fe1A—C13A	40.6 (4)	C17B—Fe1B—C14B	67.5 (4)
C17A—Fe1A—C15A	69.0 (4)	C17B—Fe1B—C15B	67.8 (4)
C17A—Fe1A—C16A	41.5 (4)	C17B—Fe1B—C16B	40.6 (4)
C20A—Fe2A—C22A	69.3 (2)	C20B—Fe2B—C26B	123.8 (3)
C20A—Fe2A—C23A	67.9 (2)	C21B—Fe2B—C20B	41.3 (3)
C20A—Fe2A—C24A	40.63 (16)	C21B—Fe2B—C22B	40.0 (4)
C20A—Fe2A—C25A	110.7 (4)	C21B—Fe2B—C23B	68.0 (4)
C20A—Fe2A—C26A	126.0 (4)	C21B—Fe2B—C24B	68.8 (3)
C20A—Fe2A—C27A	160.0 (4)	C21B—Fe2B—C25B	124.0 (4)
C20A—Fe2A—C28A	160.2 (4)	C21B—Fe2B—C26B	160.6 (4)
C20A—Fe2A—C29A	125.1 (4)	C22B—Fe2B—C20B	67.8 (3)
C21A—Fe2A—C20A	41.91 (17)	C22B—Fe2B—C23B	40.5 (4)

C21A—Fe2A—C22A	41.40 (17)	C22B—Fe2B—C26B	158.0 (4)
C21A—Fe2A—C23A	68.4 (2)	C23B—Fe2B—C20B	67.7 (3)
C21A—Fe2A—C24A	68.95 (19)	C23B—Fe2B—C26B	122.4 (4)
C21A—Fe2A—C25A	132.7 (4)	C24B—Fe2B—C20B	40.9 (3)
C21A—Fe2A—C26A	167.3 (4)	C24B—Fe2B—C22B	67.5 (3)
C21A—Fe2A—C27A	152.1 (4)	C24B—Fe2B—C23B	39.5 (4)
C21A—Fe2A—C28A	123.1 (4)	C24B—Fe2B—C26B	108.0 (4)
C21A—Fe2A—C29A	113.7 (5)	C25B—Fe2B—C20B	108.8 (3)
C22A—Fe2A—C23A	39.97 (16)	C25B—Fe2B—C22B	159.4 (3)
C22A—Fe2A—C24A	67.7 (2)	C25B—Fe2B—C23B	158.9 (4)
C22A—Fe2A—C25A	171.0 (5)	C25B—Fe2B—C24B	124.2 (4)
C22A—Fe2A—C27A	114.5 (4)	C25B—Fe2B—C26B	40.9 (4)
C24A—Fe2A—C23A	39.57 (17)	C27B—Fe2B—C20B	158.3 (4)
C25A—Fe2A—C23A	148.9 (5)	C27B—Fe2B—C21B	158.3 (4)
C25A—Fe2A—C24A	118.6 (4)	C27B—Fe2B—C22B	122.5 (4)
C25A—Fe2A—C27A	68.7 (4)	C27B—Fe2B—C23B	106.8 (4)
C26A—Fe2A—C22A	147.2 (4)	C27B—Fe2B—C24B	121.6 (4)
C26A—Fe2A—C23A	113.5 (5)	C27B—Fe2B—C25B	68.3 (4)
C26A—Fe2A—C24A	104.1 (4)	C27B—Fe2B—C26B	39.9 (4)
C26A—Fe2A—C25A	40.3 (4)	C28B—Fe2B—C20B	160.2 (4)
C26A—Fe2A—C27A	40.6 (4)	C28B—Fe2B—C21B	122.7 (3)
C26A—Fe2A—C28A	67.2 (5)	C28B—Fe2B—C22B	107.2 (4)
C27A—Fe2A—C23A	101.8 (4)	C28B—Fe2B—C23B	121.8 (4)
C27A—Fe2A—C24A	120.8 (4)	C28B—Fe2B—C24B	156.8 (4)
C28A—Fe2A—C22A	108.1 (4)	C28B—Fe2B—C25B	68.7 (4)
C28A—Fe2A—C23A	123.5 (4)	C28B—Fe2B—C26B	68.0 (4)
C28A—Fe2A—C24A	158.1 (4)	C28B—Fe2B—C27B	40.6 (4)
C28A—Fe2A—C25A	68.6 (4)	C28B—Fe2B—C29B	41.5 (4)
C28A—Fe2A—C27A	39.6 (4)	C29B—Fe2B—C20B	123.9 (4)
C29A—Fe2A—C22A	130.7 (5)	C29B—Fe2B—C21B	108.0 (4)
C29A—Fe2A—C23A	163.8 (4)	C29B—Fe2B—C22B	123.3 (4)
C29A—Fe2A—C24A	156.6 (4)	C29B—Fe2B—C23B	159.0 (4)
C29A—Fe2A—C25A	41.4 (5)	C29B—Fe2B—C24B	160.3 (4)
C29A—Fe2A—C26A	68.3 (5)	C29B—Fe2B—C25B	40.4 (4)
C29A—Fe2A—C27A	68.6 (4)	C29B—Fe2B—C26B	68.2 (4)
C29A—Fe2A—C28A	41.0 (4)	C29B—Fe2B—C27B	68.7 (4)
C2A—N1A—C3'A	127.3 (7)	C2B—N1B—C3'B	125.3 (7)
N2A—N1A—C2A	111.1 (7)	N2B—N1B—C2B	111.3 (7)

N2A—N1A—C3'A	121.6 (7)	N2B—N1B—C3'B	123.5 (7)
N3A—N2A—N1A	107.2 (7)	N3B—N2B—N1B	108.5 (7)
N2A—N3A—C1A	109.4 (7)	N2B—N3B—C1B	108.0 (7)
C3A—N4A—C1'A	121.1 (8)	C3B—N4B—C1'B	118.5 (7)
C3A—N4A—C6A	121.0 (8)	C6B—N4B—C1'B	120.8 (8)
C6A—N4A—C1'A	117.9 (8)	C6B—N4B—C3B	120.7 (8)
C3A—N5A—C4A	128.1 (9)	C3B—N5B—C4B	126.7 (8)
C1'A—O1A—C4'A	111.6 (7)	C1'B—O1B—C4'B	112.4 (6)

Table S4. Values of torsion angles for **1a** [°].

Molecule A		Molecule B	
C1'A—C2'A—C3'A—C4'A	−27.0 (9)	C1'B—C2'B—C3'B—C4'B	−34.0 (9)
C1'A—C2'A—C3'A—N1A	91.6 (9)	C1'B—C2'B—C3'B—N1B	85.5 (8)
C1A—C2A—N1A—C3'A	−179.8 (8)	C1B—C2B—N1B—C3'B	−177.8 (7)
C1A—C2A—N1A—N2A	0.8 (10)	C1B—C2B—N1B—N2B	0.8 (9)
C1A—C8A—C9A—C10A	178.2 (8)	C1B—C8B—C9B—C10B	−178.9 (8)
C1A—C8A—C9A—Fe1A	−121.5 (8)	C1B—C8B—C9B—Fe1B	−120.2 (8)
C1A—C8A—C12A—C11A	−177.5 (8)	C1B—C8B—C12B—C11B	179.3 (8)
C1A—C8A—C12A—Fe1A	121.5 (8)	C1B—C8B—C12B—Fe1B	120.9 (8)
C2'A—C1'A—N4A—C3A	109.8 (11)	C2'B—C1'B—N4B—C3B	109.8 (9)
C2'A—C1'A—N4A—C6A	−69.9 (13)	C2'B—C1'B—N4B—C6B	−69.4 (11)
C2'A—C1'A—O1A—C4'A	−13.5 (10)	C2'B—C1'B—O1B—C4'B	−16.0 (10)
C2'A—C3'A—C4'A—C5'A	−102.2 (8)	C2'B—C3'B—C4'B—C5'B	−95.1 (9)
C2'A—C3'A—C4'A—O1A	19.8 (9)	C2'B—C3'B—C4'B—O1B	25.5 (8)
C2'A—C3'A—N1A—C2A	146.7 (9)	C2'B—C3'B—N1B—C2B	171.8 (7)
C2'A—C3'A—N1A—N2A	−33.9 (12)	C2'B—C3'B—N1B—N2B	−6.6 (11)
C2A—C1A—C8A—C9A	−165.5 (8)	C2B—C1B—C8B—C9B	−0.6 (14)
C2A—C1A—C8A—C12A	14.7 (15)	C2B—C1B—C8B—C12B	179.2 (8)
C2A—C1A—C8A—Fe1A	105.2 (10)	C2B—C1B—C8B—Fe1B	−89.7 (10)
C2A—C1A—N3A—N2A	0.1 (10)	C2B—C1B—N3B—N2B	−1.1 (9)
C2A—N1A—N2A—N3A	−0.8 (10)	C2B—N1B—N2B—N3B	−1.5 (9)
C3'A—C4'A—C5'A—O2A	45.3 (10)	C3'B—C4'B—C5'B—O2B	48.4 (10)
C3'A—C4'A—O1A—C1'A	−4.5 (10)	C3'B—C4'B—O1B—C1'B	−6.2 (9)
C3'A—N1A—N2A—N3A	179.8 (8)	C3'B—N1B—N2B—N3B	177.2 (7)
C4'A—C3'A—N1A—C2A	−100.3 (10)	C4'B—C3'B—N1B—C2B	−74.0 (10)
C4'A—C3'A—N1A—N2A	79.1 (10)	C4'B—C3'B—N1B—N2B	107.5 (9)
C4A—C5A—C6A—N4A	2 (2)	C4B—C5B—C6B—N4B	−2 (2)
C5'A—C4'A—O1A—C1'A	118.7 (8)	C5'B—C4'B—O1B—C1'B	116.0 (8)
C5A—C4A—N5A—C3A	4.0 (18)	C5B—C4B—N5B—C3B	−1.4 (17)
C5A—C6A—N4A—C1'A	−177.8 (14)	C5B—C6B—N4B—C1'B	−179.9 (12)
C5A—C6A—N4A—C3A	3 (2)	C5B—C6B—N4B—C3B	0.9 (18)
C7A—C5A—C6A—N4A	176.4 (17)	C7B—C5B—C6B—N4B	178.7 (13)
C8A—C1A—C2A—C18A	0.5 (16)	C8B—C1B—C2B—C18B	−5.8 (15)
C8A—C1A—C2A—N1A	176.5 (8)	C8B—C1B—C2B—N1B	177.7 (8)
C8A—C1A—N3A—N2A	−177.2 (8)	C8B—C1B—N3B—N2B	−178.9 (7)
C8A—C9A—C10A—C11A	0.6 (9)	C8B—C9B—C10B—C11B	−1.2 (10)

C8A—C9A—C10A—Fe1A	60.3 (6)	C8B—C9B—C10B—Fe1B	58.8 (6)
C9A—C8A—C12A—C11A	2.7 (10)	C9B—C8B—C12B—C11B	−0.9 (9)
C9A—C8A—C12A—Fe1A	−58.3 (6)	C9B—C8B—C12B—Fe1B	−59.3 (6)
C9A—C10A—C11A—C12A	1.1 (10)	C9B—C10B—C11B—C12B	0.7 (10)
C9A—C10A—C11A—Fe1A	59.3 (6)	C9B—C10B—C11B—Fe1B	59.4 (6)
C10A—C11A—C12A—C8A	−2.3 (10)	C10B—C11B—C12B—C8B	0.2 (9)
C10A—C11A—C12A—Fe1A	58.2 (6)	C10B—C11B—C12B—Fe1B	58.0 (6)
C12A—C8A—C9A—C10A	−2.0 (9)	C12B—C8B—C9B—C10B	1.3 (10)
C12A—C8A—C9A—Fe1A	58.3 (6)	C12B—C8B—C9B—Fe1B	60.0 (6)
C13A—C14A—C15A—C16A	0.2 (11)	C13B—C14B—C15B—C16B	−0.1 (10)
C13A—C14A—C15A—Fe1A	59.5 (7)	C13B—C14B—C15B—Fe1B	59.3 (7)
C14A—C13A—C17A—C16A	0.7 (10)	C14B—C13B—C17B—C16B	0.4 (10)
C14A—C13A—C17A—Fe1A	−59.0 (6)	C14B—C13B—C17B—Fe1B	−60.5 (6)
C14A—C15A—C16A—C17A	0.2 (10)	C14B—C15B—C16B—C17B	0.3 (10)
C14A—C15A—C16A—Fe1A	58.4 (7)	C14B—C15B—C16B—Fe1B	59.1 (6)
C15A—C16A—C17A—C13A	−0.5 (10)	C15B—C16B—C17B—C13B	−0.4 (10)
C15A—C16A—C17A—Fe1A	59.0 (6)	C15B—C16B—C17B—Fe1B	59.7 (6)
C17A—C13A—C14A—C15A	−0.5 (11)	C17B—C13B—C14B—C15B	−0.1 (11)
C17A—C13A—C14A—Fe1A	59.1 (6)	C17B—C13B—C14B—Fe1B	59.8 (6)
C18A—C2A—N1A—C3'A	−3.3 (14)	C18B—C2B—N1B—C3'B	5.2 (12)
C18A—C2A—N1A—N2A	177.3 (8)	C18B—C2B—N1B—N2B	−176.1 (7)
C19A—C20A—C21A—C22A	176.7 (10)	C19B—C20B—C21B—C22B	−179.4 (8)
C19A—C20A—C21A—Fe2A	−121.0 (9)	C19B—C20B—C21B—Fe2B	−117.9 (8)
C19A—C20A—C24A—C23A	−176.5 (11)	C19B—C20B—C24B—C23B	179.2 (8)
C19A—C20A—C24A—Fe2A	124.3 (10)	C19B—C20B—C24B—Fe2B	118.5 (8)
C20A—C21A—C22A—C23A	0.0	C20B—C21B—C22B—C23B	2.3 (10)
C20A—C21A—C22A—Fe2A	61.1 (4)	C20B—C21B—C22B—Fe2B	61.6 (6)
C21A—C20A—C24A—C23A	0.0	C21B—C20B—C24B—C23B	3.0 (9)
C21A—C20A—C24A—Fe2A	−59.2 (3)	C21B—C20B—C24B—Fe2B	−57.6 (5)
C21A—C22A—C23A—C24A	0.0	C21B—C22B—C23B—C24B	−0.4 (10)
C21A—C22A—C23A—Fe2A	57.4 (3)	C21B—C22B—C23B—Fe2B	58.6 (6)
C22A—C23A—C24A—C20A	0.0	C22B—C23B—C24B—C20B	−1.7 (10)
C22A—C23A—C24A—Fe2A	56.3 (4)	C22B—C23B—C24B—Fe2B	58.8 (6)
C24A—C20A—C21A—C22A	0.0	C24B—C20B—C21B—C22B	−3.2 (9)
C24A—C20A—C21A—Fe2A	62.3 (4)	C24B—C20B—C21B—Fe2B	58.2 (5)
C25A—C26A—C27A—C28A	0.8 (13)	C25B—C26B—C27B—C28B	−1.4 (10)
C25A—C26A—C27A—Fe2A	59.8 (8)	C25B—C26B—C27B—Fe2B	57.2 (6)
C26A—C25A—C29A—C28A	2.3 (14)	C26B—C25B—C29B—C28B	−2.4 (10)

C26A—C25A—C29A—Fe2A	−58.9 (8)	C26B—C25B—C29B—Fe2B	−60.5 (6)
C26A—C27A—C28A—C29A	0.6 (14)	C26B—C27B—C28B—C29B	−0.1 (10)
C26A—C27A—C28A—Fe2A	58.5 (8)	C26B—C27B—C28B—Fe2B	60.7 (7)
C27A—C28A—C29A—C25A	−1.8 (15)	C27B—C28B—C29B—C25B	1.6 (10)
C27A—C28A—C29A—Fe2A	59.5 (9)	C27B—C28B—C29B—Fe2B	61.1 (6)
C29A—C25A—C26A—C27A	−1.9 (13)	C29B—C25B—C26B—C27B	2.4 (10)
C29A—C25A—C26A—Fe2A	58.1 (8)	C29B—C25B—C26B—Fe2B	59.6 (6)
Fe1A—C8A—C9A—C10A	−60.3 (6)	Fe1B—C8B—C9B—C10B	−58.6 (6)
Fe1A—C8A—C12A—C11A	61.0 (6)	Fe1B—C8B—C12B—C11B	58.4 (6)
Fe1A—C9A—C10A—C11A	−59.7 (6)	Fe1B—C9B—C10B—C11B	−60.0 (6)
Fe1A—C10A—C11A—C12A	−58.3 (6)	Fe1B—C10B—C11B—C12B	−58.7 (6)
Fe1A—C11A—C12A—C8A	−60.5 (6)	Fe1B—C11B—C12B—C8B	−57.9 (6)
Fe1A—C13A—C14A—C15A	−59.7 (7)	Fe1B—C13B—C14B—C15B	−60.0 (6)
Fe1A—C13A—C17A—C16A	59.6 (6)	Fe1B—C13B—C17B—C16B	60.9 (6)
Fe1A—C14A—C15A—C16A	−59.2 (6)	Fe1B—C14B—C15B—C16B	−59.4 (6)
Fe1A—C15A—C16A—C17A	−58.2 (6)	Fe1B—C15B—C16B—C17B	−58.7 (6)
Fe1A—C16A—C17A—C13A	−59.5 (6)	Fe1B—C16B—C17B—C13B	−60.2 (6)
Fe2A—C20A—C21A—C22A	−62.3 (4)	Fe2B—C20B—C21B—C22B	−61.4 (6)
Fe2A—C20A—C24A—C23A	59.2 (3)	Fe2B—C20B—C24B—C23B	60.6 (6)
Fe2A—C21A—C22A—C23A	−61.1 (4)	Fe2B—C21B—C22B—C23B	−59.3 (6)
Fe2A—C22A—C23A—C24A	−57.4 (3)	Fe2B—C22B—C23B—C24B	−59.0 (6)
Fe2A—C23A—C24A—C20A	−56.3 (4)	Fe2B—C23B—C24B—C20B	−60.5 (6)
Fe2A—C25A—C26A—C27A	−60.0 (8)	Fe2B—C25B—C26B—C27B	−57.2 (6)
Fe2A—C25A—C29A—C28A	61.2 (9)	Fe2B—C25B—C29B—C28B	58.1 (6)
Fe2A—C26A—C27A—C28A	−58.9 (8)	Fe2B—C26B—C27B—C28B	−58.6 (6)
Fe2A—C27A—C28A—C29A	−57.9 (9)	Fe2B—C27B—C28B—C29B	−60.8 (6)
Fe2A—C28A—C29A—C25A	−61.4 (8)	Fe2B—C28B—C29B—C25B	−59.6 (6)
N1A—C3'A—C4'A—C5'A	139.4 (8)	N1B—C3'B—C4'B—C5'B	146.2 (8)
N1A—C3'A—C4'A—O1A	−98.7 (8)	N1B—C3'B—C4'B—O1B	−93.2 (8)
N1A—N2A—N3A—C1A	0.4 (10)	N1B—N2B—N3B—C1B	1.5 (9)
N3A—C1A—C2A—C18A	−176.6 (9)	N3B—C1B—C2B—C18B	176.7 (8)
N3A—C1A—C2A—N1A	−0.5 (9)	N3B—C1B—C2B—N1B	0.2 (9)
N3A—C1A—C8A—C9A	11.2 (13)	N3B—C1B—C8B—C9B	176.8 (8)
N3A—C1A—C8A—C12A	−168.6 (8)	N3B—C1B—C8B—C12B	−3.5 (13)
N3A—C1A—C8A—Fe1A	−78.2 (10)	N3B—C1B—C8B—Fe1B	87.6 (9)
N4A—C1'A—C2'A—C3'A	146.4 (7)	N4B—C1'B—C2'B—C3'B	149.9 (7)
N4A—C1'A—O1A—C4'A	−138.2 (8)	N4B—C1'B—O1B—C4'B	−137.0 (7)
N4A—C3A—N5A—C4A	0.3 (17)	N4B—C3B—N5B—C4B	0.1 (15)

N5A—C3A—N4A—C1'A	176.6 (10)	N5B—C3B—N4B—C1'B	−179.0 (8)
N5A—C3A—N4A—C6A	−3.8 (16)	N5B—C3B—N4B—C6B	0.2 (14)
N5A—C4A—C5A—C6A	−5 (2)	N5B—C4B—C5B—C6B	2.3 (17)
N5A—C4A—C5A—C7A	−179.7 (15)	N5B—C4B—C5B—C7B	−178.5 (12)
O1A—C1'A—C2'A—C3'A	25.6 (9)	O1B—C1'B—C2'B—C3'B	31.1 (9)
O1A—C1'A—N4A—C3A	−130.2 (10)	O1B—C1'B—N4B—C3B	−133.3 (8)
O1A—C1'A—N4A—C6A	50.1 (14)	O1B—C1'B—N4B—C6B	47.5 (12)
O1A—C4'A—C5'A—O2A	−74.5 (9)	O1B—C4'B—C5'B—O2B	−68.9 (9)
O3A—C3A—N4A—C1'A	−3.0 (17)	O3B—C3B—N4B—C1'B	2.6 (15)
O3A—C3A—N4A—C6A	176.6 (12)	O3B—C3B—N4B—C6B	−178.1 (10)
O3A—C3A—N5A—C4A	179.9 (11)	O3B—C3B—N5B—C4B	178.5 (11)
O4A—C4A—C5A—C6A	177.7 (14)	O4B—C4B—C5B—C6B	−177.7 (13)
O4A—C4A—C5A—C7A	3 (2)	O4B—C4B—C5B—C7B	2 (2)
O4A—C4A—N5A—C3A	−178.5 (12)	O4B—C4B—N5B—C3B	178.6 (11)

Table S5. Bond lengths for **2a** [Å].

Molecule A		Molecule B	
C1A—C2A	1.378 (18)	C1B—C2B	1.397 (18)
C1A—C16A	1.435 (17)	C1B—C16B	1.441 (18)
C1A—N3A	1.381 (16)	C1B—N3B	1.356 (16)
C2A—C26A	1.394 (19)	C2B—C26B	1.384 (18)
C2A—N1A	1.405 (17)	C2B—N1B	1.358 (17)
C3A—C4A	1.533 (17)	C3B—C4B	1.510 (18)
C3A—N1A	1.471 (15)	C3B—N1B	1.451 (16)
C4A—C5A	1.519 (19)	C4B—C5B	1.510 (16)
C5A—C6A	1.45 (2)	C5B—C6B	1.492 (18)
C5A—O1A	1.209 (17)	C5B—O1B	1.222 (15)
C6A—C7A	1.43 (2)	C6B—C7B	1.388 (18)
C6A—C10A	1.44 (2)	C6B—C10B	1.453 (17)
C6A—Fe1A	2.030 (14)	C6B—Fe1B	2.047 (12)
C7A—C8A	1.42 (2)	C7B—C8B	1.420 (18)
C7A—Fe1A	2.034 (16)	C7B—Fe1B	2.035 (13)
C8A—C9A	1.40 (2)	C8B—C9B	1.421 (19)
C8A—Fe1A	2.061 (16)	C8B—Fe1B	2.053 (14)
C9A—C10A	1.41 (2)	C9B—C10B	1.443 (19)
C9A—Fe1A	2.044 (14)	C9B—Fe1B	2.045 (13)
C10A—Fe1A	2.015 (14)	C10B—Fe1B	2.035 (12)
C11A—C12A	1.42 (2)	C11B—C12B	1.421 (17)
C11A—C15A	1.46 (2)	C11B—C15B	1.414 (18)
C11A—Fe1A	2.060 (14)	C11B—Fe1B	2.046 (11)
C12A—C13A	1.44 (2)	C12B—C13B	1.408 (19)
C12A—Fe1A	2.068 (15)	C12B—Fe1B	2.066 (13)
C13A—C14A	1.39 (2)	C13B—C14B	1.420 (18)
C13A—Fe1A	2.075 (14)	C13B—Fe1B	2.075 (12)
C14A—C15A	1.40 (2)	C14B—C15B	1.442 (17)
C14A—Fe1A	2.063 (15)	C14B—Fe1B	2.045 (12)
C15A—Fe1A	2.079 (16)	C15B—Fe1B	2.043 (12)
C16A—C17A	1.430 (17)	C16B—C17B	1.448 (18)
C16A—C20A	1.469 (19)	C16B—C20B	1.443 (17)
C16A—Fe2A	2.033 (12)	C16B—Fe2B	2.045 (13)
C17A—C18A	1.451 (18)	C17B—C18B	1.451 (18)
C17A—Fe2A	2.055 (13)	C17B—Fe2B	2.056 (12)
C18A—C19A	1.397 (19)	C18B—C19B	1.428 (18)

C18A—Fe2A	2.054 (13)	C18B—Fe2B	2.054 (12)
C19A—C20A	1.452 (17)	C19B—C20B	1.449 (18)
C19A—Fe2A	2.051 (13)	C19B—Fe2B	2.058 (13)
C20A—Fe2A	2.080 (12)	C20B—Fe2B	2.061 (12)
C21A—C22A	1.442 (18)	C21B—C22B	1.405 (18)
C21A—C25A	1.411 (18)	C21B—C25B	1.411 (19)
C21A—Fe2A	2.034 (13)	C21B—Fe2B	2.031 (13)
C22A—C23A	1.419 (18)	C22B—C23B	1.448 (19)
C22A—Fe2A	2.052 (13)	C22B—Fe2B	2.044 (13)
C23A—C24A	1.426 (18)	C23B—C24B	1.420 (19)
C23A—Fe2A	2.070 (13)	C23B—Fe2B	2.051 (13)
C24A—C25A	1.440 (18)	C24B—C25B	1.449 (19)
C24A—Fe2A	2.055 (13)	C24B—Fe2B	2.066 (14)
C25A—Fe2A	2.045 (13)	C25B—Fe2B	2.034 (13)
C26A—C27A	1.211 (19)	C26B—C27B	1.209 (17)
C27A—C28A	1.414 (17)	C27B—C28B	1.410 (17)
C28A—C29A	1.441 (18)	C28B—C29B	1.452 (17)
C28A—C32A	1.450 (17)	C28B—C32B	1.447 (17)
C28A—Fe3A	2.058 (12)	C28B—Fe3B	2.047 (12)
C29A—C30A	1.421 (19)	C29B—C30B	1.425 (17)
C29A—Fe3A	2.040 (12)	C29B—Fe3B	2.050 (12)
C30A—C31A	1.449 (19)	C30B—C31B	1.428 (18)
C30A—Fe3A	2.052 (13)	C30B—Fe3B	2.054 (13)
C31A—C32A	1.398 (18)	C31B—C32B	1.438 (17)
C31A—Fe3A	2.050 (12)	C31B—Fe3B	2.043 (13)
C32A—Fe3A	2.046 (11)	C32B—Fe3B	2.053 (13)
C33A—C34A	1.40 (2)	C33B—C34B	1.436 (18)
C33A—C37A	1.43 (2)	C33B—C37B	1.407 (19)
C33A—Fe3A	2.033 (13)	C33B—Fe3B	2.058 (13)
C34A—C35A	1.41 (2)	C34B—C35B	1.421 (18)
C34A—Fe3A	2.022 (16)	C34B—Fe3B	2.039 (13)
C35A—C36A	1.41 (2)	C35B—C36B	1.410 (19)
C35A—Fe3A	2.043 (13)	C35B—Fe3B	2.041 (13)
C36A—C37A	1.405 (19)	C36B—C37B	1.427 (18)
C36A—Fe3A	2.042 (12)	C36B—Fe3B	2.056 (13)
C37A—Fe3A	2.035 (11)	C37B—Fe3B	2.036 (13)
N1A—N2A	1.296 (14)	N1B—N2B	1.343 (15)
N2A—N3A	1.327 (15)	N2B—N3B	1.331 (15)

Table S6. Values of valence angles for **2a** [°].

Molecule A		Molecule B	
C2A—C1A—C16A	131.7 (12)	C2B—C1B—C16B	131.4 (12)
C2A—C1A—N3A	108.9 (11)	N3B—C1B—C2B	108.2 (11)
N3A—C1A—C16A	119.3 (11)	N3B—C1B—C16B	120.3 (11)
C1A—C2A—C26A	134.4 (13)	C26B—C2B—C1B	132.8 (12)
C1A—C2A—N1A	102.4 (11)	N1B—C2B—C1B	103.7 (11)
C26A—C2A—N1A	123.0 (12)	N1B—C2B—C26B	123.5 (12)
N1A—C3A—C4A	110.7 (10)	N1B—C3B—C4B	112.3 (11)
C5A—C4A—C3A	110.7 (10)	C3B—C4B—C5B	113.2 (11)
C6A—C5A—C4A	116.5 (11)	C6B—C5B—C4B	117.9 (11)
O1A—C5A—C4A	121.2 (13)	O1B—C5B—C4B	121.7 (12)
O1A—C5A—C6A	122.3 (13)	O1B—C5B—C6B	120.2 (11)
C5A—C6A—Fe1A	123.5 (10)	C5B—C6B—Fe1B	119.5 (8)
C7A—C6A—C5A	129.6 (14)	C7B—C6B—C5B	124.4 (11)
C7A—C6A—C10A	106.1 (13)	C7B—C6B—C10B	108.1 (11)
C7A—C6A—Fe1A	69.5 (9)	C7B—C6B—Fe1B	69.7 (7)
C10A—C6A—C5A	124.2 (14)	C10B—C6B—C5B	126.8 (11)
C10A—C6A—Fe1A	68.6 (8)	C10B—C6B—Fe1B	68.7 (7)
C6A—C7A—Fe1A	69.3 (9)	C6B—C7B—C8B	109.8 (12)
C8A—C7A—C6A	109.0 (14)	C6B—C7B—Fe1B	70.6 (7)
C8A—C7A—Fe1A	70.8 (9)	C8B—C7B—Fe1B	70.4 (7)
C7A—C8A—Fe1A	68.7 (9)	C7B—C8B—C9B	107.5 (12)
C9A—C8A—C7A	107.5 (14)	C7B—C8B—Fe1B	69.0 (7)
C9A—C8A—Fe1A	69.3 (9)	C9B—C8B—Fe1B	69.4 (8)
C8A—C9A—C10A	109.1 (14)	C8B—C9B—C10B	108.3 (12)
C8A—C9A—Fe1A	70.6 (9)	C8B—C9B—Fe1B	70.0 (7)
C10A—C9A—Fe1A	68.6 (8)	C10B—C9B—Fe1B	68.9 (7)
C6A—C10A—Fe1A	69.8 (8)	C6B—C10B—Fe1B	69.6 (7)
C9A—C10A—C6A	108.4 (14)	C9B—C10B—C6B	106.3 (11)
C9A—C10A—Fe1A	70.9 (8)	C9B—C10B—Fe1B	69.6 (7)
C12A—C11A—C15A	107.8 (14)	C12B—C11B—Fe1B	70.5 (7)
C12A—C11A—Fe1A	70.2 (8)	C15B—C11B—C12B	108.3 (11)
C15A—C11A—Fe1A	70.1 (8)	C15B—C11B—Fe1B	69.7 (7)
C11A—C12A—C13A	107.6 (14)	C11B—C12B—Fe1B	69.0 (7)
C11A—C12A—Fe1A	69.6 (8)	C13B—C12B—C11B	108.6 (11)
C13A—C12A—Fe1A	69.8 (8)	C13B—C12B—Fe1B	70.5 (7)

C12A—C13A—Fe1A	69.4 (8)	C12B—C13B—C14B	107.9 (11)
C14A—C13A—C12A	107.3 (14)	C12B—C13B—Fe1B	69.8 (7)
C14A—C13A—Fe1A	69.9 (9)	C14B—C13B—Fe1B	68.7 (7)
C13A—C14A—C15A	111.0 (15)	C13B—C14B—C15B	108.0 (11)
C13A—C14A—Fe1A	70.8 (9)	C13B—C14B—Fe1B	71.0 (7)
C15A—C14A—Fe1A	70.8 (9)	C15B—C14B—Fe1B	69.3 (7)
C11A—C15A—Fe1A	68.7 (9)	C11B—C15B—C14B	107.2 (11)
C14A—C15A—C11A	106.2 (14)	C11B—C15B—Fe1B	69.9 (7)
C14A—C15A—Fe1A	69.6 (9)	C14B—C15B—Fe1B	69.4 (7)
C1A—C16A—C20A	125.1 (11)	C1B—C16B—C17B	123.5 (11)
C1A—C16A—Fe2A	125.7 (9)	C1B—C16B—C20B	128.2 (12)
C17A—C16A—C1A	127.3 (12)	C1B—C16B—Fe2B	124.2 (9)
C17A—C16A—C20A	107.6 (11)	C17B—C16B—Fe2B	69.7 (7)
C17A—C16A—Fe2A	70.4 (7)	C20B—C16B—C17B	108.3 (11)
C20A—C16A—Fe2A	70.8 (7)	C20B—C16B—Fe2B	70.0 (7)
C16A—C17A—C18A	108.1 (12)	C16B—C17B—C18B	107.7 (11)
C16A—C17A—Fe2A	68.7 (7)	C16B—C17B—Fe2B	68.9 (7)
C18A—C17A—Fe2A	69.3 (7)	C18B—C17B—Fe2B	69.3 (7)
C17A—C18A—Fe2A	69.4 (7)	C17B—C18B—Fe2B	69.4 (7)
C19A—C18A—C17A	108.5 (11)	C19B—C18B—C17B	107.8 (11)
C19A—C18A—Fe2A	70.0 (8)	C19B—C18B—Fe2B	69.8 (7)
C18A—C19A—C20A	109.4 (12)	C18B—C19B—C20B	109.0 (11)
C18A—C19A—Fe2A	70.2 (8)	C18B—C19B—Fe2B	69.5 (7)
C20A—C19A—Fe2A	70.5 (7)	C20B—C19B—Fe2B	69.5 (7)
C16A—C20A—Fe2A	67.4 (7)	C16B—C20B—C19B	107.2 (11)
C19A—C20A—C16A	106.3 (11)	C16B—C20B—Fe2B	68.8 (7)
C19A—C20A—Fe2A	68.3 (7)	C19B—C20B—Fe2B	69.3 (7)
C22A—C21A—Fe2A	70.0 (7)	C22B—C21B—C25B	109.2 (12)
C25A—C21A—C22A	107.9 (12)	C22B—C21B—Fe2B	70.4 (8)
C25A—C21A—Fe2A	70.2 (8)	C25B—C21B—Fe2B	69.8 (8)
C21A—C22A—Fe2A	68.7 (7)	C21B—C22B—C23B	108.0 (12)
C23A—C22A—C21A	107.8 (12)	C21B—C22B—Fe2B	69.3 (7)
C23A—C22A—Fe2A	70.6 (8)	C23B—C22B—Fe2B	69.6 (8)
C22A—C23A—C24A	108.6 (12)	C22B—C23B—Fe2B	69.0 (7)
C22A—C23A—Fe2A	69.2 (7)	C24B—C23B—C22B	107.5 (12)
C24A—C23A—Fe2A	69.2 (7)	C24B—C23B—Fe2B	70.4 (8)
C23A—C24A—C25A	107.3 (12)	C23B—C24B—C25B	107.6 (12)
C23A—C24A—Fe2A	70.3 (7)	C23B—C24B—Fe2B	69.3 (8)

C25A—C24A—Fe2A	69.1 (7)	C25B—C24B—Fe2B	68.2 (8)
C21A—C25A—C24A	108.5 (12)	C21B—C25B—C24B	107.7 (12)
C21A—C25A—Fe2A	69.3 (7)	C21B—C25B—Fe2B	69.5 (7)
C24A—C25A—Fe2A	69.8 (7)	C24B—C25B—Fe2B	70.5 (8)
C27A—C26A—C2A	174.6 (15)	C27B—C26B—C2B	178.0 (14)
C26A—C27A—C28A	177.2 (14)	C26B—C27B—C28B	178.8 (15)
C27A—C28A—C29A	126.6 (12)	C27B—C28B—C29B	124.9 (11)
C27A—C28A—C32A	126.4 (12)	C27B—C28B—C32B	127.6 (11)
C27A—C28A—Fe3A	126.7 (9)	C27B—C28B—Fe3B	122.6 (9)
C29A—C28A—C32A	107.0 (10)	C29B—C28B—Fe3B	69.3 (7)
C29A—C28A—Fe3A	68.7 (7)	C32B—C28B—C29B	107.3 (10)
C32A—C28A—Fe3A	68.8 (6)	C32B—C28B—Fe3B	69.6 (7)
C28A—C29A—Fe3A	70.1 (7)	C28B—C29B—Fe3B	69.1 (7)
C30A—C29A—C28A	108.2 (11)	C30B—C29B—C28B	108.1 (11)
C30A—C29A—Fe3A	70.2 (7)	C30B—C29B—Fe3B	69.8 (7)
C29A—C30A—C31A	107.8 (11)	C29B—C30B—C31B	108.4 (11)
C29A—C30A—Fe3A	69.2 (7)	C29B—C30B—Fe3B	69.5 (7)
C31A—C30A—Fe3A	69.2 (7)	C31B—C30B—Fe3B	69.2 (7)
C30A—C31A—Fe3A	69.4 (7)	C30B—C31B—C32B	108.5 (11)
C32A—C31A—C30A	108.3 (11)	C30B—C31B—Fe3B	70.0 (7)
C32A—C31A—Fe3A	69.9 (7)	C32B—C31B—Fe3B	69.8 (7)
C28A—C32A—Fe3A	69.8 (6)	C28B—C32B—Fe3B	69.1 (7)
C31A—C32A—C28A	108.7 (11)	C31B—C32B—C28B	107.6 (11)
C31A—C32A—Fe3A	70.2 (7)	C31B—C32B—Fe3B	69.1 (7)
C34A—C33A—C37A	106.4 (12)	C34B—C33B—Fe3B	68.8 (7)
C34A—C33A—Fe3A	69.3 (9)	C37B—C33B—C34B	107.5 (11)
C37A—C33A—Fe3A	69.4 (7)	C37B—C33B—Fe3B	69.1 (8)
C33A—C34A—C35A	109.7 (13)	C33B—C34B—Fe3B	70.2 (7)
C33A—C34A—Fe3A	70.2 (8)	C35B—C34B—C33B	107.4 (12)
C35A—C34A—Fe3A	70.5 (9)	C35B—C34B—Fe3B	69.7 (8)
C34A—C35A—C36A	107.2 (13)	C34B—C35B—Fe3B	69.6 (7)
C34A—C35A—Fe3A	68.9 (8)	C36B—C35B—C34B	108.9 (12)
C36A—C35A—Fe3A	69.8 (7)	C36B—C35B—Fe3B	70.4 (8)
C35A—C36A—Fe3A	69.8 (7)	C35B—C36B—C37B	107.3 (12)
C37A—C36A—C35A	108.3 (12)	C35B—C36B—Fe3B	69.3 (7)
C37A—C36A—Fe3A	69.5 (7)	C37B—C36B—Fe3B	68.8 (7)
C33A—C37A—Fe3A	69.3 (7)	C33B—C37B—C36B	108.9 (12)
C36A—C37A—C33A	108.4 (12)	C33B—C37B—Fe3B	70.7 (8)

C36A—C37A—Fe3A	70.1 (7)	C36B—C37B—Fe3B	70.3 (7)
C6A—Fe1A—C7A	41.2 (6)	C6B—Fe1B—C8B	68.2 (5)
C6A—Fe1A—C8A	69.0 (6)	C6B—Fe1B—C12B	128.5 (5)
C6A—Fe1A—C9A	68.9 (6)	C6B—Fe1B—C13B	165.5 (5)
C6A—Fe1A—C11A	112.4 (6)	C7B—Fe1B—C6B	39.8 (5)
C6A—Fe1A—C12A	116.0 (6)	C7B—Fe1B—C8B	40.6 (5)
C6A—Fe1A—C13A	145.3 (6)	C7B—Fe1B—C9B	68.3 (5)
C6A—Fe1A—C14A	175.3 (6)	C7B—Fe1B—C11B	127.6 (5)
C6A—Fe1A—C15A	137.3 (6)	C7B—Fe1B—C12B	165.4 (5)
C7A—Fe1A—C8A	40.5 (6)	C7B—Fe1B—C13B	153.5 (5)
C7A—Fe1A—C9A	67.8 (6)	C7B—Fe1B—C14B	119.4 (5)
C7A—Fe1A—C11A	117.7 (6)	C7B—Fe1B—C15B	108.0 (5)
C7A—Fe1A—C12A	148.1 (6)	C8B—Fe1B—C12B	152.9 (5)
C7A—Fe1A—C13A	170.9 (6)	C8B—Fe1B—C13B	119.1 (5)
C7A—Fe1A—C14A	134.4 (6)	C9B—Fe1B—C6B	69.0 (5)
C7A—Fe1A—C15A	111.7 (6)	C9B—Fe1B—C8B	40.6 (5)
C8A—Fe1A—C12A	171.0 (6)	C9B—Fe1B—C11B	153.5 (5)
C8A—Fe1A—C13A	130.9 (6)	C9B—Fe1B—C12B	119.3 (5)
C8A—Fe1A—C14A	108.4 (6)	C9B—Fe1B—C13B	107.7 (5)
C8A—Fe1A—C15A	113.8 (6)	C10B—Fe1B—C6B	41.7 (5)
C9A—Fe1A—C8A	40.0 (6)	C10B—Fe1B—C7B	68.8 (5)
C9A—Fe1A—C11A	173.3 (6)	C10B—Fe1B—C8B	69.2 (5)
C9A—Fe1A—C12A	133.1 (6)	C10B—Fe1B—C9B	41.4 (5)
C9A—Fe1A—C13A	107.1 (6)	C10B—Fe1B—C11B	118.8 (5)
C9A—Fe1A—C14A	111.9 (6)	C10B—Fe1B—C12B	107.9 (5)
C9A—Fe1A—C15A	142.1 (6)	C10B—Fe1B—C13B	126.6 (5)
C10A—Fe1A—C6A	41.6 (6)	C10B—Fe1B—C14B	164.1 (5)
C10A—Fe1A—C7A	68.9 (6)	C10B—Fe1B—C15B	152.8 (5)
C10A—Fe1A—C8A	68.3 (6)	C11B—Fe1B—C6B	108.5 (5)
C10A—Fe1A—C9A	40.5 (6)	C11B—Fe1B—C8B	164.9 (5)
C10A—Fe1A—C11A	136.2 (6)	C11B—Fe1B—C12B	40.4 (5)
C10A—Fe1A—C12A	109.9 (7)	C11B—Fe1B—C13B	67.8 (5)
C10A—Fe1A—C13A	112.5 (6)	C12B—Fe1B—C13B	39.8 (5)
C10A—Fe1A—C14A	141.8 (6)	C14B—Fe1B—C6B	152.9 (5)
C10A—Fe1A—C15A	177.4 (6)	C14B—Fe1B—C8B	107.5 (5)
C11A—Fe1A—C8A	146.6 (6)	C14B—Fe1B—C9B	126.2 (5)
C11A—Fe1A—C12A	40.2 (6)	C14B—Fe1B—C11B	68.4 (5)
C11A—Fe1A—C13A	67.9 (6)	C14B—Fe1B—C12B	67.6 (5)

C11A—Fe1A—C14A	67.4 (6)	C14B—Fe1B—C13B	40.3 (5)
C11A—Fe1A—C15A	41.2 (6)	C15B—Fe1B—C6B	118.5 (5)
C12A—Fe1A—C13A	40.8 (6)	C15B—Fe1B—C8B	127.0 (5)
C12A—Fe1A—C15A	68.2 (6)	C15B—Fe1B—C9B	164.5 (5)
C13A—Fe1A—C15A	67.4 (6)	C15B—Fe1B—C11B	40.5 (5)
C14A—Fe1A—C12A	67.1 (6)	C15B—Fe1B—C12B	68.0 (5)
C14A—Fe1A—C13A	39.3 (6)	C15B—Fe1B—C13B	68.4 (5)
C14A—Fe1A—C15A	39.6 (6)	C15B—Fe1B—C14B	41.3 (5)
C16A—Fe2A—C17A	40.9 (5)	C16B—Fe2B—C17B	41.4 (5)
C16A—Fe2A—C18A	69.6 (5)	C16B—Fe2B—C18B	69.7 (5)
C16A—Fe2A—C19A	69.9 (5)	C16B—Fe2B—C19B	69.1 (5)
C16A—Fe2A—C20A	41.8 (5)	C16B—Fe2B—C20B	41.2 (5)
C16A—Fe2A—C21A	105.7 (5)	C16B—Fe2B—C23B	162.8 (5)
C16A—Fe2A—C22A	121.1 (5)	C16B—Fe2B—C24B	154.0 (5)
C16A—Fe2A—C23A	157.4 (5)	C17B—Fe2B—C19B	68.9 (5)
C16A—Fe2A—C24A	159.9 (5)	C17B—Fe2B—C20B	69.4 (5)
C16A—Fe2A—C25A	122.5 (5)	C17B—Fe2B—C24B	119.4 (5)
C17A—Fe2A—C20A	68.9 (5)	C18B—Fe2B—C17B	41.3 (5)
C17A—Fe2A—C23A	160.7 (5)	C18B—Fe2B—C19B	40.6 (5)
C18A—Fe2A—C17A	41.3 (5)	C18B—Fe2B—C20B	69.4 (5)
C18A—Fe2A—C20A	68.4 (5)	C18B—Fe2B—C24B	107.5 (6)
C18A—Fe2A—C23A	123.8 (5)	C19B—Fe2B—C20B	41.2 (5)
C18A—Fe2A—C24A	107.8 (5)	C19B—Fe2B—C24B	126.4 (6)
C19A—Fe2A—C17A	68.5 (5)	C20B—Fe2B—C24B	163.9 (5)
C19A—Fe2A—C18A	39.8 (5)	C21B—Fe2B—C16B	105.0 (5)
C19A—Fe2A—C20A	41.1 (5)	C21B—Fe2B—C17B	122.4 (5)
C19A—Fe2A—C22A	123.4 (5)	C21B—Fe2B—C18B	160.3 (5)
C19A—Fe2A—C23A	107.8 (5)	C21B—Fe2B—C19B	156.7 (5)
C19A—Fe2A—C24A	121.7 (5)	C21B—Fe2B—C20B	119.7 (5)
C21A—Fe2A—C17A	121.4 (5)	C21B—Fe2B—C22B	40.3 (5)
C21A—Fe2A—C18A	158.3 (5)	C21B—Fe2B—C23B	68.9 (5)
C21A—Fe2A—C19A	160.3 (5)	C21B—Fe2B—C24B	68.6 (5)
C21A—Fe2A—C20A	123.0 (5)	C21B—Fe2B—C25B	40.6 (5)
C21A—Fe2A—C22A	41.3 (5)	C22B—Fe2B—C16B	123.7 (5)
C21A—Fe2A—C23A	68.5 (5)	C22B—Fe2B—C17B	159.9 (5)
C21A—Fe2A—C24A	68.9 (5)	C22B—Fe2B—C18B	158.0 (5)
C21A—Fe2A—C25A	40.5 (5)	C22B—Fe2B—C19B	123.2 (5)
C22A—Fe2A—C17A	157.6 (5)	C22B—Fe2B—C20B	108.1 (5)

C22A—Fe2A—C18A	159.3 (5)	C22B—Fe2B—C23B	41.4 (5)
C22A—Fe2A—C20A	106.7 (5)	C22B—Fe2B—C24B	68.5 (5)
C22A—Fe2A—C23A	40.3 (5)	C23B—Fe2B—C17B	155.5 (5)
C22A—Fe2A—C24A	68.5 (5)	C23B—Fe2B—C18B	121.5 (5)
C23A—Fe2A—C20A	121.6 (5)	C23B—Fe2B—C19B	109.8 (5)
C24A—Fe2A—C17A	124.1 (5)	C23B—Fe2B—C20B	126.8 (5)
C24A—Fe2A—C20A	157.3 (5)	C23B—Fe2B—C24B	40.4 (5)
C24A—Fe2A—C23A	40.4 (5)	C25B—Fe2B—C16B	117.4 (5)
C25A—Fe2A—C17A	107.6 (5)	C25B—Fe2B—C17B	104.5 (5)
C25A—Fe2A—C18A	123.0 (5)	C25B—Fe2B—C18B	123.8 (5)
C25A—Fe2A—C19A	157.9 (5)	C25B—Fe2B—C19B	162.3 (5)
C25A—Fe2A—C20A	159.8 (5)	C25B—Fe2B—C20B	153.6 (5)
C25A—Fe2A—C22A	68.5 (5)	C25B—Fe2B—C22B	68.5 (5)
C25A—Fe2A—C23A	68.2 (5)	C25B—Fe2B—C23B	69.1 (5)
C25A—Fe2A—C24A	41.1 (5)	C25B—Fe2B—C24B	41.4 (5)
C29A—Fe3A—C28A	41.2 (5)	C28B—Fe3B—C29B	41.5 (5)
C29A—Fe3A—C30A	40.6 (5)	C28B—Fe3B—C30B	69.2 (5)
C29A—Fe3A—C31A	69.1 (5)	C28B—Fe3B—C32B	41.3 (5)
C29A—Fe3A—C32A	69.3 (5)	C28B—Fe3B—C33B	106.7 (5)
C29A—Fe3A—C35A	156.6 (6)	C28B—Fe3B—C36B	152.8 (5)
C29A—Fe3A—C36A	122.4 (5)	C29B—Fe3B—C30B	40.6 (5)
C30A—Fe3A—C28A	68.6 (5)	C29B—Fe3B—C32B	69.4 (5)
C31A—Fe3A—C28A	68.6 (5)	C29B—Fe3B—C33B	125.3 (5)
C31A—Fe3A—C30A	41.4 (5)	C29B—Fe3B—C36B	118.1 (5)
C32A—Fe3A—C28A	41.4 (5)	C30B—Fe3B—C33B	162.5 (5)
C32A—Fe3A—C30A	68.5 (5)	C30B—Fe3B—C36B	107.2 (5)
C32A—Fe3A—C31A	39.9 (5)	C31B—Fe3B—C28B	69.4 (5)
C33A—Fe3A—C28A	109.3 (5)	C31B—Fe3B—C29B	68.9 (5)
C33A—Fe3A—C29A	125.7 (6)	C31B—Fe3B—C30B	40.8 (5)
C33A—Fe3A—C30A	161.5 (6)	C31B—Fe3B—C32B	41.1 (5)
C33A—Fe3A—C31A	156.5 (6)	C31B—Fe3B—C33B	155.3 (5)
C33A—Fe3A—C32A	122.9 (5)	C31B—Fe3B—C36B	126.5 (5)
C33A—Fe3A—C35A	68.7 (6)	C32B—Fe3B—C30B	69.0 (5)
C33A—Fe3A—C36A	68.7 (6)	C32B—Fe3B—C33B	119.9 (5)
C33A—Fe3A—C37A	41.2 (6)	C32B—Fe3B—C36B	164.6 (5)
C34A—Fe3A—C28A	124.5 (6)	C34B—Fe3B—C28B	126.3 (5)
C34A—Fe3A—C29A	162.0 (6)	C34B—Fe3B—C29B	163.9 (5)
C34A—Fe3A—C30A	155.9 (6)	C34B—Fe3B—C30B	154.7 (5)

C34A—Fe3A—C31A	120.0 (6)	C34B—Fe3B—C31B	120.5 (5)
C34A—Fe3A—C32A	106.9 (5)	C34B—Fe3B—C32B	108.3 (5)
C34A—Fe3A—C33A	40.5 (6)	C34B—Fe3B—C33B	41.0 (5)
C34A—Fe3A—C35A	40.6 (6)	C34B—Fe3B—C35B	40.8 (5)
C34A—Fe3A—C36A	68.0 (6)	C34B—Fe3B—C36B	68.4 (5)
C34A—Fe3A—C37A	68.1 (6)	C35B—Fe3B—C28B	164.9 (5)
C35A—Fe3A—C28A	159.2 (6)	C35B—Fe3B—C29B	153.0 (5)
C35A—Fe3A—C30A	120.0 (6)	C35B—Fe3B—C30B	119.8 (5)
C35A—Fe3A—C31A	104.6 (5)	C35B—Fe3B—C31B	108.8 (5)
C35A—Fe3A—C32A	121.0 (6)	C35B—Fe3B—C32B	127.6 (5)
C36A—Fe3A—C28A	159.7 (5)	C35B—Fe3B—C33B	68.4 (5)
C36A—Fe3A—C30A	106.5 (6)	C35B—Fe3B—C36B	40.3 (5)
C36A—Fe3A—C31A	121.6 (5)	C36B—Fe3B—C33B	68.2 (5)
C36A—Fe3A—C32A	157.0 (5)	C37B—Fe3B—C28B	117.9 (5)
C36A—Fe3A—C35A	40.5 (6)	C37B—Fe3B—C29B	106.1 (5)
C37A—Fe3A—C28A	124.9 (5)	C37B—Fe3B—C30B	125.4 (5)
C37A—Fe3A—C29A	109.3 (5)	C37B—Fe3B—C31B	163.5 (5)
C37A—Fe3A—C30A	123.8 (5)	C37B—Fe3B—C32B	153.5 (5)
C37A—Fe3A—C31A	159.1 (6)	C37B—Fe3B—C33B	40.2 (5)
C37A—Fe3A—C32A	160.6 (5)	C37B—Fe3B—C34B	68.5 (5)
C37A—Fe3A—C35A	68.2 (5)	C37B—Fe3B—C35B	68.2 (5)
C37A—Fe3A—C36A	40.3 (6)	C37B—Fe3B—C36B	40.8 (5)
C2A—N1A—C3A	125.0 (10)	C2B—N1B—C3B	129.6 (11)
N2A—N1A—C2A	112.1 (10)	N2B—N1B—C2B	111.9 (10)
N2A—N1A—C3A	122.9 (10)	N2B—N1B—C3B	118.5 (11)
N1A—N2A—N3A	108.5 (10)	N3B—N2B—N1B	106.5 (10)
N2A—N3A—C1A	108.1 (10)	N2B—N3B—C1B	109.6 (10)

Table S7. Values of torsion angles for **2a** [°].

Molecule A		Molecule B	
C1A—C2A—N1A—C3A	−179.1 (11)	C1B—C2B—N1B—C3B	177.5 (12)
C1A—C2A—N1A—N2A	−0.6 (14)	C1B—C2B—N1B—N2B	−1.6 (13)
C1A—C16A—C17A—C18A	−178.7 (12)	C1B—C16B—C17B—C18B	−176.8 (11)
C1A—C16A—C17A—Fe2A	−120.5 (13)	C1B—C16B—C17B—Fe2B	−118.3 (12)
C1A—C16A—C20A—C19A	178.1 (11)	C1B—C16B—C20B—C19B	177.2 (12)
C1A—C16A—C20A—Fe2A	120.7 (12)	C1B—C16B—C20B—Fe2B	118.3 (13)
C2A—C1A—C16A—C17A	−172.2 (13)	C2B—C1B—C16B—C17B	170.5 (12)
C2A—C1A—C16A—C20A	6 (2)	C2B—C1B—C16B—C20B	−7 (2)
C2A—C1A—C16A—Fe2A	96.2 (16)	C2B—C1B—C16B—Fe2B	83.6 (17)
C2A—C1A—N3A—N2A	−0.2 (14)	C2B—C1B—N3B—N2B	2.5 (14)
C2A—N1A—N2A—N3A	0.5 (14)	C2B—N1B—N2B—N3B	3.1 (14)
C3A—C4A—C5A—C6A	−178.2 (12)	C3B—C4B—C5B—C6B	−173.6 (10)
C3A—C4A—C5A—O1A	−1 (2)	C3B—C4B—C5B—O1B	11.3 (17)
C3A—N1A—N2A—N3A	179.0 (10)	C3B—N1B—N2B—N3B	−176.1 (11)
C4A—C3A—N1A—C2A	−165.9 (11)	C4B—C3B—N1B—C2B	−126.8 (13)
C4A—C3A—N1A—N2A	15.8 (16)	C4B—C3B—N1B—N2B	52.3 (15)
C4A—C5A—C6A—C7A	1 (2)	C4B—C5B—C6B—C7B	−177.1 (11)
C4A—C5A—C6A—C10A	−174.4 (13)	C4B—C5B—C6B—C10B	14.1 (17)
C4A—C5A—C6A—Fe1A	−88.9 (15)	C4B—C5B—C6B—Fe1B	98.4 (12)
C5A—C6A—C7A—C8A	−177.0 (14)	C5B—C6B—C7B—C8B	−172.1 (11)
C5A—C6A—C7A—Fe1A	−117.1 (16)	C5B—C6B—C7B—Fe1B	−112.5 (11)
C5A—C6A—C10A—C9A	177.4 (13)	C5B—C6B—C10B—C9B	171.9 (11)
C5A—C6A—C10A—Fe1A	116.8 (14)	C5B—C6B—C10B—Fe1B	111.6 (11)
C6A—C7A—C8A—C9A	0.2 (17)	C6B—C7B—C8B—C9B	0.8 (14)
C6A—C7A—C8A—Fe1A	58.9 (10)	C6B—C7B—C8B—Fe1B	59.7 (9)
C7A—C6A—C10A—C9A	0.9 (16)	C7B—C6B—C10B—C9B	1.6 (13)
C7A—C6A—C10A—Fe1A	−59.7 (10)	C7B—C6B—C10B—Fe1B	−58.7 (8)
C7A—C8A—C9A—C10A	0.4 (17)	C7B—C8B—C9B—C10B	0.3 (14)
C7A—C8A—C9A—Fe1A	58.3 (10)	C7B—C8B—C9B—Fe1B	58.7 (9)
C8A—C9A—C10A—C6A	−0.8 (16)	C8B—C9B—C10B—C6B	−1.1 (13)
C8A—C9A—C10A—Fe1A	59.2 (10)	C8B—C9B—C10B—Fe1B	59.1 (9)
C10A—C6A—C7A—C8A	−0.7 (16)	C10B—C6B—C7B—C8B	−1.5 (14)
C10A—C6A—C7A—Fe1A	59.2 (9)	C10B—C6B—C7B—Fe1B	58.1 (8)
C11A—C12A—C13A—C14A	−0.3 (16)	C11B—C12B—C13B—C14B	0.4 (14)
C11A—C12A—C13A—Fe1A	59.5 (10)	C11B—C12B—C13B—Fe1B	58.6 (8)

C12A—C11A—C15A—C14A	−0.5 (16)	C12B—C11B—C15B—C14B	−0.5 (13)
C12A—C11A—C15A—Fe1A	−60.3 (10)	C12B—C11B—C15B—Fe1B	−60.2 (8)
C12A—C13A—C14A—C15A	0.0 (17)	C12B—C13B—C14B—C15B	−0.7 (14)
C12A—C13A—C14A—Fe1A	59.6 (10)	C12B—C13B—C14B—Fe1B	58.9 (9)
C13A—C14A—C15A—C11A	0.3 (16)	C13B—C14B—C15B—C11B	0.8 (13)
C13A—C14A—C15A—Fe1A	59.5 (11)	C13B—C14B—C15B—Fe1B	60.7 (8)
C15A—C11A—C12A—C13A	0.5 (16)	C15B—C11B—C12B—C13B	0.1 (14)
C15A—C11A—C12A—Fe1A	60.2 (10)	C15B—C11B—C12B—Fe1B	59.6 (8)
C16A—C1A—C2A—C26A	9 (3)	C16B—C1B—C2B—C26B	4 (2)
C16A—C1A—C2A—N1A	−175.5 (12)	C16B—C1B—C2B—N1B	−176.1 (13)
C16A—C1A—N3A—N2A	176.3 (11)	C16B—C1B—N3B—N2B	178.6 (11)
C16A—C17A—C18A—C19A	−1.3 (14)	C16B—C17B—C18B—C19B	−1.1 (14)
C16A—C17A—C18A—Fe2A	57.9 (8)	C16B—C17B—C18B—Fe2B	58.4 (8)
C17A—C16A—C20A—C19A	−3.7 (13)	C17B—C16B—C20B—C19B	−0.6 (13)
C17A—C16A—C20A—Fe2A	−61.1 (8)	C17B—C16B—C20B—Fe2B	−59.4 (9)
C17A—C18A—C19A—C20A	−1.0 (15)	C17B—C18B—C19B—C20B	0.8 (14)
C17A—C18A—C19A—Fe2A	58.8 (9)	C17B—C18B—C19B—Fe2B	59.2 (8)
C18A—C19A—C20A—C16A	2.9 (14)	C18B—C19B—C20B—C16B	−0.1 (13)
C18A—C19A—C20A—Fe2A	59.7 (9)	C18B—C19B—C20B—Fe2B	58.4 (9)
C20A—C16A—C17A—C18A	3.1 (14)	C20B—C16B—C17B—C18B	1.1 (14)
C20A—C16A—C17A—Fe2A	61.3 (8)	C20B—C16B—C17B—Fe2B	59.6 (8)
C21A—C22A—C23A—C24A	0.7 (14)	C21B—C22B—C23B—C24B	−1.3 (15)
C21A—C22A—C23A—Fe2A	58.8 (8)	C21B—C22B—C23B—Fe2B	58.8 (9)
C22A—C21A—C25A—C24A	−1.1 (14)	C22B—C21B—C25B—C24B	0.9 (15)
C22A—C21A—C25A—Fe2A	−60.1 (9)	C22B—C21B—C25B—Fe2B	−59.6 (9)
C22A—C23A—C24A—C25A	−1.4 (14)	C22B—C23B—C24B—C25B	1.8 (15)
C22A—C23A—C24A—Fe2A	58.1 (9)	C22B—C23B—C24B—Fe2B	59.3 (9)
C23A—C24A—C25A—C21A	1.5 (14)	C23B—C24B—C25B—C21B	−1.7 (15)
C23A—C24A—C25A—Fe2A	60.3 (9)	C23B—C24B—C25B—Fe2B	58.2 (10)
C25A—C21A—C22A—C23A	0.2 (14)	C25B—C21B—C22B—C23B	0.3 (14)
C25A—C21A—C22A—Fe2A	60.2 (9)	C25B—C21B—C22B—Fe2B	59.2 (9)
C26A—C2A—N1A—C3A	−3.2 (19)	C26B—C2B—N1B—C3B	−3 (2)
C26A—C2A—N1A—N2A	175.3 (12)	C26B—C2B—N1B—N2B	177.9 (11)
C27A—C28A—C29A—C30A	179.2 (12)	C27B—C28B—C29B—C30B	−175.1 (12)
C27A—C28A—C29A—Fe3A	−120.7 (13)	C27B—C28B—C29B—Fe3B	−116.0 (12)
C27A—C28A—C32A—C31A	−179.6 (12)	C27B—C28B—C32B—C31B	174.5 (12)
C27A—C28A—C32A—Fe3A	120.8 (12)	C27B—C28B—C32B—Fe3B	116.0 (13)
C28A—C29A—C30A—C31A	1.4 (14)	C28B—C29B—C30B—C31B	0.3 (14)

C28A—C29A—C30A—Fe3A	60.0 (8)	C28B—C29B—C30B—Fe3B	58.7 (8)
C29A—C28A—C32A—C31A	1.3 (13)	C29B—C28B—C32B—C31B	−0.8 (14)
C29A—C28A—C32A—Fe3A	−58.4 (8)	C29B—C28B—C32B—Fe3B	−59.3 (8)
C29A—C30A—C31A—C32A	−0.6 (14)	C29B—C30B—C31B—C32B	−0.8 (15)
C29A—C30A—C31A—Fe3A	58.6 (9)	C29B—C30B—C31B—Fe3B	58.6 (9)
C30A—C31A—C32A—C28A	−0.4 (14)	C30B—C31B—C32B—C28B	1.0 (14)
C30A—C31A—C32A—Fe3A	59.0 (9)	C30B—C31B—C32B—Fe3B	59.5 (9)
C32A—C28A—C29A—C30A	−1.7 (13)	C32B—C28B—C29B—C30B	0.3 (14)
C32A—C28A—C29A—Fe3A	58.4 (8)	C32B—C28B—C29B—Fe3B	59.4 (8)
C33A—C34A—C35A—C36A	−0.1 (16)	C33B—C34B—C35B—C36B	0.6 (14)
C33A—C34A—C35A—Fe3A	59.4 (10)	C33B—C34B—C35B—Fe3B	60.3 (9)
C34A—C33A—C37A—C36A	−0.2 (14)	C34B—C33B—C37B—C36B	2.0 (15)
C34A—C33A—C37A—Fe3A	−59.7 (9)	C34B—C33B—C37B—Fe3B	−58.2 (9)
C34A—C35A—C36A—C37A	−0.1 (15)	C34B—C35B—C36B—C37B	0.6 (15)
C34A—C35A—C36A—Fe3A	59.0 (10)	C34B—C35B—C36B—Fe3B	59.1 (9)
C35A—C36A—C37A—C33A	0.2 (14)	C35B—C36B—C37B—C33B	−1.6 (15)
C35A—C36A—C37A—Fe3A	59.2 (9)	C35B—C36B—C37B—Fe3B	58.8 (9)
C37A—C33A—C34A—C35A	0.2 (16)	C37B—C33B—C34B—C35B	−1.6 (14)
C37A—C33A—C34A—Fe3A	59.8 (9)	C37B—C33B—C34B—Fe3B	58.4 (9)
Fe1A—C6A—C7A—C8A	−59.9 (11)	Fe1B—C6B—C7B—C8B	−59.6 (9)
Fe1A—C6A—C10A—C9A	60.6 (10)	Fe1B—C6B—C10B—C9B	60.3 (8)
Fe1A—C7A—C8A—C9A	−58.7 (10)	Fe1B—C7B—C8B—C9B	−59.0 (9)
Fe1A—C8A—C9A—C10A	−57.9 (10)	Fe1B—C8B—C9B—C10B	−58.4 (9)
Fe1A—C9A—C10A—C6A	−60.0 (10)	Fe1B—C9B—C10B—C6B	−60.3 (8)
Fe1A—C11A—C12A—C13A	−59.7 (10)	Fe1B—C11B—C12B—C13B	−59.5 (9)
Fe1A—C11A—C15A—C14A	59.8 (10)	Fe1B—C11B—C15B—C14B	59.7 (8)
Fe1A—C12A—C13A—C14A	−59.9 (10)	Fe1B—C12B—C13B—C14B	−58.2 (9)
Fe1A—C13A—C14A—C15A	−59.6 (11)	Fe1B—C13B—C14B—C15B	−59.6 (8)
Fe1A—C14A—C15A—C11A	−59.2 (10)	Fe1B—C14B—C15B—C11B	−60.0 (8)
Fe2A—C16A—C17A—C18A	−58.2 (8)	Fe2B—C16B—C17B—C18B	−58.6 (8)
Fe2A—C16A—C20A—C19A	57.4 (8)	Fe2B—C16B—C20B—C19B	58.9 (8)
Fe2A—C17A—C18A—C19A	−59.2 (9)	Fe2B—C17B—C18B—C19B	−59.5 (8)
Fe2A—C18A—C19A—C20A	−59.9 (9)	Fe2B—C18B—C19B—C20B	−58.4 (8)
Fe2A—C19A—C20A—C16A	−56.8 (8)	Fe2B—C19B—C20B—C16B	−58.6 (8)
Fe2A—C21A—C22A—C23A	−60.0 (9)	Fe2B—C21B—C22B—C23B	−59.0 (9)
Fe2A—C21A—C25A—C24A	59.0 (9)	Fe2B—C21B—C25B—C24B	60.4 (9)
Fe2A—C22A—C23A—C24A	−58.1 (9)	Fe2B—C22B—C23B—C24B	−60.1 (9)
Fe2A—C23A—C24A—C25A	−59.4 (9)	Fe2B—C23B—C24B—C25B	−57.5 (9)

Fe2A—C24A—C25A—C21A	−58.7 (9)	Fe2B—C24B—C25B—C21B	−59.8 (9)
Fe3A—C28A—C29A—C30A	−60.1 (8)	Fe3B—C28B—C29B—C30B	−59.1 (9)
Fe3A—C28A—C32A—C31A	59.6 (9)	Fe3B—C28B—C32B—C31B	58.5 (9)
Fe3A—C29A—C30A—C31A	−58.6 (9)	Fe3B—C29B—C30B—C31B	−58.4 (9)
Fe3A—C30A—C31A—C32A	−59.3 (9)	Fe3B—C30B—C31B—C32B	−59.4 (9)
Fe3A—C31A—C32A—C28A	−59.4 (8)	Fe3B—C31B—C32B—C28B	−58.5 (8)
Fe3A—C33A—C34A—C35A	−59.6 (11)	Fe3B—C33B—C34B—C35B	−60.0 (9)
Fe3A—C33A—C37A—C36A	59.5 (9)	Fe3B—C33B—C37B—C36B	60.2 (9)
Fe3A—C34A—C35A—C36A	−59.5 (9)	Fe3B—C34B—C35B—C36B	−59.7 (9)
Fe3A—C35A—C36A—C37A	−59.0 (9)	Fe3B—C35B—C36B—C37B	−58.6 (9)
Fe3A—C36A—C37A—C33A	−59.0 (9)	Fe3B—C36B—C37B—C33B	−60.5 (9)
N1A—C3A—C4A—C5A	−179.3 (10)	N1B—C3B—C4B—C5B	70.4 (13)
N1A—N2A—N3A—C1A	−0.2 (14)	N1B—N2B—N3B—C1B	−3.4 (13)
N3A—C1A—C2A—C26A	−174.7 (14)	N3B—C1B—C2B—C26B	−180.0 (13)
N3A—C1A—C2A—N1A	0.5 (13)	N3B—C1B—C2B—N1B	−0.5 (13)
N3A—C1A—C16A—C17A	12.1 (19)	N3B—C1B—C16B—C17B	−4.6 (19)
N3A—C1A—C16A—C20A	−169.9 (12)	N3B—C1B—C16B—C20B	177.9 (12)
N3A—C1A—C16A—Fe2A	−79.4 (15)	N3B—C1B—C16B—Fe2B	−91.6 (13)
O1A—C5A—C6A—C7A	−176.4 (15)	O1B—C5B—C6B—C7B	−2.0 (19)
O1A—C5A—C6A—C10A	8 (2)	O1B—C5B—C6B—C10B	−170.8 (12)
O1A—C5A—C6A—Fe1A	93.5 (17)	O1B—C5B—C6B—Fe1B	−86.5 (13)

Table S8. Bond lengths for **2c** [Å].

C1—C2	1.379 (8)	C14—C15	1.430 (9)
C1—C16	1.462 (8)	C14—Fe1	2.045 (6)
C1—N3	1.363 (7)	C15—Fe1	2.052 (5)
C2—N1	1.350 (7)	C16—C17	1.425 (8)
C3—C4	1.520 (8)	C16—C20	1.439 (8)
C3—N1	1.468 (7)	C16—Fe2	2.049 (6)
C4—C5	1.514 (8)	C17—C18	1.427 (8)
C5—C6	1.473 (8)	C17—Fe2	2.038 (6)
C5—O1	1.219 (7)	C18—C19	1.430 (9)
C6—C7	1.444 (8)	C18—Fe2	2.043 (6)
C6—C10	1.433 (8)	C19—C20	1.430 (8)
C6—Fe1	2.036 (5)	C19—Fe2	2.051 (6)
C7—C8	1.427 (9)	C20—Fe2	2.051 (6)
C7—Fe1	2.039 (5)	C21—C22	1.424 (9)
C8—C9	1.427 (9)	C21—C25	1.422 (9)
C8—Fe1	2.056 (5)	C21—Fe2	2.041 (6)
C9—C10	1.421 (9)	C22—C23	1.426 (8)
C9—Fe1	2.063 (6)	C22—Fe2	2.049 (6)
C10—Fe1	2.050 (5)	C23—C24	1.418 (9)
C11—C12	1.421 (9)	C23—Fe2	2.055 (6)
C11—C15	1.425 (9)	C24—C25	1.436 (8)
C11—Fe1	2.059 (6)	C24—Fe2	2.054 (6)
C12—C13	1.426 (9)	C25—Fe2	2.049 (6)
C12—Fe1	2.065 (5)	N1—N2	1.340 (7)
C13—C14	1.434 (9)	N2—N3	1.318 (7)
C13—Fe1	2.053 (5)		

Table S9. Values of valence angles for **2c** [°].

C2—C1—C16	131.5 (5)	C7—Fe1—C9	68.9 (2)
N3—C1—C2	108.0 (5)	C7—Fe1—C10	69.2 (2)
N3—C1—C16	120.4 (5)	C7—Fe1—C11	122.7 (2)
N1—C2—C1	104.5 (5)	C7—Fe1—C12	159.5 (3)
N1—C3—C4	111.1 (4)	C7—Fe1—C13	157.7 (2)
C5—C4—C3	113.0 (5)	C7—Fe1—C14	121.0 (2)
C6—C5—C4	117.8 (5)	C7—Fe1—C15	105.9 (2)
O1—C5—C4	121.1 (5)	C8—Fe1—C9	40.5 (2)
O1—C5—C6	121.0 (5)	C8—Fe1—C11	158.0 (2)
C5—C6—Fe1	121.2 (4)	C8—Fe1—C12	159.2 (3)
C7—C6—C5	127.8 (5)	C9—Fe1—C12	124.1 (2)
C7—C6—Fe1	69.3 (3)	C10—Fe1—C8	68.2 (2)
C10—C6—C5	124.3 (5)	C10—Fe1—C9	40.4 (2)
C10—C6—C7	107.7 (5)	C10—Fe1—C11	125.1 (2)
C10—C6—Fe1	70.0 (3)	C10—Fe1—C12	109.4 (2)
C6—C7—Fe1	69.2 (3)	C10—Fe1—C13	122.9 (2)
C8—C7—C6	107.3 (5)	C10—Fe1—C15	160.5 (2)
C8—C7—Fe1	70.2 (3)	C11—Fe1—C9	160.7 (2)
C7—C8—Fe1	68.9 (3)	C11—Fe1—C12	40.3 (2)
C9—C8—C7	108.7 (5)	C13—Fe1—C8	122.0 (2)
C9—C8—Fe1	70.0 (3)	C13—Fe1—C9	107.1 (2)
C8—C9—Fe1	69.5 (3)	C13—Fe1—C11	68.2 (2)
C10—C9—C8	107.9 (5)	C13—Fe1—C12	40.5 (2)
C10—C9—Fe1	69.3 (3)	C14—Fe1—C8	105.8 (2)
C6—C10—Fe1	68.9 (3)	C14—Fe1—C9	121.4 (3)
C9—C10—C6	108.4 (5)	C14—Fe1—C10	158.0 (2)
C9—C10—Fe1	70.3 (3)	C14—Fe1—C11	68.2 (2)
C12—C11—C15	108.6 (5)	C14—Fe1—C12	68.3 (2)
C12—C11—Fe1	70.1 (3)	C14—Fe1—C13	41.0 (2)
C15—C11—Fe1	69.5 (3)	C14—Fe1—C15	40.9 (3)
C11—C12—C13	108.2 (5)	C15—Fe1—C8	121.2 (2)
C11—C12—Fe1	69.6 (3)	C15—Fe1—C9	157.2 (2)
C13—C12—Fe1	69.3 (3)	C15—Fe1—C11	40.5 (2)
C12—C13—C14	107.5 (5)	C15—Fe1—C12	68.3 (2)
C12—C13—Fe1	70.2 (3)	C15—Fe1—C13	68.8 (2)

C14—C13—Fe1	69.2 (3)	C16—Fe2—C19	69.1 (2)
C13—C14—Fe1	69.9 (3)	C16—Fe2—C20	41.1 (2)
C15—C14—C13	108.2 (5)	C16—Fe2—C22	120.1 (2)
C15—C14—Fe1	69.9 (3)	C16—Fe2—C23	156.8 (3)
C11—C15—C14	107.5 (5)	C16—Fe2—C24	160.0 (2)
C11—C15—Fe1	70.0 (3)	C16—Fe2—C25	122.0 (2)
C14—C15—Fe1	69.3 (3)	C17—Fe2—C16	40.8 (2)
C1—C16—Fe2	124.5 (4)	C17—Fe2—C18	40.9 (2)
C17—C16—C1	128.0 (5)	C17—Fe2—C19	68.9 (2)
C17—C16—C20	107.4 (5)	C17—Fe2—C20	68.7 (2)
C17—C16—Fe2	69.2 (3)	C17—Fe2—C21	119.0 (2)
C20—C16—C1	124.6 (5)	C17—Fe2—C22	154.8 (2)
C20—C16—Fe2	69.5 (3)	C17—Fe2—C23	162.0 (2)
C16—C17—C18	108.5 (5)	C17—Fe2—C24	124.2 (3)
C16—C17—Fe2	70.0 (3)	C17—Fe2—C25	105.5 (2)
C18—C17—Fe2	69.7 (4)	C18—Fe2—C16	68.9 (2)
C17—C18—C19	108.2 (5)	C18—Fe2—C19	40.9 (2)
C17—C18—Fe2	69.4 (3)	C18—Fe2—C20	68.6 (2)
C19—C18—Fe2	69.8 (3)	C18—Fe2—C22	163.1 (2)
C18—C19—Fe2	69.2 (3)	C18—Fe2—C23	126.2 (2)
C20—C19—C18	107.6 (5)	C18—Fe2—C24	108.4 (2)
C20—C19—Fe2	69.6 (3)	C18—Fe2—C25	120.5 (2)
C16—C20—Fe2	69.4 (3)	C19—Fe2—C20	40.8 (2)
C19—C20—C16	108.3 (5)	C19—Fe2—C23	109.5 (2)
C19—C20—Fe2	69.6 (3)	C19—Fe2—C24	122.7 (2)
C22—C21—Fe2	69.9 (3)	C20—Fe2—C23	122.7 (2)
C25—C21—C22	108.6 (5)	C20—Fe2—C24	158.0 (2)
C25—C21—Fe2	70.0 (3)	C21—Fe2—C16	105.3 (2)
C21—C22—C23	107.7 (5)	C21—Fe2—C18	155.1 (2)
C21—C22—Fe2	69.3 (3)	C21—Fe2—C19	161.6 (2)
C23—C22—Fe2	69.9 (3)	C21—Fe2—C20	123.8 (2)
C22—C23—Fe2	69.5 (3)	C21—Fe2—C22	40.7 (2)
C24—C23—C22	108.2 (5)	C21—Fe2—C23	68.4 (2)
C24—C23—Fe2	69.8 (3)	C21—Fe2—C24	68.4 (2)

C23—C24—C25	108.2 (5)	C21—Fe2—C25	40.7 (2)
C23—C24—Fe2	69.9 (3)	C22—Fe2—C19	125.7 (2)
C25—C24—Fe2	69.4 (3)	C22—Fe2—C20	107.9 (2)
C21—C25—C24	107.3 (5)	C22—Fe2—C23	40.7 (2)
C21—C25—Fe2	69.3 (3)	C22—Fe2—C24	68.3 (2)
C24—C25—Fe2	69.7 (3)	C22—Fe2—C25	68.7 (2)
C6—Fe1—C7	41.5 (2)	C24—Fe2—C23	40.4 (3)
C6—Fe1—C8	68.8 (2)	C25—Fe2—C19	157.1 (2)
C6—Fe1—C9	68.8 (2)	C25—Fe2—C20	159.7 (2)
C6—Fe1—C10	41.0 (2)	C25—Fe2—C23	68.6 (2)
C6—Fe1—C11	108.6 (2)	C25—Fe2—C24	41.0 (2)
C6—Fe1—C12	123.9 (2)	C2—N1—C3	129.5 (5)
C6—Fe1—C13	159.4 (2)	N2—N1—C2	111.2 (4)
C6—Fe1—C14	158.6 (2)	N2—N1—C3	119.1 (5)
C6—Fe1—C15	122.9 (2)	N3—N2—N1	107.3 (5)
C7—Fe1—C8	40.8 (2)	N2—N3—C1	108.9 (5)

Table S10. Values of torsion angles for **2c** [°].

C1—C2—N1—C3	176.2 (5)	C17—C16—C20—C19	−0.3 (7)
C1—C2—N1—N2	0.5 (6)	C17—C16—C20—Fe2	−59.1 (4)
C1—C16—C17—C18	−177.4 (6)	C17—C18—C19—C20	−0.4 (7)
C1—C16—C17—Fe2	−118.2 (6)	C17—C18—C19—Fe2	59.0 (4)
C1—C16—C20—C19	177.3 (5)	C18—C19—C20—C16	0.4 (7)
C1—C16—C20—Fe2	118.5 (6)	C18—C19—C20—Fe2	59.1 (4)
C2—C1—C16—C17	12.4 (11)	C20—C16—C17—C18	0.1 (7)
C2—C1—C16—C20	−164.7 (6)	C20—C16—C17—Fe2	59.3 (4)
C2—C1—C16—Fe2	−77.0 (8)	C21—C22—C23—C24	0.0 (7)
C2—C1—N3—N2	0.0 (7)	C21—C22—C23—Fe2	59.2 (4)
C2—N1—N2—N3	−0.5 (6)	C22—C21—C25—C24	0.2 (7)
C3—C4—C5—C6	167.9 (4)	C22—C21—C25—Fe2	−59.4 (4)
C3—C4—C5—O1	−14.2 (7)	C22—C23—C24—C25	0.1 (7)
C3—N1—N2—N3	−176.7 (5)	C22—C23—C24—Fe2	59.0 (4)
C4—C3—N1—C2	120.5 (6)	C23—C24—C25—C21	−0.2 (7)
C4—C3—N1—N2	−64.2 (7)	C23—C24—C25—Fe2	59.2 (4)
C4—C5—C6—C7	−0.2 (8)	C25—C21—C22—C23	−0.1 (7)
C4—C5—C6—C10	−173.1 (5)	C25—C21—C22—Fe2	59.5 (4)
C4—C5—C6—Fe1	−87.2 (5)	Fe1—C6—C7—C8	−60.2 (4)
C5—C6—C7—C8	−174.3 (5)	Fe1—C6—C10—C9	59.4 (4)
C5—C6—C7—Fe1	−114.1 (5)	Fe1—C7—C8—C9	−58.8 (4)
C5—C6—C10—C9	174.2 (5)	Fe1—C8—C9—C10	−58.9 (4)
C5—C6—C10—Fe1	114.8 (5)	Fe1—C9—C10—C6	−58.5 (4)
C6—C7—C8—C9	0.7 (6)	Fe1—C11—C12—C13	−58.8 (4)
C6—C7—C8—Fe1	59.5 (4)	Fe1—C11—C15—C14	59.4 (4)
C7—C6—C10—C9	0.0 (6)	Fe1—C12—C13—C14	−59.3 (4)
C7—C6—C10—Fe1	−59.3 (4)	Fe1—C13—C14—C15	−59.5 (4)
C7—C8—C9—C10	−0.7 (6)	Fe1—C14—C15—C11	−59.8 (4)
C7—C8—C9—Fe1	58.2 (4)	Fe2—C16—C17—C18	−59.2 (4)
C8—C9—C10—C6	0.4 (6)	Fe2—C16—C20—C19	58.8 (4)
C8—C9—C10—Fe1	59.0 (4)	Fe2—C17—C18—C19	−59.2 (4)
C10—C6—C7—C8	−0.4 (6)	Fe2—C18—C19—C20	−59.3 (4)
C10—C6—C7—Fe1	59.8 (4)	Fe2—C19—C20—C16	−58.7 (4)

C11—C12—C13—C14	−0.4 (6)	Fe2—C21—C22—C23	−59.6 (4)
C11—C12—C13—Fe1	59.0 (4)	Fe2—C21—C25—C24	59.6 (4)
C12—C11—C15—C14	0.1 (6)	Fe2—C22—C23—C24	−59.2 (4)
C12—C11—C15—Fe1	−59.3 (4)	Fe2—C23—C24—C25	−58.9 (4)
C12—C13—C14—C15	0.4 (6)	Fe2—C24—C25—C21	−59.4 (4)
C12—C13—C14—Fe1	60.0 (4)	N1—C3—C4—C5	−78.4 (6)
C13—C14—C15—C11	−0.3 (6)	N1—N2—N3—C1	0.3 (6)
C13—C14—C15—Fe1	59.5 (4)	N3—C1—C2—N1	−0.3 (6)
C15—C11—C12—C13	0.2 (6)	N3—C1—C16—C17	−168.7 (6)
C15—C11—C12—Fe1	59.0 (4)	N3—C1—C16—C20	14.3 (9)
C16—C1—C2—N1	178.7 (6)	N3—C1—C16—Fe2	101.9 (6)
C16—C1—N3—N2	−179.1 (5)	O1—C5—C6—C7	−178.2 (5)
C16—C17—C18—C19	0.2 (7)	O1—C5—C6—C10	8.9 (8)
C16—C17—C18—Fe2	59.4 (4)	O1—C5—C6—Fe1	94.8 (6)

Table S11. Values of angle of relative rotation of the cyclopentadienyl rings (τ) within individual ferrocene moieties in the crystals of investigated compounds.

Compound	Ferrocene*	$\|\tau\|$ [°]
**1a**	Fe1A	19.77
	Fe2A	10.27
	Fe1B	29.96
	Fe2B	0.91
**2a**	Fe1A	25.34
	Fe2A	1.83
	Fe3A	2.23
	Fe1B	11.34
	Fe2B	5.44
	Fe3B	8.16
**2c**	Fe1	0.91
	Fe2	4.00

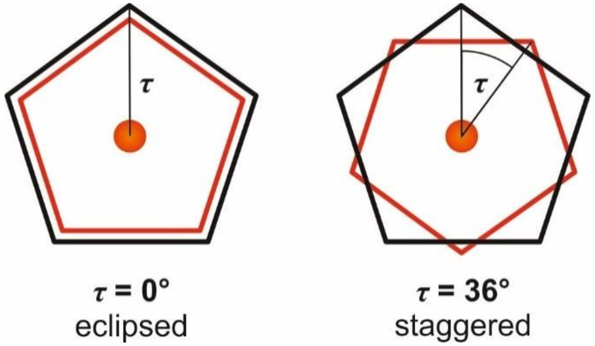

*The ferrocene moieties are labelled after the Fe atoms included in their composition.

Table S12. Cremer & Pople and pseudorotation parameters calculated for the pentafuranose rings present in **1a**.

Parameter	Molecule A	Molecule B
***Q*(2) [Å]**	0.273(10)	0.349(10)
***Φ* [º]**	82.0(19)	83.1(15)
***P* [º]**	170.3(12)	171.8(9)
***τ* [º]**	28.0(6)	35.1(6)

The meaning of Q, Φ, P and *τ* is in: D. Cremer & J.A. Pople, J.Am.Chem.Soc., 97, (1975), 1354–1358.

Table S13. Experimental (IR_{exp}) and BLYP/6-31+G(d)/LanL2DZ calculated (IR_{calc})[a] carbon-carbon triple bond stretch ($n_{C≡C}$ in cm^{-1}). Bond lengths (in Å) calculated at the same level of theory.

Species	IR_{exp} ($\nu_{C≡C}$)	IR_{calc} ($\nu_{C≡C}$)[b]	$r_{C≡C}$[b]
**1a**	2214	2211	1.232
**1a^{+}**	2210	2198	1.233
**1a^{2+}**	2216	2212	1.229

[a] No scaling factor employed. [b] The calculated frequency/bond length corresponds to the averaged value for different set of conformers (Boltzmann population weights).

$$V_{ab} = 2.06 \text{ x } 10^{-2} \text{ x } (\varepsilon_{max} \nu_{max} \Delta\nu_{1/2})^{1/2} \text{ x } r_{ab}^{-1}$$

where ν_{max} = the energy, ε_{max} = the molar extinction coefficient, $\Delta\nu_{1/2}$ = the full width at half-height of the IVCT band, r_{ab} = the electron transfer distance).

Details on DFT Calculations. Density functional theory was applied to study the electronic structure properties of compounds **1a**, **1c**, **2a**, and **2c** in more details. The available experimental data (solid state structures and redox properties) were reproduced and additional insights were provided.Two crystallographically independent molecules (**A** and **B**) were observed in case of **1a** and **2a**. The experimental coordinates of these conformers for **1a** and **2a** in crystal lattices were used as starting coordinates in geometry optimization at the BLYP/6-31+G(d)/LanL2DZ level of theory. The calculated relative energy difference in the gas phase between the two conformers (**A** and **B**) was less than 2.5 kJ/mol in each case. The global minimum of **1a** (and **1b**) in dichloromethane, however, is different from the experimental solid state structure, which may indicate a significant geometry change upon dissolving it in the respective solvent. A full conformational search was performed for **1a** and **2a** to probe their structural flexibility. A hundred of conformers were located, but only low-energy structures (the most stable minima within the range of 5 kJ/mol) were considered for further calculations (e.g. vibrational frequencies or HOMO energies, see below). In addition, conformational analysis for **1c** and **2c**, derivatives without the Fc-C≡C- unit, was carried out for comparative reasons. The experimental (and calculated) redox potentials for **1c** and **2c** ($E^{o\prime}$ = 60 and 20 mV, resp.) are similar to a series of ferrocenyl-triazole derivatives measured earlier.[1,2,3] $E^{o\prime}$ values for different triazole-tethered ferrocene conjugates (benzyl, quinoline, phenantrene, or ferrocene) vary between 22 – 50 mV, which suggests that the *N*1-substituent at the triazole ring (the nucleoside in **1c** and Fc-CO-CH_2CH_2- unit in **2c**) may induce a slight effect on the ferrocenyl redox potential. It also indicates that 1,3-substituted triazole spacer participates in electronic communication between the two parts of the molecule.The introduction of the Fc-C≡C- unit at the C5-position (**1a** and **2a**) induces additional anodic shift of 20-25 mV, i.e. towards higher potentials for the first oxidation. The computational evidence that all compounds (**1a**, **2a**, **1c**, and **2b**) undergo the initial oxidation at the ferrocenyl moiety at the C4-position comes from the FMO analysis. In each case the HOMO orbital is localized at the ferrocene ring, which is directly bonded to the heterocyclic core (Figure S15). The calculated energies of HOMO orbitals (E_{HOMO}) in **1a**, **1c**, **2a**, and **2c** nicely fit (linear correlation coefficient r = 0.99) to experimental redox potentials (E^{o}) measured in dichloromethane (Figure S17). It is in agreement with reported observation that experimental redox potentials for a series of ferrocene derivatives correlate well with calculated HOMO energies.[4] The higher is the HOMO energy, the easier is the oxidation of the ferrocene moiety, i.e. the lower is the $E^{o\prime}$ value. The second and the third redox event, however, may not be easily predicted by using calculated energy values of the frontier molecular orbitals. Instead, vertical ionization energies (*IE*) were found to be associated with experimental redox potentials. The *IE* was calculated as the electronic energy difference between oxidized and reduced forms involved in the

respective redox event. A clear trend was observed when calculated *IE* values were correlated to the first, second, and the third redox potential (Figure S17). In the case of dicationic species (**1a**$^{2+}$, **2a**$^{2+}$, and **2c**$^{2+}$) the triplet ground state was calculated ca. 180 kJ/mol lower than the singlet state, while the triply oxidized **2a**$^{3+}$ exists as a quartet. The geometries of these open-shell species were not optimized, i.e. a required number of electrons were removed from the neutral molecule to obtain the desired multiplet. This is why data points in the graph are somewhat scattered.

References: 1. Romero, T.; Orenes, R. A.; Tárraga, A.; Molina, P. *Organometallics* **2013**, *32*, 5740-5753.
2. Djaković, S.; Maračič, S.; Lapić, J.; Kovalski, E.; Hildebrandt, A.; Lang, H. Vrček. V.; Raić-Malić, S.; Cetina, M. *Struct. Chem.* **2021** doi 10.1007/s11224-021-01801-2
3. Verschoor-Kirss, M.; Kreisz, J.; Feighery, W.; Reiff, W. M.; Frommen, C.M.; Kirss, R. U. *J. Organomet. Chem.* **2009**, *694*, 3262-3269.
4. Toma, M.; Kuvek, T. Vrček, V. *J. Phys. Chem. A* **2020**, *124*, 8029-8039.

BLYP/6-31+G(d)/LanL2DZ optimized geometries and corresponding Gibbs free energies in Hartrees (at 298 K, in parentheses).

Only the most stable conformer included.

1a (G = -2134.525022)

Fe,0,2.3402117911,3.1016535509,3.3998911652
Fe,0,-4.1636115555,0.8259475467,-0.6122768471
O,0,-0.6847536063,-1.1049830453,-3.3131460227
O,0,1.4311866705,0.253883897,-4.9474895299
N,0,1.8258860499,1.4005344885,-1.496997846
O,0,-1.3355541135,1.2940520709,-4.8010042075
C,0,2.1511180002,1.2706084328,4.3560296657
O,0,-2.4082563056,2.9695228282,-8.9698390731
N,0,3.5187424244,1.8448292017,-0.2071117229
N,0,3.1483853614,1.7659778176,-1.474231654
N,0,0.4165256993,2.027720749,-6.1624665269
C,0,2.4554978881,1.5277079875,0.6138255397
C,0,2.5879583881,1.5042431936,2.0682505956
N,0,-1.8089294161,2.1502840456,-6.8927271162
C,0,3.5426973508,1.6129469138,4.2024676111
C,0,3.8125044453,1.7698946191,2.7990397945
C,0,1.3891296095,4.5231759635,4.5820518188
C,0,1.3396666964,1.2273786903,-0.2028950127
C,0,1.5681369204,-0.0702875072,-3.532231752
C,0,1.5572487447,1.2129604357,3.0464914169
C,0,2.7814690707,4.8579262224,4.4196851026
C,0,0.0206928748,0.816724498,0.055468381
C,0,1.1077887317,1.2089854706,-2.76385937
C,0,3.039839912,5.020339868,3.0105491692
C,0,-0.9387349914,1.7828340315,-5.8733199154
C,0,1.334533973,2.3654121385,-3.7738887458
C,0,0.7261397255,-1.3310437641,-3.2650013604
C,0,1.806379064,4.788139994,2.3034462867
C,0,0.7863280502,4.4787553587,3.2731099455
C,0,1.4606746496,1.6624477757,-5.1578268965
C,0,-4.0466482516,1.8480081818,-2.421969314
C,0,-1.5036084866,2.7046991213,-8.1744283202
C,0,-5.4058979007,1.4430050298,-2.1608090538
C,0,-1.1475011896,0.4391499203,0.1535406396
C,0,0.8025436957,2.5446690262,-7.4025687731
C,0,-3.6280006157,2.7018301931,-1.339065457
C,0,-0.063207243,2.8933998105,-8.3986914053
C,0,-5.8225163642,2.0430853156,-0.9181972961
C,0,-4.7212652722,2.821709185,-0.4094667015
C,0,-2.4678372868,-0.0723086438,0.2036877859
C,0,-3.0665541506,-0.9219461838,-0.8202871716
C,0,-4.4064795525,-1.2303887783,-0.4085050195
C,0,-4.6638538088,-0.5681885496,0.8460365747
C,0,-3.482522718,0.1583125603,1.2238862053
C,0,0.3788883462,3.4575686043,-9.7290776087
H,0,-0.8718089581,-0.4744369972,-4.0490464871
H,0,1.6298258987,1.1107482774,5.2991651584
H,0,-2.7999753143,1.9858323909,-6.7011708451
H,0,4.2591507461,1.7609226455,5.0097459305
H,0,4.7617552046,2.040673697,2.34206887
H,0,0.8869181809,4.3137339574,5.526047526
H,0,2.632627708,-0.2750180119,-3.3094965012
H,0,0.5166805416,0.9911276113,2.819119953
H,0,3.5164956087,4.9438695182,5.2194372783
H,0,0.0461373899,1.1066044323,-2.5106500656
H,0,4.0036384193,5.2483146231,2.5570277751

H,0,0.5146295308,3.0960078747,-3.7543085715
H,0,2.2746855986,2.8838350406,-3.5381028095
H,0,1.0401330077,-2.1067010541,-3.9941141548
H,0,0.954789918,-1.7086635685,-2.254068846
H,0,1.6791226612,4.8008922554,1.2215712419
H,0,-0.2516031135,4.230562055,3.0540471328
H,0,2.4092645721,1.9452220837,-5.6469826077
H,0,-3.4284190517,1.542841925,-3.2654768462
H,0,-6.0036805158,0.7722804364,-2.7773522507
H,0,1.8821512547,2.6591116988,-7.5224604303
H,0,-2.6364924378,3.1366858781,-1.217070393
H,0,-6.7895765008,1.9090425511,-0.4344259526
H,0,-4.7097007552,3.3809101826,0.5254703499
H,0,-2.5514735553,-1.241908432,-1.7249446356
H,0,-5.1197583026,-1.8345463494,-0.9681087058
H,0,-5.602193252,-0.5871953788,1.3991845959
H,0,-3.3519804353,0.7715360853,2.1140538633
H,0,0.0412331902,2.8141663501,-10.5594257503
H,0,1.4748797939,3.5524928212,-9.7815007837
H,0,-0.069498337,4.4508771346,-9.9017110166

1a$^+$ (G = -2134.323369)

Fe,0,5.4587520354,-1.2521793327,-0.0031170243
Fe,0,-0.5077177847,4.317148819,0.01815407
O,0,-2.0587802726,0.208497412,2.2013356526
O,0,-2.954059692,-2.3723310752,1.5635919154
N,0,0.2164057102,-1.9935224712,-0.2365193844
O,0,-5.4268414047,-4.8485068883,0.0624845185
C,0,5.8796082202,-1.6526383454,-1.9986296475
O,0,-8.4615022919,-1.5314564261,-1.0326192909
N,0,2.048712751,-3.1441898279,-0.4677641053
N,0,0.7764579906,-3.2434986532,-0.1453477206
N,0,-4.6543896389,-2.6427202489,-0.0596593438
C,0,2.3519670249,-1.8319252273,-0.7653780368
C,0,3.6815255523,-1.4552358813,-1.2222043137
N,0,-6.909831348,-3.1546741893,-0.4854818118
C,0,5.5931343717,-0.2513133094,-1.8270021505
C,0,4.2484979457,-0.1299706103,-1.3308583432
C,0,6.0389236249,-2.564484395,1.5540130357
C,0,1.1704600694,-1.0573078631,-0.6182956813
C,0,-1.5384002752,-2.1142715877,1.5786133301
C,0,4.7159008536,-2.3964790937,-1.6006488275
C,0,7.1622418358,-1.7969102825,1.0776042419
C,0,0.8969882782,0.3043158689,-0.8197633113
C,0,-1.2042326772,-1.7861414202,0.0890040912
C,0,6.8693798112,-0.402136537,1.2927402903
C,0,-5.655800492,-3.6567119419,-0.1393920499
C,0,-2.1772179763,-2.699988293,-0.7049116038
C,0,-1.3012296105,-0.9600189643,2.5658277597
C,0,5.5671752439,-0.3133826723,1.902099102
C,0,5.0621808377,-1.6466372328,2.0671262303
C,0,-3.3048156896,-3.0539136747,0.3235477624
C,0,-2.2717703149,4.3061592137,1.1954212007
C,0,-7.2973039392,-1.8065466126,-0.7361672823
C,0,-1.2823082117,3.4729847561,1.8169011767
C,0,0.6360825484,1.4953526053,-1.0012884103
C,0,-4.9567270102,-1.2947637578,-0.2686206692
C,0,-1.7368532619,5.637743137,1.083250652
C,0,-6.1958508201,-0.8335534109,-0.5998648495
C,0,-0.1255083137,4.2863300385,2.0841082718
C,0,-0.4071674743,5.6276692199,1.6370156143
C,0,0.3636990772,2.8545198278,-1.2831267738
C,0,1.2623769181,3.9779764493,-1.0720076757
C,0,0.6095216196,5.1691694046,-1.5322536916
C,0,-0.7052169417,4.8122883765,-1.9976885951
C,0,-0.8756252001,3.3970266118,-1.8235715031

C,0,-6.5170615631,0.6251036097,-0.8267361927
H,0,-3.0014093375,-0.0645805306,2.2496611858
H,0,6.8183775017,-2.0784750106,-2.3495360502
H,0,-7.6516093618,-3.8549589048,-0.5606163386
H,0,6.2770499971,0.5731883507,-2.0225744526
H,0,3.7367161092,0.7970996977,-1.0817340212
H,0,5.93693616,-3.6478638537,1.5115593414
H,0,-0.9704443488,-3.0119972898,1.8938100433
H,0,4.6007968038,-3.4781826813,-1.5945694708
H,0,8.0673586257,-2.2000075372,0.6256219567
H,0,-1.4055642297,-0.7207637457,-0.088983939
H,0,7.5143365581,0.4360936028,1.0331405604
H,0,-2.5799608633,-2.204543961,-1.6002766213
H,0,-1.6609241303,-3.6166017336,-1.0239262418
H,0,-1.5533212641,-1.2954888646,3.5891753805
H,0,-0.2403136775,-0.6634595709,2.5531709948
H,0,5.0494320993,0.605581906,2.173623358
H,0,4.0823818941,-1.9168067109,2.4591702734
H,0,-3.3710101924,-4.1366534627,0.4960981691
H,0,-3.2487613287,3.9806458772,0.8404139666
H,0,-1.3847479278,2.4031646674,2.0056684647
H,0,-4.1129320853,-0.6162670457,-0.125513744
H,0,-2.2402654268,6.4957791799,0.6397789818
H,0,0.8041073809,3.9469984863,2.5391123437
H,0,0.2697102213,6.4792132279,1.6919732435
H,0,2.2612002193,3.9102453433,-0.6445064579
H,0,1.0292737346,6.1736719921,-1.5080701159
H,0,-1.4529905185,5.4981551654,-2.392896312
H,0,-1.7572751334,2.811489742,-2.0769417579
H,0,-6.9161277869,0.7857634499,-1.8426942894
H,0,-5.6241636944,1.2583822752,-0.6953575269
H,0,-7.2993491137,0.9690858152,-0.1289215499

1a^{2+} (G = -2134.028697), triplet state

Fe,0,5.8388148217,-0.982051851,-0.4979334144
Fe,0,-1.6825103046,4.1244708214,-0.0300819034
O,0,-2.2792955395,0.2494859459,2.3936202543
O,0,-2.7343856082,-2.6203140194,1.7685395108
N,0,0.651078314,-1.6432968901,0.754929663
O,0,-3.5885763597,-0.4902111589,-0.0775338812
C,0,5.9482926591,0.8917217863,0.4390002424
O,0,-7.5990671021,-1.8230457376,-1.9375181543
N,0,2.6334299984,-2.4464168938,1.1563874623
N,0,1.3564031283,-2.7603611439,1.1254653936
N,0,-4.0163990856,-2.7880571292,-0.2258087448
C,0,2.79302392,-1.1223552672,0.8075161981
C,0,4.1127873603,-0.5048867097,0.8242697129
N,0,-5.5750386488,-1.222697072,-1.0008975554
C,0,6.371121467,-0.1576502839,1.3343029755
C,0,5.2517168595,-1.0373791769,1.5426690954
C,0,7.1179188216,-0.9612081493,-2.1767052622
C,0,1.5112708362,-0.5751547923,0.540232061
C,0,-1.4421734189,-2.0558286163,2.1033285338
C,0,4.5668438857,0.6637091823,0.1111040306
C,0,7.5412491469,-1.9617590764,-1.2266440293
C,0,1.0775036471,0.7060943344,0.1535752226
C,0,-0.8238106872,-1.6578732153,0.7229880568
C,0,6.4797121406,-2.9316404793,-1.105660543
C,0,-4.3456690255,-1.4292561817,-0.4081322275
C,0,-1.4306828945,-2.6852478756,-0.2658506291
C,0,-1.6914806921,-0.877300118,3.0599915428
C,0,5.4170781703,-2.5361374794,-1.9833676314
C,0,5.8019692346,-1.3208959946,-2.6374391105
C,0,-2.7403341285,-3.159096844,0.4443628024
C,0,-2.53315214,4.6233487701,1.9151692723
C,0,-6.5482525134,-2.194007001,-1.4221916893

C,0,-3.1673194953,5.369047986,0.8637062487
C,0,0.6403492051,1.8065891465,-0.1828067664
C,0,-4.9183614035,-3.8018113124,-0.5807411874
C,0,-2.7147278337,3.2275690467,1.6520852166
C,0,-6.136962044,-3.5838800628,-1.1616972741
C,0,-3.7483535145,4.4269285491,-0.0610508406
C,0,-3.4598487928,3.0976184923,0.4261483753
C,0,0.1667661119,3.0600987244,-0.6429357854
C,0,0.4718041228,4.3650967076,-0.0912912179
C,0,-0.1980141778,5.3578892548,-0.8807443424
C,0,-0.9542714537,4.6813576086,-1.9061716282
C,0,-0.7560949915,3.267193794,-1.7452111553
C,0,-7.0898159692,-4.6892630234,-1.5423762204
H,0,-2.9972617378,-0.0919707734,1.8061572149
H,0,6.564045648,1.7116258529,0.0716876048
H,0,-5.8451311941,-0.2467238275,-1.1444334849
H,0,7.3601556821,-0.2678975675,1.7769567313
H,0,5.2325328111,-1.9357035544,2.1569519226
H,0,7.6928605253,-0.0919784275,-2.4935144771
H,0,-0.8131820705,-2.8221680619,2.5938320084
H,0,3.9639351675,1.2653396834,-0.5671466663
H,0,8.4947081341,-1.9862700142,-0.7005010374
H,0,-1.1403851805,-0.6394876212,0.4747453117
H,0,6.4780442672,-3.8037867085,-0.45286706
H,0,-1.6264771194,-2.2517022289,-1.2559868986
H,0,-0.7533048714,-3.5439396694,-0.386690747
H,0,-2.3266947509,-1.2361663044,3.8919168125
H,0,-0.7359260979,-0.5325808765,3.4883702891
H,0,4.4581595274,-3.0433491412,-2.0866771251
H,0,5.1972818501,-0.7565351809,-3.3465661965
H,0,-2.7667225053,-4.2594516004,0.4937074163
H,0,-1.9728641145,5.0497579534,2.7471521684
H,0,-3.1872948953,6.4545355505,0.7705757678
H,0,-4.571477945,-4.8121459665,-0.3558271283
H,0,-2.3303595287,2.3870902763,2.2338276369
H,0,-4.3039403615,4.67920413,-0.9636047432
H,0,-3.7452593237,2.1516184675,-0.0352507043
H,0,1.1044376659,4.5466654457,0.7763872772
H,0,-0.1447098852,6.4348303389,-0.7265974199
H,0,-1.566648183,5.1552796555,-2.6721600165
H,0,-1.1920673553,2.4762610926,-2.3531287554
H,0,-8.0561148664,-4.5615263738,-1.0254484086
H,0,-6.6824739184,-5.680383334,-1.2929792806
H,0,-7.3100153999,-4.6565108095,-2.6230556896

1c (G = -1549.382674)

Fe,0,1.5059669142,-5.4740466108,-1.4953597768
O,0,1.4145115478,2.8610304056,-2.0346038307
O,0,0.4641857458,3.0108992369,0.880229146
N,0,0.8794766849,-0.4761250426,-0.1424063291
O,0,-1.399542166,3.2346456823,-1.3965891421
C,0,3.1733323833,-5.2051057166,-2.6992115216
O,0,-4.7112322729,6.2092230717,-0.1684067411
N,0,1.7142066639,-2.2442665962,0.8069363279
N,0,1.1596033529,-1.0727087711,1.0675158418
N,0,-1.8698476144,3.4577084178,0.8835748004
C,0,1.8054288536,-2.419492305,-0.5649278623
C,0,2.4061948862,-3.6155082923,-1.159100049
N,0,-3.0459585075,4.7033507785,-0.7196259346
C,0,3.5716645673,-5.6328447739,-1.3819617993
C,0,3.0926572018,-4.664192373,-0.4325549069
C,0,-0.4245323255,-5.6898615399,-2.2388019215
C,0,1.2753183061,-1.2819098468,-1.1773280023
C,0,1.2951282054,1.9697731119,0.2890383469
C,0,2.4479099096,-3.9687687378,-2.5645991675
C,0,0.3100627398,-6.9211423733,-2.389761772

C,0,0.3005800261,0.8736032295,-0.2031155149
C,0,0.7248930629,-7.3549824255,-1.0796047794
C,0,-2.0500577043,3.7612651325,-0.4805964513
C,0,-0.943894463,1.0542262909,0.7036349267
C,0,2.1389360819,2.6130016495,-0.8297932504
C,0,0.2476949151,-6.3918421007,-0.1188951404
C,0,-0.4637691963,-5.3643497066,-0.8355149717
C,0,-0.8012405316,2.4940092752,1.2822206108
C,0,-3.8691071082,5.3955492663,0.2201749409
C,0,-2.6164934118,4.109409644,1.8689201908
C,0,-3.5881901385,5.0346812429,1.6174776353
C,0,-4.3890139672,5.7192864854,2.7008998508
H,0,0.5560554717,3.2826144444,-1.7902281188
H,0,-3.1962305641,4.935238916,-1.7042256792
H,0,3.3616048747,-5.7357217399,-3.6317583813
H,0,4.1133074894,-6.547070731,-1.142067292
H,0,3.2107633995,-4.6952064058,0.6484532103
H,0,-0.8552804038,-5.098770694,-3.0464161808
H,0,1.1612043355,-0.9851227555,-2.2155929076
H,0,1.9563885089,1.549963157,1.0703540359
H,0,1.999004415,-3.4010204003,-3.3791099419
H,0,0.5343251193,-7.4220574186,-3.3310198645
H,0,0.0565656497,1.0715027757,-1.2534768683
H,0,1.3203448141,-8.2399597905,-0.8574260097
H,0,-0.9104453705,0.3237014238,1.5246434547
H,0,-1.8838704178,0.9209709002,0.1510107773
H,0,2.5953912157,3.5370086029,-0.4173896093
H,0,2.9612167772,1.926495551,-1.0955444626
H,0,0.4208075278,-6.4178790228,0.9561852152
H,0,-0.9134033822,-4.4751780795,-0.3951983725
H,0,-0.8607329531,2.4716724589,2.3844681817
H,0,-2.3645233998,3.8137422245,2.8899073311
H,0,-4.0892897707,5.3727501681,3.7024878429
H,0,-5.4680641267,5.5286850707,2.5706829768
H,0,-4.2577138509,6.8138076356,2.6533629091

1c^{+} (G = -1549.170160)

Fe,0,5.2043921586,0.4509426629,0.2597827766
O,0,-2.0208121883,-2.5478871486,1.652426993
O,0,-3.4418111412,-1.5870242383,-1.0095957217
N,0,0.051682165,-0.5340838312,-0.607602771
O,0,-3.7204726861,-0.1835266635,1.5519062307
C,0,5.6029373219,-1.4726883618,0.9535334017
O,0,-7.9747755864,1.6045097604,1.6782989217
N,0,1.8580081783,-0.3662481208,-1.8113769635
N,0,0.5541356369,-0.2352133074,-1.8690279283
N,0,-4.7467110002,0.3324650056,-0.4855501267
C,0,2.225391839,-0.7541392079,-0.5296155191
C,0,3.6130553636,-1.0276789332,-0.1991780353
N,0,-5.8479803217,0.708594587,1.553255051
C,0,5.9167785423,-1.3674464579,-0.4497739947
C,0,4.7002745814,-1.0579707194,-1.1525516961
C,0,5.0169268233,2.1913278154,1.5101623632
C,0,1.0604725139,-0.8621029738,0.2424245378
C,0,-2.050109259,-1.920094742,-0.7659544354
C,0,4.1924880958,-1.2315999735,1.1086858037
C,0,6.3943525519,1.7974526858,1.3531168923
C,0,-1.3951497708,-0.5745537633,-0.3265256659
C,0,6.7166650657,1.8723234458,-0.050616497
C,0,-4.7017372119,0.2481058083,0.9247436593
C,0,-2.2289013577,0.5028199041,-1.0537510834
C,0,-1.980961336,-3.0325516163,0.3088076694
C,0,5.5352427748,2.3110642891,-0.7518315027
C,0,4.4967929311,2.5159428416,0.2146154205
C,0,-3.6062718779,-0.1958244768,-1.2870840484
C,0,-7.0432962483,1.2321861571,0.9655493838

C,0,-5.8870510122,0.8061859734,-1.1418977091
C,0,-7.0101725702,1.2582093775,-0.5069740469
C,0,-8.2275777461,1.7756497822,-1.2334043441
H,0,-2.8306282977,-1.9913823459,1.7489574945
H,0,-5.8370812371,0.6601740651,2.5753225679
H,0,6.3041858448,-1.690099143,1.7578930401
H,0,6.8996903496,-1.4933221759,-0.9015227898
H,0,4.5860783482,-0.8978533699,-2.2228128484
H,0,4.4634081269,2.2216878941,2.4479163679
H,0,0.8668354509,-1.1649788944,1.2681726789
H,0,-1.5937203222,-2.2763734216,-1.7103911575
H,0,3.6506908317,-1.220261565,2.0537049879
H,0,7.0704173123,1.4998148085,2.1533946583
H,0,-1.5002509327,-0.4822947811,0.7592655729
H,0,7.6802558494,1.6409728661,-0.502316132
H,0,-1.7757823592,0.747751838,-2.0254125354
H,0,-2.3240802025,1.427787458,-0.4684029306
H,0,-2.7927240747,-3.7557489677,0.095928524
H,0,-1.0230726837,-3.5700758813,0.2076311894
H,0,5.4405419216,2.4443815249,-1.8287330864
H,0,3.4694706292,2.8030267757,-0.0080348248
H,0,-3.9278142927,-0.0639136586,-2.3333817346
H,0,-5.8183077898,0.7936423896,-2.2318178503
H,0,-8.09453769,1.7361338493,-2.3253817894
H,0,-8.4426926057,2.8173798888,-0.9400601803
H,0,-9.1205044062,1.1867132864,-0.9631882378

2a (G = -2037.112359)

C,0,0.6061297368,22.3738007138,6.8682388195
C,0,-0.7020465215,22.8941302694,7.2245953489
C,0,-1.0137809396,23.9725516461,6.3330553636
C,0,0.082107434,24.1271111282,5.4085165421
C,0,1.0772557762,23.1395331252,5.7216717687
C,0,-0.8086408858,20.2106762845,4.6215388812
C,0,-0.4362637922,21.0725156239,3.5309468437
C,0,-1.4861842668,22.0422739714,3.3478706638
C,0,-2.5081834054,21.7800609855,4.3299595662
C,0,-2.0869680724,20.6497319042,5.1195264813
C,0,1.2575729519,21.2314237119,7.5483113991
C,0,2.6164341253,20.7480257297,6.9966984437
C,0,3.0767982323,19.4101348525,7.6054809753
C,0,1.6148987517,17.3165579849,7.9596823678
C,0,0.9435559308,16.5000878568,7.0169692184
C,0,1.6983373438,17.2857647347,9.3623867424
C,0,1.7693385103,17.3086016324,10.5910975636
C,0,1.8357452305,17.3190287624,12.0066918147
C,0,0.8173067529,16.8204810851,12.9234870027
C,0,1.2846791704,17.0210231602,14.2671511863
C,0,2.5738753273,17.6616094016,14.2067205428
C,0,2.9141944933,17.8609916334,12.8250049761
C,0,0.396184013,20.3803423897,12.0665056011
C,0,1.4411035848,20.8698737599,12.9283650155
C,0,1.0446355909,20.6375561645,14.2950660807
C,0,-0.2474719699,20.0000395205,14.2786049888
C,0,-0.6455134034,19.8399012159,12.9017536643
C,0,0.1112657339,15.3153990979,7.2143916833
C,0,-0.5804083978,14.6037660303,6.156610815
C,0,-1.2789158164,13.4921435309,6.7419177259
C,0,-1.0429964908,13.5132122785,8.1634904844
C,0,-0.1960838979,14.6385024564,8.4601894158
C,0,-2.6081659202,17.2447856045,7.7208174653
C,0,-2.9483814457,16.5070655355,8.9109543209
C,0,-3.7908476659,15.4025253614,8.5254410124
C,0,-3.969639512,15.4572792959,7.0963725188
C,0,-3.2374472158,16.5954885671,6.598234711
Fe,0,-0.7054040148,22.204741578,5.266106679

Fe,0,1.142782719,18.8357588614,13.2629025693
Fe,0,-1.9522077879,15.2776776397,7.5616258682
N,0,1.1929994898,17.0024437294,5.7581053206
N,0,1.9719802211,18.0720419158,5.849343668
N,0,2.2322607338,18.2819595102,7.1757404218
O,0,0.7531851536,20.697716526,8.5487826407
H,0,-1.3210301786,22.5081469747,8.031464474
H,0,-1.9371973956,24.550669528,6.3275647578
H,0,0.1308834778,24.8434407892,4.5889421227
H,0,2.0178271183,22.9946891625,5.1928792839
H,0,-0.2077902357,19.3907335569,5.0122319859
H,0,0.4879853089,21.011287056,2.9578025989
H,0,-1.4942073565,22.8491065134,2.6156342243
H,0,-3.4231473164,22.3547004408,4.4702822718
H,0,-2.6303212694,20.215709296,5.957756197
H,0,2.5753622873,20.6700707674,5.8990154912
H,0,3.3786417548,21.516744066,7.2298016896
H,0,4.117843507,19.2008185577,7.3083835925
H,0,3.0178501828,19.4408267494,8.7014847239
H,0,-0.1282564705,16.3729717509,12.6222704332
H,0,0.7416924007,16.7616397591,15.1752226497
H,0,3.1750881762,17.9704427096,15.0610861826
H,0,3.8180023678,18.3283698442,12.4378322088
H,0,0.4148352652,20.3782621381,10.9766856967
H,0,2.3801715781,21.3161960438,12.6027472
H,0,1.6298566814,20.8761567991,15.1827108088
H,0,-0.8109588869,19.6726232857,15.1518346414
H,0,-1.5643579968,19.3703778921,12.552326158
H,0,-0.5628609988,14.8847227065,5.1059307259
H,0,-1.9045214569,12.7787911736,6.2063895553
H,0,-1.4551158291,12.8166827825,8.8929753524
H,0,0.1542479627,14.9398900037,9.444859596
H,0,-1.9480141911,18.110524177,7.6763724268
H,0,-2.6050682034,16.7306402357,9.9202621539
H,0,-4.197789035,14.6432988872,9.1927654546
H,0,-4.5344441774,14.7461409522,6.494207008
H,0,-3.1492826562,16.8939886338,5.5543531518

2a^{+} (G = -2036.911998)

C,0,0.1479024929,21.8084114991,6.9391149203
C,0,-1.2622318973,22.0809161272,7.0412193804
C,0,-1.4749476000,23.4429693829,6.7081080402
C,0,-0.2104758549,24.0299916915,6.3931178250
C,0,0.7906983800,23.0315096954,6.5193497256
C,0,-0.2384065884,20.9947621439,3.6405592360
C,0,0.3775950175,22.2281569556,3.2737608782
C,0,-0.6443065404,23.2217337554,3.1870835358
C,0,-1.8895486183,22.6032284139,3.5050970289
C,0,-1.6394033354,21.2259945268,3.7883663717
C,0,0.7557530208,20.4951179719,7.1850148994
C,0,2.1937458890,20.2760839914,6.7074123595
C,0,2.9128622156,19.0720863745,7.3247923566
C,0,1.7006112458,16.9512237302,8.0016727749
C,0,0.9895987099,16.0295043233,7.2176961081
C,0,1.8694259050,17.0866464096,9.3847999099
C,0,2.0202890678,17.2556460046,10.5832678374
C,0,2.2146286194,17.4455924945,11.9685522018
C,0,1.5095986321,16.7741253474,13.0351471707
C,0,2.0013186573,17.2775341247,14.2690061369
C,0,2.9926771605,18.2659362860,13.9907422850
C,0,3.1241476112,18.3869508031,12.5808790450
C,0,-0.1308543619,19.8821719069,11.9182388156
C,0,0.7688032015,20.7720990446,12.5773396561
C,0,0.6018673885,20.6069983760,13.9863977296
C,0,-0.3999247962,19.6148587137,14.1987775338
C,0,-0.8522338929,19.1648272188,12.9206450307

C,0,0.1143193320,14.9575024495,7.6159754705
C,0,-0.7184279032,14.2034891294,6.7182455827
C,0,-1.5430522351,13.3330247807,7.4900746855
C,0,-1.2605563817,13.5732331271,8.8716204880
C,0,-0.2598824946,14.5833227194,8.9480354670
C,0,-2.4576555833,17.4051887389,7.8212526479
C,0,-2.9029254930,16.9192701736,9.0813527820
C,0,-3.8574034516,15.8855840153,8.8561717561
C,0,-3.9977623113,15.7182901928,7.4436716566
C,0,-3.1251067830,16.6584997409,6.8050924158
Fe,0,-0.5847448273,22.4317265809,5.1076347678
Fe,0,1.1791388846,18.8111434853,13.1213437992
Fe,0,-2.0179473478,15.2973717133,7.9604922862
N,0,1.1306255188,16.3826401329,5.9032328563
N,0,1.8675504983,17.4458103185,5.8235198347
N,0,2.2227182444,17.8065370462,7.0795717960
O,0,0.1294415437,19.5884678695,7.7354775252
H,0,-2.0137305691,21.3570036901,7.3222210629
H,0,-2.4331590042,23.9430859355,6.6580302841
H,0,-0.0464438765,25.0512234206,6.0759864160
H,0,1.8458562558,23.1729903787,6.3281915585
H,0,0.2696880160,20.0524260504,3.8050263818
H,0,1.4340613509,22.3880261620,3.1016879504
H,0,-0.4960133771,24.2671503408,2.9502828796
H,0,-2.8503969285,23.0981720812,3.5565925963
H,0,-2.3779027330,20.4940223602,4.0872002575
H,0,2.1683546102,20.1690078392,5.6162319950
H,0,2.7983583207,21.1669242627,6.9108471960
H,0,3.9243656948,18.9981827472,6.9142007378
H,0,2.9899362628,19.1709574166,8.4092103287
H,0,0.7460714223,16.0188286960,12.9087039022
H,0,1.6571682971,16.9831982979,15.2513965053
H,0,3.5289930203,18.8499176529,14.7264774852
H,0,3.7879402934,19.0544895178,12.0487830003
H,0,-0.2174958449,19.7511829477,10.8463118906
H,0,1.4710389375,21.4404219216,12.0965050427
H,0,1.1599177466,21.1207839891,14.7579323197
H,0,-0.7343915375,19.2432935800,15.1582830528
H,0,-1.6008217923,18.4015956070,12.7510464543
H,0,-0.6927946369,14.2905690746,5.6406874121
H,0,-2.2590314156,12.6231497190,7.0970664161
H,0,-1.7374305232,13.0918124794,9.7154949269
H,0,0.1415569355,15.0193989482,9.8520963069
H,0,-1.6912825243,18.1603121639,7.6753339144
H,0,-2.5384756240,17.2498011212,10.0454090880
H,0,-4.3658857692,15.3118508989,9.6202344728
H,0,-4.6513088200,15.0132046730,6.9467160262
H,0,-2.9892611266,16.7758364993,5.7378875285

2c (G = -1451.969990)

Fe,0,3.0938906745,4.6358895611,-0.0456028076
N,0,-0.6736066085,1.0801120063,-0.641693564
N,0,0.6632627595,2.1633940301,-1.96829635
N,0,-0.5115016978,1.5580425072,-1.9219637596
C,0,1.2752295165,2.0831297095,-0.727512272
C,0,1.328659244,5.7366418315,-0.0119160375
C,0,3.4906305315,3.1933618466,-1.4824320585
C,0,3.4392381444,6.6594876795,-0.3722354132
C,0,-1.8697690188,0.2953327027,-0.2969387736
C,0,0.4207654373,1.3805709458,0.1249825623
C,0,1.9982307018,5.826754103,1.2609546499
C,0,3.284726413,2.7532227229,0.7992083543
C,0,4.5833625236,3.333052807,0.5726927051
C,0,4.7093443767,3.6033314903,-0.8373269618
C,0,-1.7896289303,-1.1432358904,-0.8498531292

C,0,2.6031472236,2.6472103548,-0.47604973
C,0,2.2191620352,6.2493287875,-1.0215478855
C,0,3.3032464592,6.3978706568,1.0384512257
H,0,0.3406703266,5.3135908164,-0.1894603846
H,0,3.252136234,3.274215656,-2.5407495603
H,0,4.3208634233,7.0680127509,-0.8652028417
H,0,-1.9738914667,0.2936079428,0.7971477649
H,0,1.5976349124,5.5007643012,2.2203793829
H,0,2.8790781285,2.4564894177,1.7660997282
H,0,5.3261745215,3.5536028298,1.3384359919
H,0,5.5650897223,4.067270758,-1.3264913218
H,0,-0.8758639264,-1.6466217422,-0.4829277814
H,0,2.0163096769,6.2886855824,-2.0909716808
H,0,4.0624958562,6.5769676311,1.7991893822
H,0,-2.7433462869,0.8172608097,-0.7122089861
H,0,-1.697225311,-1.1089476658,-1.949971689
Fe,0,-2.4059251169,-5.0352532869,0.01385933
C,0,-2.6113989494,-5.3372604664,2.0642077093
C,0,-3.1171063364,-3.361197553,-0.9844274055
C,0,-0.5582523172,-5.1321810674,0.9745201315
C,0,-2.3334499029,-6.5840759057,1.3966705149
C,0,-3.925795413,-5.5265833363,-1.3303217415
C,0,-2.6652509355,-5.4094783974,-2.0209271512
C,0,-2.1576812754,-4.0830131057,-1.8065493226
C,0,-4.2033209906,-4.2765413929,-0.6844239947
C,0,-1.513482357,-4.4429104373,1.8043764622
C,0,-1.0655191946,-6.4574292615,0.723390136
H,0,-3.5053784057,-5.1017300249,2.6398154555
H,0,0.3744949105,-4.7206560132,0.590645732
H,0,-2.9821360158,-7.4590770741,1.3789987802
H,0,-4.541525711,-6.4237386643,-1.2774074468
H,0,-2.1674701806,-6.1999098335,-2.581573907
H,0,-1.218031247,-3.690943009,-2.1922918805
H,0,-1.4392306808,-3.4121053838,2.1491382121
H,0,-0.5872287223,-7.220516993,0.1104808113
H,0,-5.0653491093,-4.0304518755,-0.0683130981
C,0,-3.0231531105,-1.9777592851,-0.4542951372
O,0,-3.9080299986,-1.4959762261,0.2679954928
H,0,0.4971775139,1.0886294428,1.1682578684

2c^{+} (G = -1451.771724)

Fe,0,3.0891137193,4.9629444586,-0.1722301205
N,0,-0.1665125762,0.8082871705,-0.1043349548
N,0,1.150897384,1.790874679,-1.5279955324
N,0,0.0601866816,1.059023549,-1.4446108544
C,0,1.639362745,2.0347421139,-0.2544784394
C,0,1.22473884,5.9174594948,-0.6587503196
C,0,3.7875491801,3.1903348797,-1.0867007634
C,0,3.3204584042,6.8679499538,-1.0044158718
C,0,-1.3564790921,0.0393207848,0.3148631918
C,0,0.7923937181,1.4028487326,0.6619794547
C,0,1.7116362621,6.3822210845,0.6087600599
C,0,3.3568679021,3.3610031252,1.191783802
C,0,4.597836246,4.0392009016,0.9250607507
C,0,4.8646379596,3.931903605,-0.4875041113
C,0,-1.4637421983,-1.3008299255,-0.4413169341
C,0,2.864896192,2.8004128302,-0.045205954
C,0,2.2158054711,6.2090105658,-1.6542664939
C,0,3.0081479771,6.9749194082,0.3991427242
H,0,0.2925854708,5.3800444065,-0.8291085785
H,0,3.6694320336,2.9457202067,-2.1402504278
H,0,4.2323670943,7.2159409406,-1.4874612523
H,0,-1.2838734472,-0.1201773044,1.3993672666
H,0,1.1995306197,6.28593319,1.5652786884
H,0,2.8683661888,3.2888297446,2.1627917133
H,0,5.2155286583,4.5512796159,1.6612529549

H,0,5.7230107152,4.3472106713,-1.013314024
H,0,-0.5698515125,-1.9250968381,-0.2547389657
H,0,2.1540053466,5.9505204843,-2.7104402173
H,0,3.6406750615,7.4200550616,1.1657980358
H,0,-2.2520680424,0.6522717761,0.1313877272
H,0,-1.4850023609,-1.1152226012,-1.5285388196
Fe,0,-2.6482165117,-5.2482325825,-0.1550983854
C,0,-2.8231032529,-5.9364198588,1.8144592828
C,0,-3.0783584159,-3.3092959511,-0.7684372145
C,0,-0.8071832102,-5.8713520805,0.643361858
C,0,-2.7869105349,-7.0629567395,0.9201754013
C,0,-4.3115619889,-5.1620522357,-1.4740783884
C,0,-3.0896245374,-5.1306438257,-2.2314800835
C,0,-2.322867853,-3.9977813036,-1.8018669143
C,0,-4.3078754346,-4.0552740496,-0.5652510426
C,0,-1.5970590178,-5.1987881434,1.644636747
C,0,-1.5446723735,-7.0227124711,0.1990957881
H,0,-3.6401878229,-5.6757868006,2.4854094495
H,0,0.1699960761,-5.5592956068,0.2772529684
H,0,-3.5797868216,-7.7973461169,0.7843441456
H,0,-5.086305388,-5.9240666283,-1.5490189427
H,0,-2.7811961596,-5.8639406266,-2.9756818031
H,0,-1.3444314836,-3.7122848829,-2.1850948808
H,0,-1.3254395505,-4.2868116992,2.1744558579
H,0,-1.2339204627,-7.721402333,-0.5766142288
H,0,-5.0801420972,-3.7929885182,0.1547268407
C,0,-2.7270902798,-2.0704447935,-0.0150072539
O,0,-3.4457325423,-1.6543103298,0.9006712543
H,0,0.8046410714,1.3234886016,1.7452501196

$2c^{2+}$ (G = -1451.468111), triplet state

Fe,0,3.0527781449,5.1936738905,-0.2507045037
N,0,0.1129474333,0.7035162293,0.1130016496
N,0,1.2765873813,1.7664299312,-1.3871578544
N,0,0.2719507875,0.9360283471,-1.2466866095
C,0,1.7751799135,2.1030891283,-0.1372038167
C,0,1.1255665241,6.1518468656,-0.6933713452
C,0,3.7941801998,3.3738313557,-1.0963218599
C,0,3.2079338155,7.0518194751,-1.2111212435
C,0,-0.9951391582,-0.1497400145,0.5947674291
C,0,1.0238205888,1.4201031763,0.8283115589
C,0,1.6830883635,6.6883067035,0.5124159111
C,0,3.4290533679,3.6276186716,1.1839965629
C,0,4.6220760369,4.3580221427,0.8454107665
C,0,4.8501316261,4.1978757874,-0.5701551582
C,0,-1.1578072346,-1.4124619186,-0.2815901138
C,0,2.9392717809,2.9689580547,-0.0034725659
C,0,2.0635572033,6.3630449598,-1.7565307724
C,0,2.9709817369,7.2521163627,0.1984269807
H,0,0.1776328806,5.6209184278,-0.7783028581
H,0,3.6602095381,3.0684699544,-2.1324012837
H,0,4.0883718498,7.3714982995,-1.7670286635
H,0,-0.7834737808,-0.4131791491,1.6403984216
H,0,1.2213736698,6.6607743788,1.4989319741
H,0,2.9793084381,3.5750300668,2.1750169145
H,0,5.2386175839,4.9282319162,1.5389691394
H,0,5.6748012873,4.6204531802,-1.1425666084
H,0,-0.2468668244,-2.0400605049,-0.2383738931
H,0,1.939007204,6.0457456084,-2.7912368387
H,0,3.6414558132,7.7475995042,0.8994683184
H,0,-1.9250632525,0.4400950862,0.5833935159
H,0,-1.2732826927,-1.1238188626,-1.34053792
Fe,0,-2.7885509214,-5.4193223043,-0.1916376383
C,0,-3.2997333454,-6.1827626767,1.6933761076
C,0,-2.8146380653,-3.3859874897,-0.7138661472

C,0,-1.1679507659,-6.4551395279,0.7863241484
C,0,-3.3402303122,-7.2789542604,0.7594826706
C,0,-4.2663412567,-4.9314837255,-1.7001550121
C,0,-2.9945833605,-5.0372553396,-2.3551321989
C,0,-2.0989215761,-4.0955868455,-1.7562340168
C,0,-4.1658800512,-3.9268447984,-0.6803569718
C,0,-1.9489670295,-5.6700417449,1.709961354
C,0,-2.0272218166,-7.4447128977,0.2066128367
H,0,-4.1354215971,-5.8123514558,2.2858877492
H,0,-0.112975677,-6.3135093638,0.5530815845
H,0,-4.2215034346,-7.8633696146,0.4955477823
H,0,-5.1468411442,-5.5354778008,-1.9189017567
H,0,-2.7386563506,-5.7492272249,-3.1401811228
H,0,-1.0531443467,-3.9590391846,-2.0302554741
H,0,-1.5877370682,-4.8449548439,2.322363578
H,0,-1.7448505747,-8.165618867,-0.5607650756
H,0,-4.9564298489,-3.5887674291,-0.0123581964
C,0,-2.3710835113,-2.2371788161,0.1636970907
O,0,-3.0215416797,-1.958164105,1.1711261607
H,0,1.0804255583,1.3781510517,1.912651624
